# *Lactobacillus rhamnosus* GG attenuates MASLD/MASH progression by modulating gut microbiota and metabolic pathways

**DOI:** 10.3389/fmicb.2025.1586678

**Published:** 2025-07-24

**Authors:** Shi-Long Wang, Si Liang, Si-Yu Li, Jin-Wen Fu, Zi-Yi-Ru Wang, Dong-Qing Zhu, Nan Chen

**Affiliations:** ^1^School of Basic Medical Sciences, Hebei University, Baoding, China; ^2^Department of Anesthesiology, Affiliated Hospital of Hebei University, Baoding, China; ^3^Clinical Medical College, Hebei University, Baoding, China

**Keywords:** non-alcoholic fatty liver disease, non-alcoholic steatohepatitis, probiotics, gut microbiota, gut barrier

## Abstract

**Introduction:**

Non-alcoholic fatty liver disease (MASLD) is a common liver condition with a global prevalence of approximately 25%, often associated with overweight, obesity, and abnormalities in glucose and lipid metabolism. Its histological hallmark is hepatic steatosis. Non-alcoholic steatohepatitis (MASH), an advanced form of MASLD, can lead to cirrhosis and liver cancer. Dysbiosis of the gut microbiota plays a significant role in chronic liver diseases, making probiotic treatment a focal point in MASLD research. Studies have shown that *Lactobacillus rhamnosus* GG (LGG) can improve gut microbiota, reduce hepatic fat accumulation, and lower blood lipid levels in MASLD model mice. However, the role of LGG in the progression from MASLD to MASH remains unclear.

**Methods:**

In this study, we constructed MASLD and MASH models using a high-fructose, high-fat diet combined with carbon tetrachloride (CCl4) induction to explore the effects of LGG on disease progression. Our findings revealed that in the MASLD model, LGG improved lipid metabolism and inflammatory responses by modulating the gut microbiota (e.g., increasing the abundance of Bacteroidetes) and promoting the production of short-chain fatty acids (SCFAs). Additionally, LGG reduced the expression of genes related to lipogenesis, further alleviating MASLD.

**Results:**

In the MASH model, LGG primarily exerted its effects by inhibiting the TGF-β/SMAD signaling pathway and reducing the expression of pro-inflammatory factors (e.g., IL-1β, IL-6, TNF-α), thereby mitigating liver fibrosis and inflammation. Furthermore, LGG restored intestinal barrier function, reduced intestinal permeability, and prevented harmful substances like endotoxins from entering the liver, further alleviating hepatic inflammation and fibrosis.

**Discussion:**

Although LGG shows promise in the treatment of MASLD and MASH, its mechanisms of action and long-term effects require further investigation. Future research should focus on optimizing the types, dosages, and treatment regimens of probiotics, as well as monitoring their long-term impact on gut microbiota balance, to ensure their safety and efficacy in clinical applications.

## 1 Introduction

Metabolic dysfunction-associated steatotic liver disease (MASLD) is a common liver disease that poses a significant threat to human health, with a global prevalence of approximately 25%. Patients with MASLD often present with overweight or obesity, as well as various glucose and lipid metabolism abnormalities such as diabetes and hyperlipidemia. The histological changes in MASLD are primarily characterized by hepatic steatosis, without accompanying hepatocyte degeneration, necrosis, injury, or fibrosis (Yan et al., 2024). Metabolic dysfunction-associated steatohepatitis (MASH) is an advanced form of MASLD, manifesting as steatohepatitis and steatotic cirrhosis. Ultimately, it may lead to the development of cirrhosis and hepatocellular carcinoma, making it one of the primary causes of end-stage liver disease (Sarkar et al., 2023).

Gut microbiota dysbiosis is prevalent in chronic liver diseases and plays a crucial role in the onset and progression of cirrhosis. Probiotic therapy has thus become a hot topic in the treatment of MASLD in recent years (Zhai et al., 2023). Studies have shown that an 8-week high-fructose diet can induce experimental MASLD in C57BL/J6 mice, and the administration of *Lactobacillus rhamnosus* GG (LGG) increased beneficial bacteria in the distal small intestine, reduced duodenal IκB protein levels, and restored duodenal tight junction protein concentrations (Higarza et al., 2021). Another study demonstrated that a 13-week high-fat diet (HFD) induced MASLD in mice, and daily LGG gavage significantly reduced liver, mesenteric, and subcutaneous adipose tissue weights, as well as serum triglyceride and cholesterol levels, while also restoring HFD-induced gut microbiota dysbiosis (Kim et al., 2016). A recent study indicated that live LGG effectively prevented high-fat, high-fructose diet-induced MASLD in rats, significantly reducing hepatic fat accumulation. This effect was primarily mediated by reducing lipid uptake and accelerating the release of hepatic triglycerides into the bloodstream (Lu et al., 2024).

However, the therapeutic role of LGG in the progression from MASLD to MASH remains unclear. Some studies have shown that a short-term high-fat, high-fructose diet combined with carbon tetrachloride (CCl_4_) induction can shorten the time required for MASH induction, exacerbate liver fibrosis, and maintain other pathological changes such as hepatic lipid accumulation, steatosis, ballooning, and inflammation (Hammerich and Tacke, 2023). The pathological process of MASH induced by HFD combined with CCl_4_ in mice closely resembles the clinical progression of MASH (Araujo et al., 2024). Therefore, in this study, we used a high-fat, high-fructose diet combined with weekly intraperitoneal injections of CCl_4_ to establish an MASLD model, and a high-fat, high-fructose diet combined with twice-weekly intraperitoneal injections of CCl_4_ to establish an MASH model, aiming to explore the role of LGG in the progression of MASLD and MASH.

## 2 Materials and methods

### 2.1 Cultivation of LGG

The *Lactobacillus rhamnosus* GG ATCC6141 was purchased from ATCC and stored at −80°C. The pre-frozen LGG strain is retrieved from the −80°C freezer and placed at room temperature to fully thaw. Next, the freeze-dried tube is opened near an alcohol lamp, and the bacterial solution inside is thoroughly mixed using a pipette. The bacterial solution is then inoculated into sterilized MRS broth at a 2% concentration. The inoculated MRS broth is placed in a biochemical incubator and cultured at 37°C for 48 h to complete the recovery of the LGG strain. After the bacterial strain revival is complete, to activate LGG, a 2% inoculum of the recovered MRS broth should be aspirated from the MRS broth and inoculated into new sterilized MRS broth in a laminar flow cabinet. The inoculated broth is then placed in an incubator and cultured at 37°C for 24 h. To ensure the full activation of the LGG strain, this process should be repeated three times using the same method. After activation, the LGG strain was cultured again. Next to an alcohol lamp, the bacterial solution was aspirated from the activated MRS broth with a 2% inoculation amount and inoculated into a new sterilized MRS broth, which was then incubated at 37°C in a biochemical incubator for 24 h. Count the recovered LGG bacterial suspension. In a laminar flow cabinet, mix the bacterial suspension by shaking it evenly. Use a pipette to transfer 100 μL of the bacterial suspension into 900 μL of sterilized saline for gradient dilution. Select three dilutions: 10^6^ CFU/mL, 10^7^ CFU/mL, and 10^8^ CFU/mL. Take 100 μL from each dilution and evenly spread it on the surface of MRS solid agar. Before spreading, test the temperature of the sterilized glass spreader at the edge of the plate to prevent scalding the bacteria. After spreading, let the bacterial suspension fully absorb for 20 min, then invert the petri dish and incubate it in a 37°C constant temperature incubator for 16 h. Set three duplicate samples for each dilution. Determine the original bacterial concentration using the CFU counting method. The final count is approximately 3 × 10^9^ CFU/mL. In the super-clean workbench, after thoroughly mixing the re-cultured bacterial solution, 5 mL of the bacterial solution is transferred to a 15 mL sterile centrifuge tube. The mixture is then centrifuged at 4°C and 4,000 rpm for 10 min to separate the bacterial cells from the supernatant. After collecting the bacterial cells, they are resuspended in physiological saline to re-suspend LGG live bacteria. The concentration of LGG live bacteria during the logarithmic growth phase, calculated using the plate dilution method, is approximately 3 × 10^9^ CFU/mL, with a cultivation time of 16 h. For subsequent experiments, the LGG live bacteria cultured over 16 h will be used as the gastric feeding bacterial solution.

### 2.2 Animal handling

Seven-week-old male C57BL/6 J mice (purchased from Beijing Vital River Laboratory Animal Technology Co, Ltd.) were housed under controlled environmental conditions (temperature 23 ± 1°C, humidity 50–60%, 12-h light/dark cycle) with ad libitum access to food and water. The body weight and food intake of the mice were monitored weekly. All animal experimental procedures were approved by the Institutional Animal Care and Use Committee of Hebei University (Animal Ethics Number: HBU2024M030) and conducted in accordance with the standards outlined in the Guide for the Care and Use of Laboratory Animals, with humane care provided. After a one-week acclimation period, the mice were randomly divided into six groups: (1) The control group received a normal diet and intraperitoneal injections of olive oil (once a week), with tap water as the drinking source. Within this group, the LGG drug control group (LGG) received daily oral gavage of 1 × 10^9^ CFU live LGG (200 μL), while the blank control group (Con) received daily oral gavage of 200 μL of saline. (2) The model groups were fed a high-fat diet and given sugar water to drink (18.9 g/L d-fructose and 3.4 g/L d-glucose), with daily oral gavage of 200 μL of saline. Among these, the MASLD model group (Steatosis, STE) received intraperitoneal injections of CCl_4_ once a week (10%, diluted in olive oil), and the MASH model group (Fibrosis, FIB) received intraperitoneal injections of CCl_4_ twice a week (10%, diluted in olive oil). (3) The treatment groups, based on the model groups, received daily oral gavage of 1 × 10^9^ CFU live LGG (200 μL), divided into the MASLD treatment group (STE + LGG) and the MASH treatment group (FIB+LGG). CCl_4_ Concentration and frequency of intraperitoneal injection were determined according to previous literature methods and pre-experiment (Tsuchida et al., 2018). At the end of the 12th week, mice were euthanized by exsanguination from the orbital plexus followed by cervical dislocation. Whole blood was centrifuged at 2,000 g for 15 min at 4°C. The supernatant (serum) was stored at −80°C. The liver and epididymal adipose tissue were dissected and weighed, with some organs frozen in liquid nitrogen and stored at −80°C for subsequent experiments.

### 2.3 Histopathological analysis

Liver and colon tissues were fixed in 4% paraformaldehyde for 48 h and subjected to histopathological analysis. After dehydration, embedding, and sectioning, liver tissues were stained with hematoxylin and eosin (H&E), Oil Red O, and Masson’s trichrome, while colon tissues were stained with H&E. Sections were scanned using a Nikon optical microscope and imaging system (Nikon, Japan), and liver MASLD/MASH disease activity scores were determined in a blinded manner based on the pathological results of H&E staining. ImageJ software was used to quantify Oil Red O-stained and Masson’s trichrome-stained positive areas, as well as to measure and count colon goblet cells and crypt depths.

### 2.4 Measurement of serum biochemical indicators

Biochemical contents of total aspartate aminotransferase (AST/GOT)(Cat# C010-2-1) and alanine aminotransferase (ALT/GPT) (Cat# C009-2-1) in serum and liver were determined using ALT and AST biochemical kits (Nanjing Jiancheng Bioengineering Institute, China). The operating procedures followed the instructions provided with the kits.

### 2.5 ELISA assays

ELISA kits (Wuhan Sanying Biotechnology, China) were used to measure IL-1β (Proteintech, Cat# KE10003) and TNF-α (Proteintech, Cat# KE10002) concentrations in liver tissues. The operating procedures followed the instructions provided with the kits.

### 2.6 Real-time fluorescent quantitative polymerase chain reaction

RNA was extracted from mouse liver and colon tissues using a tissue RNA extraction kit (TIANGEN BIOTECH, Beijing), and its concentration was detected using a NanoDrop 2000/2000c spectrophotometer. cDNA was synthesized using 2 μg of RNA and TB Green^®^ Premix Ex Taq^™^ II (Takara, Japan) was used for qPCR. The primer sequences used in this study are shown in Supplementary Table S1. Relative gene expression levels were normalized to GAPDH and calculated using the 2^−ΔΔCt^ method.

### 2.7 Western blot analysis

Total protein was extracted from liver and colon tissues using PMSF and RIPA, followed by centrifugation at 12,000 rpm for 10 min at 4°C. The protein concentration in the supernatant was then determined using a BCA protein assay kit (Biomed, Beijing). Protein samples were heated at 100°C for 10 min in 5 × protein loading buffer. After separation by 10% SDS-PAGE electrophoresis, the target proteins were transferred onto PVDF membranes. The membranes were then blocked in a solution of 5% skimmed milk powder at room temperature for 1 h and incubated with primary antibodies overnight at 4°C. The primary antibodies were Occludin Polyclonal antibody (1:1000, 27260-1-AP, Proteintech Group), Claudin-1 Polyclonal antibody (1:2500, 28674-1-AP, Proteintech Group), TGF Beta 1 Polyclonal antibody (1:1000, 21898-1-AP, Proteintech Group), smooth muscle actin Polyclonal antibody (1:1500, 14395-1-AP, Proteintech Group), Collagen Type I Polyclonal antibody (1:1000, 114695-1-AP, Proteintech Group), SMAD2 Polyclonal antibody (1:1500, 12570-1-AP, Proteintech Group) and GAPDH Polyclonal antibody (1:2000, 10494-1-AP, Proteintech Group). After washing with 1 × TBST four times for 5 min each, the membranes were incubated with HRP-conjugated Goat Anti-Rabbit IgG(H + L) (1:2000, SA00001-2, Proteintech Group) at room temperature for 2 h. The membranes were then washed as described earlier. Finally, protein bands were detected using an ECL chemiluminescent detection kit (Biosharp, China) and an ECL blotting detection system (Tanon, China). The grayscale values of the immunoblotting bands were analyzed using ImageJ software.

### 2.8 Immunofluorescence analysis

Colon paraffin sections were dewaxed and washed. The primary antibody (anti-Occludin antibody) was incubated overnight at 4°C, followed by incubation with the secondary antibody at room temperature for 50 min. The cell nuclei were stained with DAPI, and the autofluorescence of the tissue was quenched using an autofluorescence quenching reagent. The sections were then sealed with an anti-fluorescence quenching reagent and observed and photographed under a microscope (Nikon Eclipse C1, Japan). The Occludin protein emitted red fluorescence, and the cell nuclei emitted blue fluorescence. The immunofluorescence intensity was analyzed using ImageJ software.

### 2.9 Extraction of total microbial DNA and metagenomic sequencing

Fresh intestinal contents were collected and immediately frozen at −80°C for sequencing. Total genomic DNA extraction from microbial communities was performed according to the instructions of the E. Z. N. A.^®^ soil DNA kit (Omega Bio-tek, Norcross, GA, United States). The quality of the extracted genomic DNA was checked using 1% agarose gel electrophoresis, and the concentration and purity of the DNA were measured using a NanoDrop 2000 (Thermo Scientific, United States). Using the extracted DNA as a template, the V3-V4 variable region of the 16S rRNA gene was amplified by PCR using upstream primer 338F and downstream primer 806R, both carrying Barcode sequences. The purified PCR products were used to construct libraries using the NEXTFLEX Rapid DNA-Seq Kit, and sequencing was performed on the Illumina PE300/PE250 platform (Shanghai Majorbio Bio-pharm Technology Co., Ltd.). All data analysis was conducted on the Majorbio Cloud Platform.

### 2.10 Statistical analysis

In addition to the metagenomic analysis described above, another statistical analysis was performed using GraphPad Prism 10.0 (GraphPad Software, San Diego, CA, United States). Results are presented as mean ± standard deviation (SD). One-way ANOVA was used for multiple group comparisons, and the *t*-test was used for comparisons between two groups. Differences were considered statistically significant at *p* < 0.05.

## 3 Results

### 3.1 LGG ameliorates MASLD induced by HFD in mice

**Figure 1:**
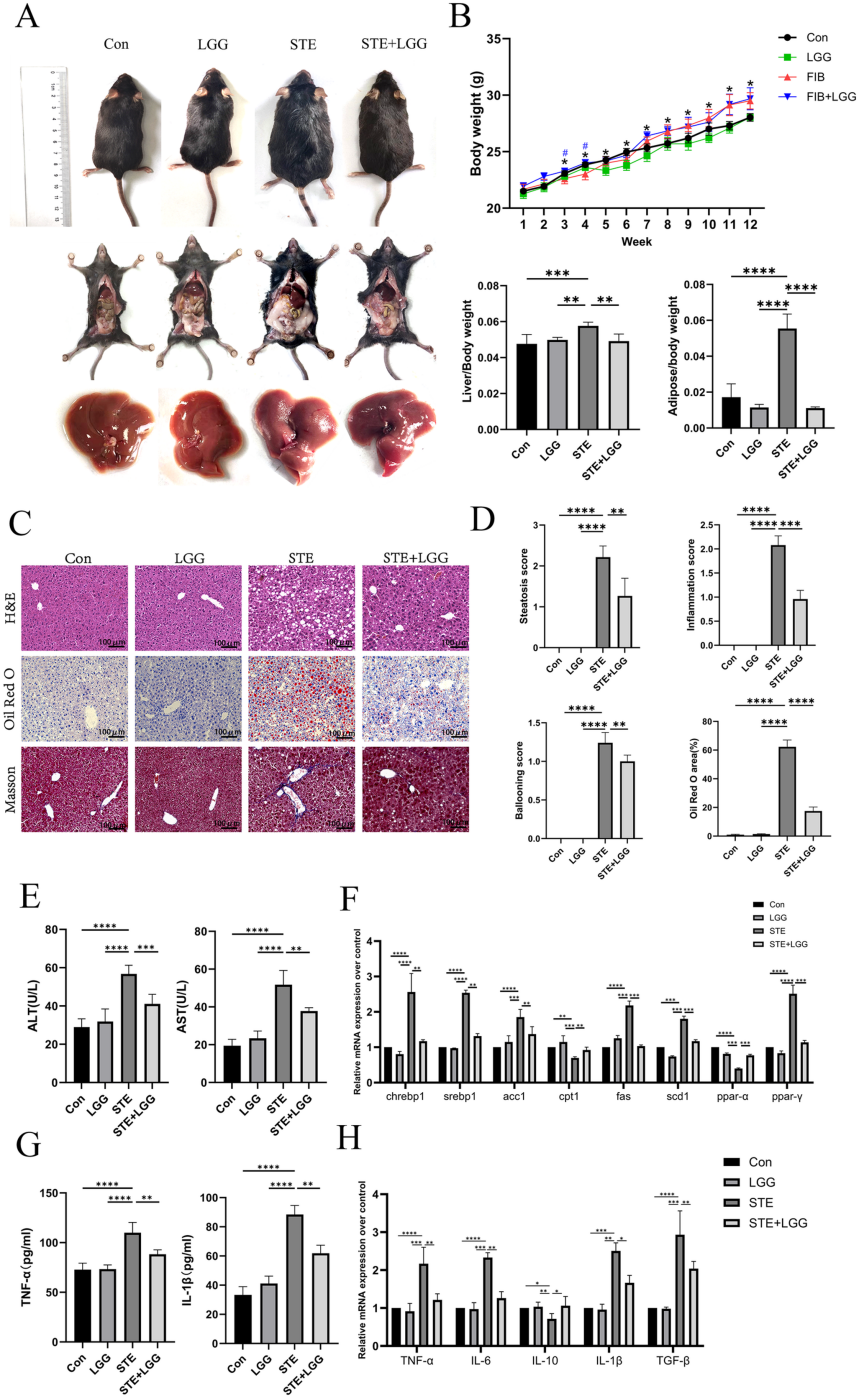
LGG attenuates MASLD induced by HFD in mice. **(A)** Comparison of body size, epididymal fat and liver appearance of mice in each group. **(B)** Changes in body weight of mice in each group and liver index and epididymal fat index of mice in each group. **(C)** H&E, oil Red O and Masson staining images of mouse liver. **(D)** Steatosis score, inflammation score, ballooning degeneration score and quantitative oil red-O staining. **(E)** Serum ALT level and AST level. **(F)** mRNA expression levels of liver-related lipid metabolism factors. **(G)** The levels of pro-inflammatory factors IL-1β and TNF-α in the liver. **(H)** The mRNA expression levels of liver-related inflammatory factors TNF-α, IL-6, IL-10, and TGF-β. Each value is presented as mean ± SD (*n* = 6). *p < 0.05, **p < 0.01, ***p < 0.001, ****p < 0.0001.

To investigate the role of LGG in the treatment of MASLD/MASH, we subjected mice to a high-sugar, high-fat diet for 12 weeks and recorded their body weight daily. On the last day of the 12th week, the mice were euthanized, and their livers and epididymal adipose tissues were weighed. The results showed that due to the 12-week HFD, mice in the STE group became more obese, with wider distribution of epididymal fat. In contrast, mice treated with LGG had smaller body sizes and less epididymal fat distribution. Upon examination of liver tissue, we found that the livers of mice in the control and LGG groups had smooth surfaces, soft textures, and sharp edges. However, in the MASLD model, due to long-term fat infiltration, the liver volume increased, the edges became blunt, and the texture hardened. After LGG treatment, the liver became smoother and its volume decreased (Figure 1A). Consistent with the changes in body shape, LGG also significantly inhibited HFD-induced weight gain and significantly improved liver and fat indices in mice (Figure 1B).

The accumulation of fat in the liver is one of the characteristics of MASLD. To evaluate the impact of LGG on HFD-induced liver pathology and lipid accumulation, we used H&E and Oil Red O staining. The results showed that hepatocytes in the control group were neatly arranged with clear liver cords and no obvious lipid deposition. In contrast, hepatocytes in the STE group were disordered, with fat vacuoles of varying sizes and intralobular inflammatory foci. LGG treatment significantly reduced liver inflammatory infiltration and lipid deposition. Oil Red O staining revealed severe hepatic steatosis in the STE group, with large fat droplets occupying most of the area, accounting for up to 60%. After LGG treatment, this value decreased to 18% (Figures 1C,D). These findings suggest that LGG can reduce body fat and protect the liver, thereby alleviating the progression of MASLD.

In MASLD mice, elevated levels of ALT and AST usually indicate liver cell damage, which may be caused by inflammation due to fat accumulation in the liver. Therefore, we determined the levels of ALT and AST in mouse serum using biochemical kits. The results showed that compared with the STE group, LGG treatment reduced serum ALT concentration from 59 U/L to 42 U/L and serum AST concentration from 53 U/L to 34 U/L (Figure 1E). ACC, FAS, and SCD1 are considered key enzymes regulating fatty acid metabolism, while CNREBP1 and SREBP1c are transcription factors for lipogenesis genes. Consistent with the reduction in liver lipid accumulation, we found that the gene expression of these key lipid metabolism genes (ACC, FAS, SCD1, CHREBP1, SREBP1) was significantly upregulated in the STE group, the increase of CPT1 expression can accelerate the transport of fatty acids to mitochondria, enhance β-oxidation, and reduce the accumulation of triglycerides in hepatocytes, thus alleviating steatosis. In the STE group, we found that CPT1 gene expression was downregulated, after LGG treatment, the expression of genes related to lipogenesis (ACC, FAS, SREBP1, and SCD1) in the livers of MASLD mice was significantly reduced, and the expression of CPT1 gene increased (Figure 1F). The concentrations of pro-inflammatory cytokines TNF-*α* and IL-1β were determined by ELISA, revealing high concentrations of TNF-α (88 Pg/ml) and IL-1β (117 Pg/ml) in the STE group. After LGG treatment, the concentrations of TNF-α and IL-1β decreased significantly to 61 Pg/ml and 82 Pg/ml, respectively (Figure 1G). Compared with the STE group, LGG treatment significantly reduced the gene expression of pro-inflammatory cytokines TNF-α, IL-1β, IL-6, and TGF-β and increased the secretion of the anti-inflammatory cytokine IL-10 (Figure 1H).

In conclusion, LGG can significantly participate in fat metabolism in MASLD mice, including lipid oxidation in adipose tissue and the liver. In turn, increased lipid oxidation reduces fat accumulation in metabolic tissues. Reduced lipid load in adipose tissue may have a positive impact on liver lipid accumulation.

### 3.2 LGG ameliorates HFD-induced intestinal barrier damage in MASLD mice

**Figure 2:**
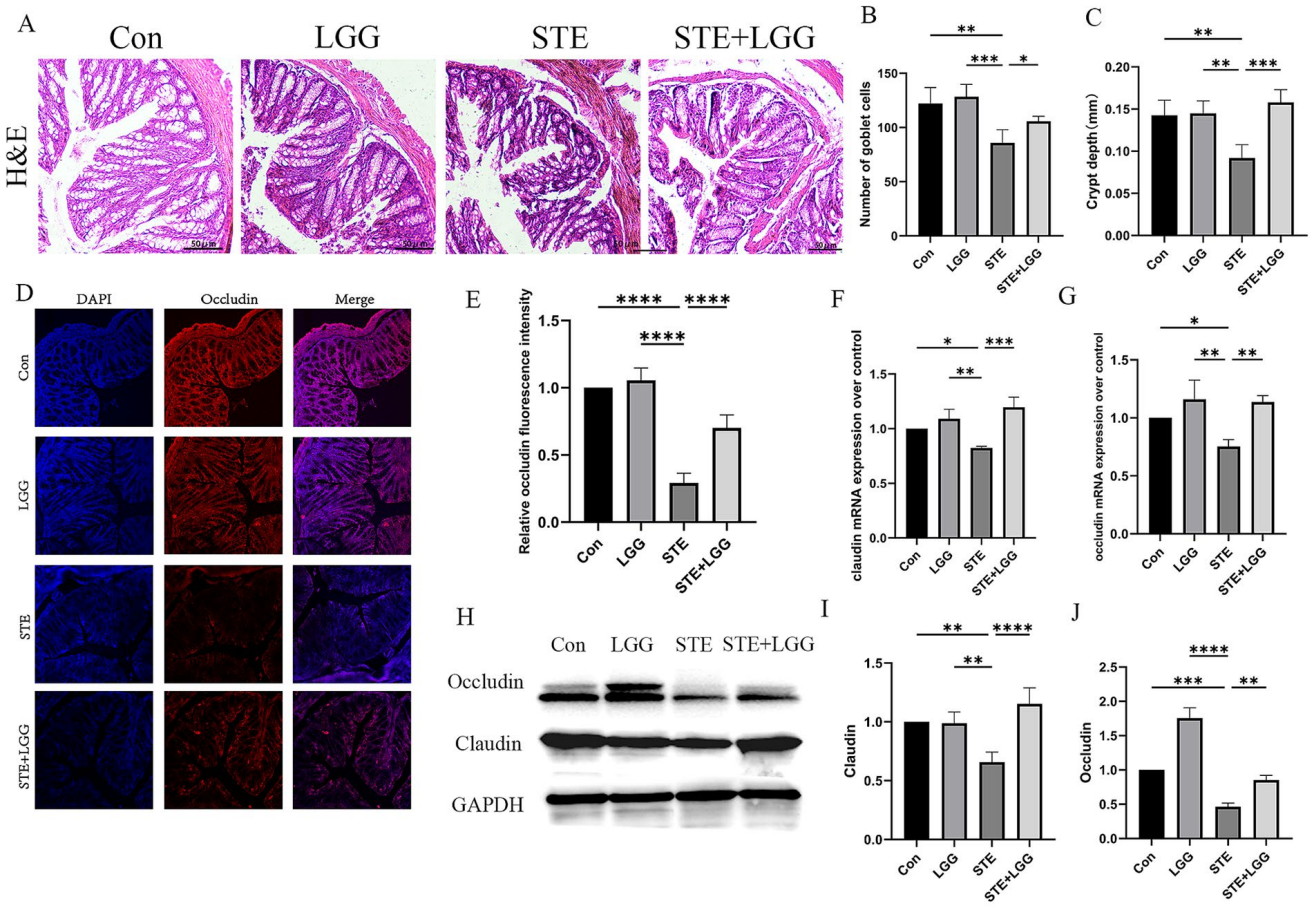
LGG ameliorates HFD-induced intestinal barrier damage in MASLD mice. **(A)** H&E staining images of mouse colonic segments. **(B)** Goblet cell counts in mouse colons. **(C)** Crypt depth in mouse colons. **(D)** Immunofluorescence images of Occludin in mouse colons. Red fluorescence represents Occludin expression, and blue fluorescence is DAPI-stained nuclei. **(E)** Quantification of Occludin fluorescence intensity in mouse colons. **(F,G)** mRNA levels of tight junction proteins claudin and occludin. **(H)** Western blot analysis of tight junction proteins Occludin and Claudin, with GAPDH as a control. **(I,J)** Quantification of western blot results. Each value is presented as mean ± SD (*n* = 6). *p < 0.05, **p < 0.01, ***p < 0.001, ****p < 0.0001.

Disruption of the intestinal barrier may expose the liver to additional bacteria or bacterial metabolites, such as LPS, leading to persistent inflammatory responses and promoting liver injury. Therefore, based on the gut-liver axis theory, we speculate that LGG may have a potential therapeutic effect on intestinal permeability in MASLD/MASH mice. H&E staining was used to assess the degree of colonic damage. Compared to the control group, the STE group showed significant changes such as epithelial cell shedding, goblet cell reduction, and shallowing of crypt depth in colonic tissue. By counting, the number of goblet cells per field of view decreased from 122 to 79, and the average crypt depth shallowed from 0.14 mm to 0.09 mm. After LGG treatment, the number of goblet cells per field of view recovered to 102, and the average crypt depth also recovered to 0.16 mm. LGG significantly alleviated the above intestinal pathological changes (Figures 2A–C). Immunofluorescence was used to detect Occludin protein expression, and it was found that Occludin was evenly distributed in red in the colonic tissue of the control group, while it was significantly weakened and sparsely distributed in the model group compared to the control group (Figures 2D,E). Consistent with the immunofluorescence results, QPCR and western blot results also showed that the expression of Occludin and Claudin was significantly reduced in the STE group, leading to intestinal barrier damage (Figures 2F–J). LGG treatment increased the expression of Occludin and Claudin, enhanced the intestinal barrier, thereby reducing bacterial translocation and alleviating the occurrence of MASLD inflammation.

### 3.3 LGG ameliorates microbial dysbiosis in HFD-induced MASH

**Figure 3:**
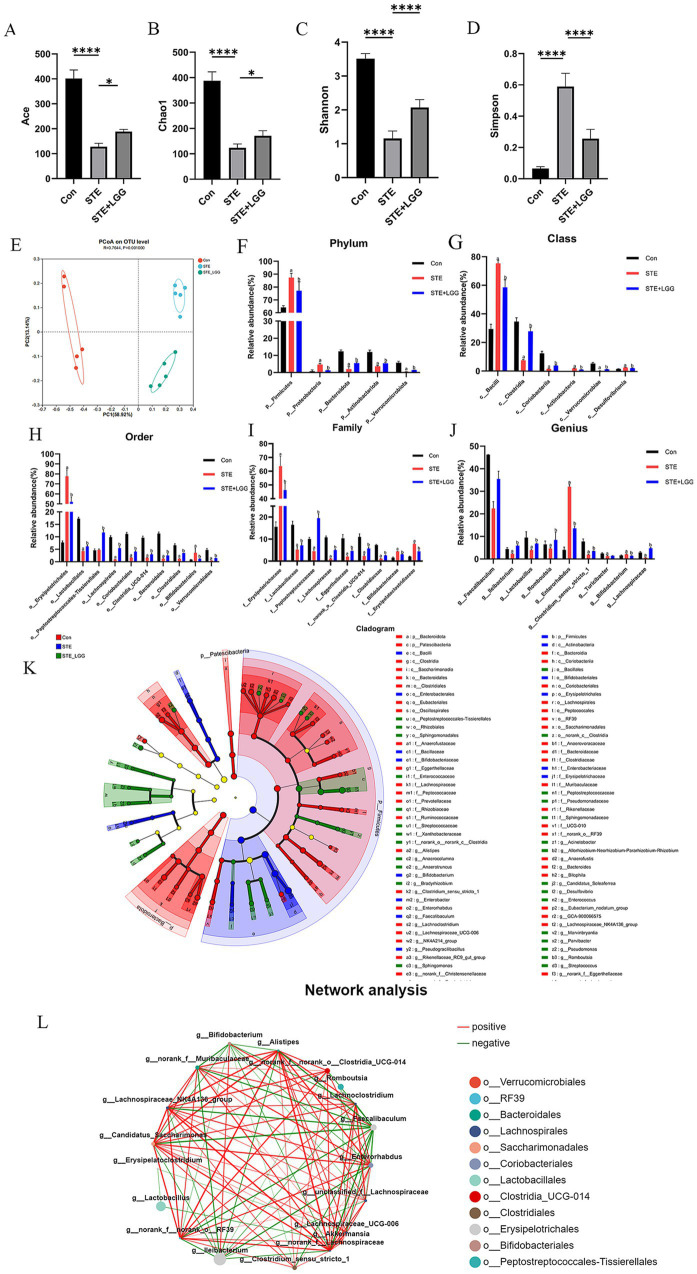
LGG ameliorates microbial dysbiosis in MCD-induced MASH. **(A–D)** Comparison of intestinal content flora α-diversity indices Ace, Chao1, Shannon, and Simpson. **(E)** PCoA analysis. **(F)** Phylum-level species composition. **(G)** Class-level species composition. **(H)** Order-level species composition. **(I)** Family-level species composition. **(J)** Genus-level species composition. **(K)** Identification of differentially enriched specific bacterial taxa among groups by LEfSe analysis. **(L)** Relative abundance of differentially abundant bacterial groups identified at the genus level. Each value is presented as mean ± SD (*n* = 6). *p < 0.05, **p < 0.01, ***p < 0.001, ****p < 0.0001.

To assess the impact of LGG treatment on intestinal microbiota diversity, we conducted 16S rRNA sequencing analysis on bacteria extracted from cecal contents of mice in each group. In terms of *α*-diversity indices, LGG treatment significantly increased the Ace index, Chao-1, and Shannon index, and significantly decreased the Simpson index, indicating that HFD-induced intestinal microbial dysbiosis was improved (Figures 3A–D). Similarly, PCoA results also showed significant differences in Beta diversity of intestinal microbiota among the three groups (Figure 3E). At the phylum level, Firmicutes and Bacteroidetes accounted for about 90% of the total flora. The Firmicutes/Bacteroidetes (F/B) ratio was significantly increased in the STE group, while LGG treatment reversed this ratio (Figure 3F). At the class level, the differential abundance of *Bacilli* was increased, and the differential abundance of *Clostridia*, *Coriobacteriia*, and *Verrucomicrobiae* was decreased in the STE group. LGG treatment reversed these changes (Figure 3G). At the order level, the abundance of *Erysipelotrichales* was significantly increased, while the abundance of *Lactobacillales*, *Lachnospirales*, *Coriobacteriales*, *Clostridia*, and *Bacteroidales* was decreased in the STE group. LGG treatment reversed these changes (Figure 3H). At the family level, the abundance of *Erysipelotrichaceae* and *Bifidobacteriaceae* was increased, while the abundance of *Lactobacillaceae*, *Lachnospiraceae*, *Eggerthellaceae*, and *Clostridiaceae* was decreased in the STE group. LGG treatment reversed these changes (Figure 3I). At the genus level, the differential abundance of *Faecalibaculum*, *Lactobacillus*, *Clostridium*, and *Lachnospiraceae* was decreased, while the differential abundance of *Enterorhabdus* was increased in the STE group. LGG treatment reversed these changes (Figure 3J). To further elucidate the differences in the abundance of intestinal microbiota, Linear Discriminant Analysis Effect Size (LEfSe) in genus-level analysis showed multiple differentially abundant microbiota (Figure 3K). In addition, univariate correlation network analysis of samples revealed that 12 strains were significantly negatively correlated with Erysipelotrichaceae in the cecum, including three intestinal beneficial strains: *Lactobacillus reuteri*, Lachnospiraceae, and *Akkermansia muciniphila* (Figure 3L). *Lactobacillus reuteri* can induce the production of a type of immune cell that promotes tolerance through the tryptophan derivative 3-indole-acetic acid. Lachnospiraceae can participate in the metabolism of various carbohydrates and ferment to produce acetic acid and butyric acid. *Akkermansia muciniphila* can repair intestinal mucosa, regulate immunity, and limit the onset of inflammation. Our results demonstrate that LGG can alleviate MASLD caused by HFD by reversing intestinal microbial dysbiosis and participating in the regulation of intestinal flora metabolism.

### 3.4 LGG attenuates the progression from MASLD to MASH in mice

**Figure 4:**
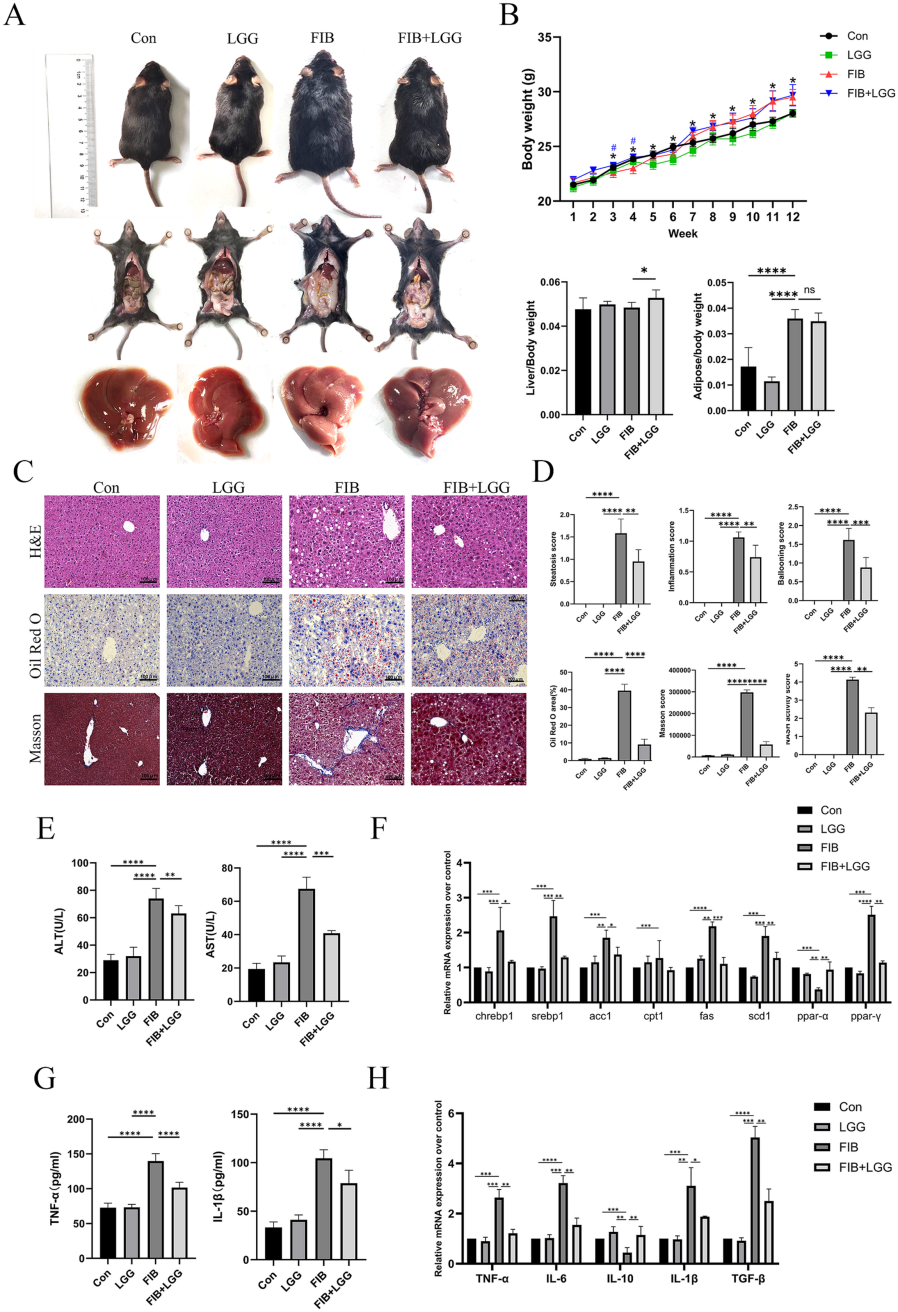
LGG attenuates the progression from MASLD to MASH in mice. **(A)** Comparison of body shape, epididymal fat, and liver appearance among mice in different groups. **(B)** Body weight, liver index, and epididymal fat index of mice in each group. **(C)** H&E, Oil Red O, and Masson staining images of mouse livers. **(D)** Scores for steatosis, inflammation, ballooning, quantitative analysis of Oil Red O and Masson staining, and MASH disease score. **(E)** Serum levels of ALT and AST. **(F)** mRNA expression levels of liver-related lipid metabolism factors. **(G)** Levels of pro-inflammatory cytokines TNF-α and IL-1β in the liver. **(H)** mRNA expression levels of liver-related inflammatory cytokines TNF-α, IL-6, IL-10, and TGF-β. Each value is presented as mean ± SD (*n* = 6). *p < 0.05, **p < 0.01, ***p < 0.001, ****p < 0.0001.

The results showed that in the MASH model, due to hepatocyte damage and fibrous tissue hyperplasia, the liver surface of mice exhibited obvious granularity, with an overall reduction in liver volume, hardening texture, and blunted edges. LGG treatment significantly reduced the granularity and softened the liver texture (Figure 4A). However, unlike the MASLD model, LGG intervention cannot significantly reduce the weight gain induced by HFD in mice. Specifically, at the end of the experiment, both the model group and the LGG intervention group showed significantly higher body weights compared to the control group, but there was no statistically significant difference between the two groups. Additionally, the improvement in liver and fat indices by LGG intervention was also not significant (Figure 4B). To clarify the therapeutic effect of LGG on liver lesions caused by HFD + CCl_4_-induced MASH, we employed H&E, Oil Red O staining, and Masson staining. H&E staining images revealed that hepatocytes in the control group were neatly arranged with clear liver cords and no obvious lipid deposition. In contrast, hepatocytes in the FIB group were disordered, with more extensive steatosis accompanied by obvious intralobular inflammatory foci and ballooning changes. After LGG treatment, steatosis, inflammatory infiltration, and ballooning changes in the liver were significantly reduced. Oil Red O staining showed steatosis in the FIB group with partial fatty droplet areas, and LGG treatment reduced the deposition of red lipid droplets. Masson trichrome staining showed increased collagen deposition in the liver of the FIB group, with portal-periportal bridging fibrosis due to CCl_4_ intervention. LGG treatment significantly reduced collagen deposition and alleviated fibrosis. MASH scoring of liver sections from mice in each group revealed that the NAS score in the FIB group reached 4.1 points, while the NAS score after LGG treatment decreased to 2.4 points (Figures 4C,D). These results suggest that in the MASH disease model, LGG may be more likely to reduce the progression of MASH by improving liver fibrosis deposition, but the specific mechanism of action remains to be further studied.

The levels of ALT and AST in mouse serum were determined using biochemical kits. The results showed that compared with the FIB group, the serum ALT concentration decreased from 72 U/L to 60 U/L and the serum AST concentration decreased from 68 U/L to 41 U/L after LGG treatment (Figure 4E). Consistent with the above MASLD findings, we found that the expression of these key lipid metabolism genes was significantly upregulated in the MASH model, and LGG treatment significantly reduced the genes related to lipogenesis, including ACC, FAS, SREBP1c, and SCD1, in the livers of MASH mice (Figure 4F). We further investigated the effect of LGG on liver inflammation in MASH mice by determining the concentrations of pro-inflammatory cytokines TNF-α and IL-1β using ELISA. The results showed that the concentration of TNF-α was as high as 137 Pg/ml and IL-1β was 107 Pg/ml in the FIB group, and after LGG treatment, the concentrations of TNF-α and IL-1β decreased significantly to 102 Pg/ml and 76 Pg/ml, respectively, (Figure 4G). Compared with the FIB group, LGG treatment significantly reduced the secretion of pro-inflammatory cytokines TNF-α, IL-1β, IL-6, and TGF-β but increased the gene expression of the anti-inflammatory cytokine IL-10 (Figure 4H).

### 3.5 LGG improves intestinal barrier damage in HFD-induced MASH mice

**Figure 5:**
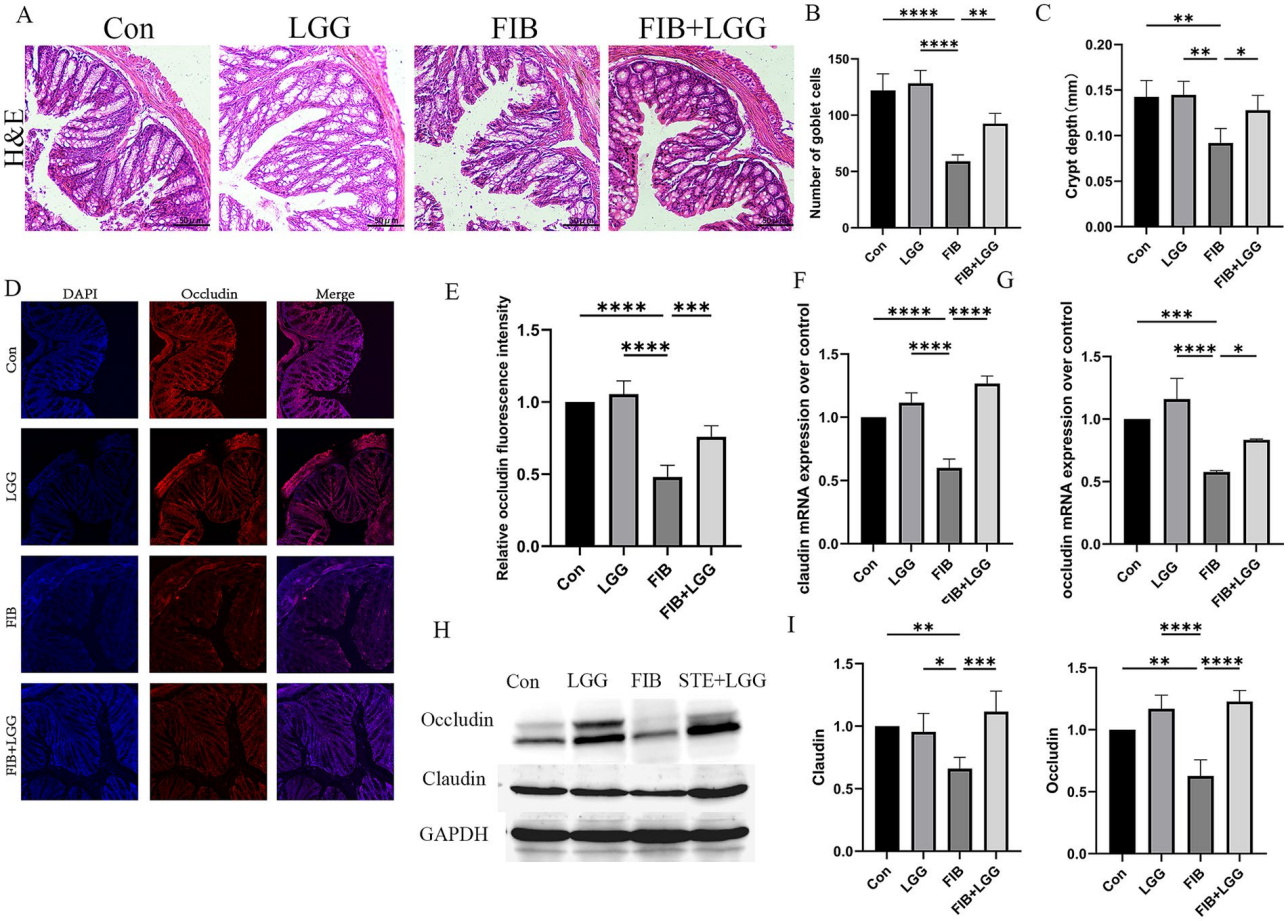
LGG improves intestinal barrier damage in HFD-induced MASLD mice. **(A)** H&E staining images of mouse colonic segments. **(B)** Count of goblet cells in mouse colons. **(C)** Crypt depth in mouse colons. **(D)** Immunofluorescence images of Occludin in mouse colons. Red fluorescence represents Occludin expression, and blue fluorescence represents DAPI-stained nuclei. **(E)** Quantitative analysis of Occludin fluorescence intensity in mouse colons. **(F,G)** mRNA levels of tight junction proteins claudin and occludin. **(H)** Western blot analysis of tight junction proteins Occludin and Claudin, with GAPDH as a control. **(I,J)** Quantification of western blot results. Each value is presented as mean ± SD (*n* = 6). *p < 0.05, **p < 0.01, ***p < 0.001, ****p < 0.0001.

In the MASLD model, we found that LGG could reverse HFD-induced intestinal barrier damage, leading us to speculate that LGG might also have a therapeutic effect on intestinal permeability in MASH mice. H&E staining was used to assess colonic damage, revealing obvious epithelial cell shedding, goblet cell reduction, and shallowing of crypt depth in the colonic tissue of the FIB group compared with the control group. Counting revealed that the number of goblet cells per field of view decreased from 128 to 59, and the average crypt depth shallowed from 0.14 mm to 0.07 mm. After LGG treatment, the number of goblet cells per field of view recovered to 92, and the average crypt depth also recovered to 0.13 mm. LGG significantly alleviated the above-mentioned intestinal pathological changes (Figures 5A–C). Immunofluorescence was used to detect Occludin protein expression, revealing that Occludin was evenly distributed in red in the colonic tissue of the control group but significantly weakened and sparsely distributed in the model group compared with the control group (Figures 5D,E). Consistent with the immunofluorescence results, QPCR and western blot results also showed significantly reduced expression of Occludin and Claudin in the STE group, leading to intestinal barrier damage (Figures 5F–J). LGG treatment enhanced the expression of Occludin and Claudin, strengthening the intestinal barrier, thereby preventing bacterial translocation and mitigating the occurrence of MASH inflammation.

### 3.6 LGG reduces the levels of profibrotic cytokines in mice induced by HFD+CCl_4_

**Figure 6:**
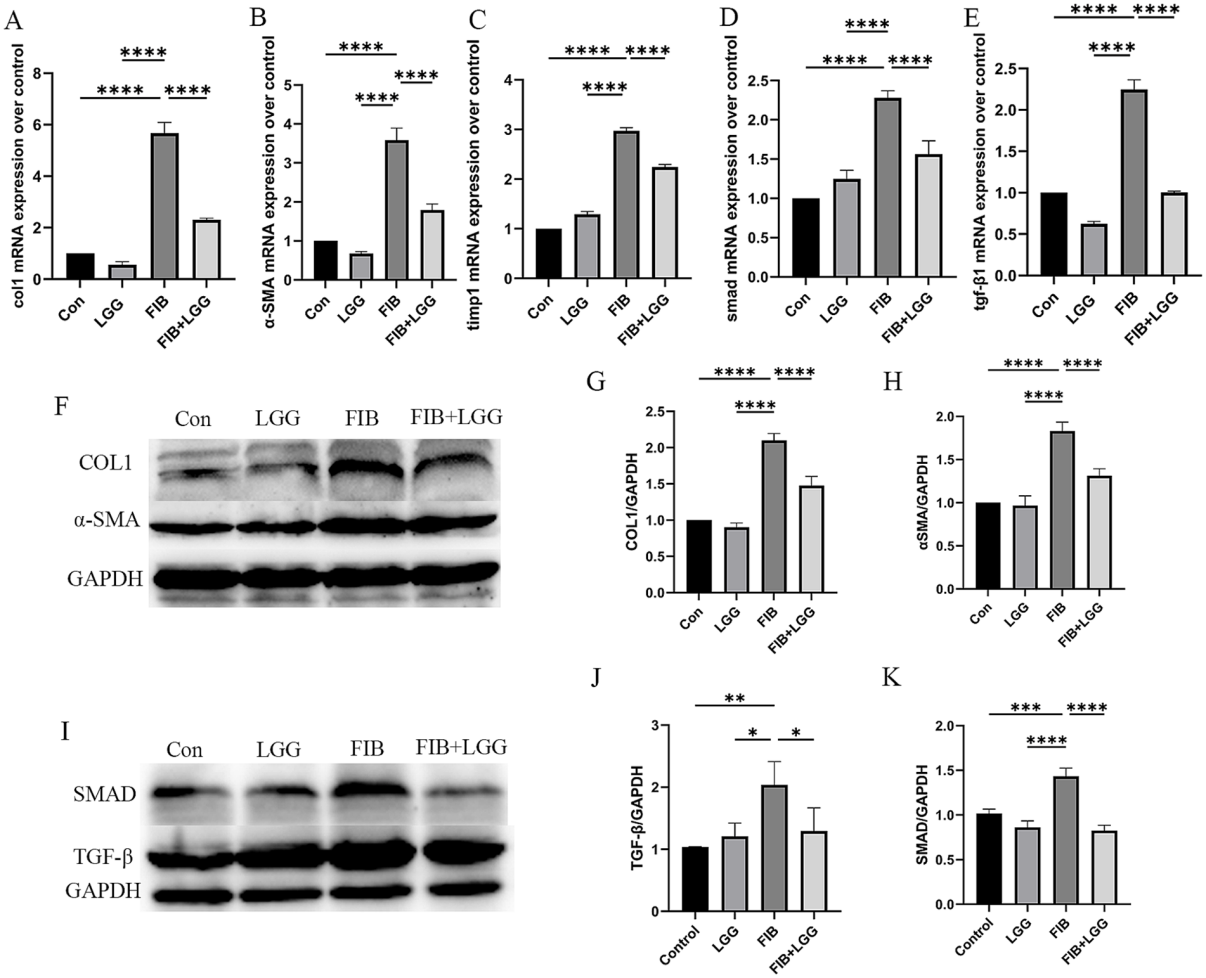
LGG reduces the levels of profibrotic cytokines in mice induced by HFD + CCl_4_. **(A–E)** q-PCR analysis of the expression of Collagen I, α-SMA, SMAD, TGF-β, and TIMP-1. **(F)** Representative western blot analysis of α-SMA and Collagen I expression. **(G-H)** Density measurements of α-SMA and Collagen I proteins normalized to GAPDH. **(I)** Representative western blot analysis of SMAD and TGF-β expression. **(J,K)** Density measurements of SMAD and TGF-β proteins normalized to GAPDH. Each value is presented as mean ± SD (*n* = 6). *p < 0.05, **p < 0.01, ***p < 0.001, ****p < 0.0001.

The TGF-β/SMAD signaling pathway is a key pathway promoting the activation of hepatic stellate cells (HSCs) and the induction of extracellular matrix (ECM) production. During liver fibrosis, the activity of this pathway is significantly enhanced, leading to HSC activation, ECM synthesis and accumulation, and liver function impairment. To further investigate the effect of LGG on liver fibrosis in mice induced by HFD + CCl_4_, we examined the mRNA levels of profibrotic markers *α*-SMA, Collagen I, tissue inhibitor of metalloproteinase-1 (TIMP-1), and TGF-β and SMAD in the TGF-β/SMAD signaling pathway. The results showed that compared with the control group, the mRNA gene expression of α-SMA, Collagen I, and TIMP-1 was significantly increased in the FIB group, while LGG treatment reduced these expressions compared with the FIB group (Figures 6A–C). Simultaneously, compared with the FIB group, LGG treatment also significantly reduced the mRNA expression of TGF-β and SMAD (Figures 6D,E). Furthermore, consistent with the QPCR results, the Western blot analysis confirmed that LGG significantly inhibited the protein transcription of α-SMA and type I collagen induced by HFD and CCl_4_ (Figure 6F), while also inhibiting the protein transcription of TGF-β and SMAD. These findings suggest that LGG can reduce the release of TGF-β, inhibit the recruitment and activation of SMAD, and thereby prevent the activation of HSCs, the synthesis and accumulation of ECM, as well as liver function impairment.

## 4 Discussion

MASH, as a progressive form of MASLD, is characterized by steatosis, lobular inflammation, and hepatocyte ballooning, with or without fibrosis (Hammerich and Tacke, 2023). Our results demonstrate that mice subjected to a 12-week high-sugar, high-fat diet with twice-weekly intraperitoneal injections of CCl_4_ during the diet period exhibited pronounced inflammation and hepatocyte ballooning, accompanied by portal-to-portal bridging fibrosis (Zhai et al., 2023). Compared with MASLD/MASH models established using HFD or CCl_4_ alone, this combined model more comprehensively mimics the pathological features of MASH, reflecting the characteristics of high-fat, high-calorie Western diets, and these symptoms are similar to the primary features observed in clinical MASLD patients.

This study aimed to investigate the efficacy of LGG in a mouse model of MASLD and MASH induced by a combination of HFD and CCl_4_. Our results show that LGG can reduce the levels of pro-inflammatory cytokines and alleviate hepatic fat accumulation, both in the MASLD and MASH stages. However, there are differences. In the MASLD model, LGG effectively regulates the composition and function of the intestinal microbiota. At the phylum level, the intestinal microbiota of MASLD patients is dominated by Firmicutes, Bacteroidetes, and Proteobacteria. Among them, Bacteroidetes can metabolize complex carbohydrates such as dietary fiber, producing SCFAs through fermentation. These SCFAs, primarily acetic acid, propionic acid, and butyric acid, are the main end products of large intestinal bacterial metabolism (Guan et al., 2022). The production and accumulation of SCFAs can stimulate the secretion of intestinal hormones such as glucagon-like peptide-1, which can affect body weight by regulating appetite and energy balance (Li et al., 2023). In addition, SCFAs serve as substrates and regulatory factors for lipid metabolism, influencing hepatic lipid metabolism and reducing the expression of adipogenic genes, thereby improving the lipid metabolism status of MASLD patients (Zhu et al., 2024). In this study, we found a decrease in the abundance of Bacteroidetes in the intestines of MASLD model mice. After LGG treatment, the abundance of Bacteroidetes increased, including three beneficial intestinal strains: *Lactobacillus reuteri*, Lachnospiraceae, and *Akkermansia muciniphila*. *Lactobacillus reuteri* can induce the production of immune cells that promote tolerance through tryptophan derivative 3-indole-acetic acid (Lai et al., 2024). Lachnospiraceae can participate in the metabolism of various carbohydrates, fermenting to produce acetic acid and butyric acid. *Akkermansia muciniphila* can repair intestinal mucosa, regulate immunity, and limit the onset of inflammation (Higarza et al., 2021). Our results demonstrate that LGG alleviates HFD-induced MASLD by reversing intestinal microbial dysbiosis and participating in the regulation of intestinal microbiota metabolism. Meanwhile, QPCR results revealed that compared with MASLD model mice, the expression of lipogenesis-related genes ACC, FAS, SREBP1, and SCD1 in the livers of mice treated with LGG was significantly reduced, suggesting that in the early stages of the disease, LGG can alleviate MASLD by regulating the composition of the intestinal microbiota, especially the abundance of Bacteroidetes, thereby promoting the production and accumulation of SCFAs (Tian et al., 2024).

In the MASH mouse model, with more liver fibrosis and severe liver inflammation, LGG treatment did not upregulate the abundance of Bacteroidetes but may have more pronounced effects in treating fibrosis and inflammation. Studies have shown that inflammatory cytokines can activate the TGF-β signaling pathway through various pathways (Wu et al., 2024). Among these inflammatory cytokines, IL-6, TNF, and TGF-β play crucial roles in maintaining immune responses, tissue repair, and homeostasis, serving as key pro-inflammatory and pro-fibrotic factors that activate hematopoietic stem cells and drive liver fibrosis (Zhang et al., 2024). For example, inflammatory cytokines such as IL-1β and TNF-*α* can upregulate the expression and activity of TGF-β, thereby activating the TGF-β/SMAD signaling pathway, promoting the proliferation and differentiation of fibroblasts (Kang et al., 2023), as well as the synthesis and secretion of extracellular matrix, thereby accelerating liver fibrosis and the progression from MASLD to MASH (Zhang et al., 2024). Our results indicate that in the MASH model, increased levels of hepatic pro-inflammatory cytokines IL-1β, IL-6, and TNF-α led to increased expression and activity of TGF-β, activating the TGF-β/SMAD signaling pathway. Western blot results showed increased protein expression levels of TGF-β, SMAD, and their downstream proteins COL1 and α-SMA in the MASH model, suggesting HSC activation and ECM production and deposition (Wei et al., 2023), which drive the progression of liver fibrosis. However, LGG treatment significantly reduced the protein expression levels of TGF-β, SMAD, COL1, and α-SMA, suggesting that when the disease progresses to the MASH stage, the role of LGG may be more pronounced in regulating the TGF-β1/SMAD signaling pathway, thereby improving liver fibrosis.

Meanwhile, numerous studies have confirmed that the disruption of the intestinal barrier plays a crucial role in the deterioration of MASLD (Song et al., 2023). Studies have shown that when the intestinal barrier is damaged, intestinal microorganisms and their metabolites (such as endotoxin LPS) may enter the liver through the portal vein, activating immune cells (such as Kupffer cells) in the liver, inducing innate immune responses, thereby initiating or exacerbating liver inflammation and releasing inflammatory cytokines (Zhou et al., 2017). Among these inflammatory cytokines, IL-6, TNF, and TGF-β are key pro-inflammatory and pro-fibrotic factors that maintain immune responses, tissue repair, and homeostasis, and activate hematopoietic stem cells and drive liver fibrosis (Zhang et al., 2023). Our results found that compared with the MASLD model, the intestinal barrier disruption was more significant in the MASH model. Tight junction proteins Claudin and Occludin maintain the apical and basolateral membrane domains between epithelial cells (Chen et al., 2023). The metabolic products of LGG, such as short-chain fatty acids (SCFAs), can regulate actin reorganization and stabilize tight junction structures. In our experiments, we found that among the two tight junction proteins, Occludin and Claudin, Claudin is more sensitive to LGG or inflammatory factors. Taking LGG alone may also lead to an overexpression of Claudin after treatment. Therefore, we speculate that during the recovery phase of the disease, when inflammatory factors subside, there might be an overexpression of Claudin. However, the specific mechanisms behind this are not yet clear and require further investigation. In both MASLD and MASH models, the expression of tight junction proteins Claudin and Occludin was significantly reduced, while LGG treatment significantly alleviated the intestinal barrier damage caused by HFD + CCl_4_. In summary, although LGG treatment has demonstrated certain potential in MASLD/MASH, there are still multiple deficiencies and challenges. Our findings indicate that in the MASLD model, changes in the gut microbiota were first observed in mice after they were given LGG. This enhanced the intestinal barrier and reduced bacterial translocation, thereby alleviating MASLD symptoms to some extent. However, due to the diverse mechanisms of MASLD, it is uncertain whether LGG can improve the disease through other pathways. Additionally, LGG did not alleviate the intestinal microecological disorder in the MASH phase of the disease. Future clinical studies and explorations are needed to optimize the types, doses, and treatment regimens of probiotics to improve their efficacy and safety in MASLD/MASH treatment. At the same time, it is also necessary to pay attention to the long-term effects of probiotic treatment and its impact on intestinal microbial ecological balance to ensure its safety and effectiveness in clinical applications.

## Data Availability

The original contributions presented in the study are publicly available. This data can be found here: [https://www.ncbi.nlm.nih.gov/bioproject/PRJNA1232663].
