# Supplementary figures and images for: Lactobacillus rhamnosus GG attenuates MASLD/MASH progression by modulating gut microbiota and metabolic pathways
Source: Front Microbiol. 2025 Jul 24;16:1586678. doi: 10.3389/fmicb.2025.1586678 (PMC12328336; doi:10.3389/fmicb.2025.1586678)

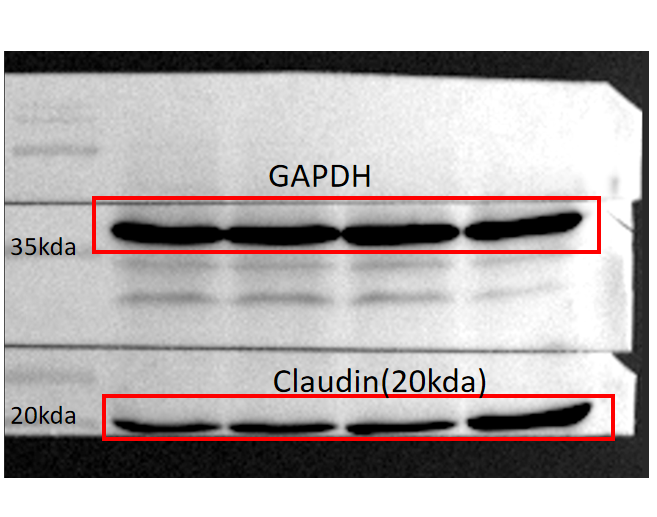

Supplement: Supplementary file 1 [file Data_Sheet_1.ZIP › Western Blot data/Figure2.H(Claudin).tif]

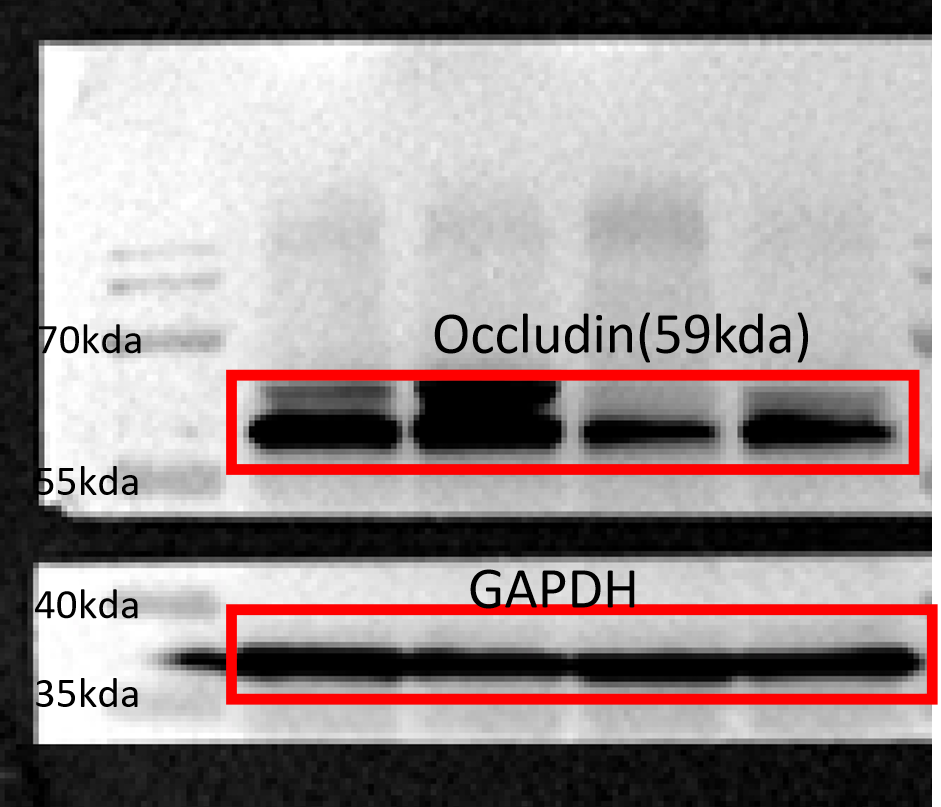

Supplement: Supplementary file 1 [file Data_Sheet_1.ZIP › Western Blot data/Figure2.H(Occludin).tif]

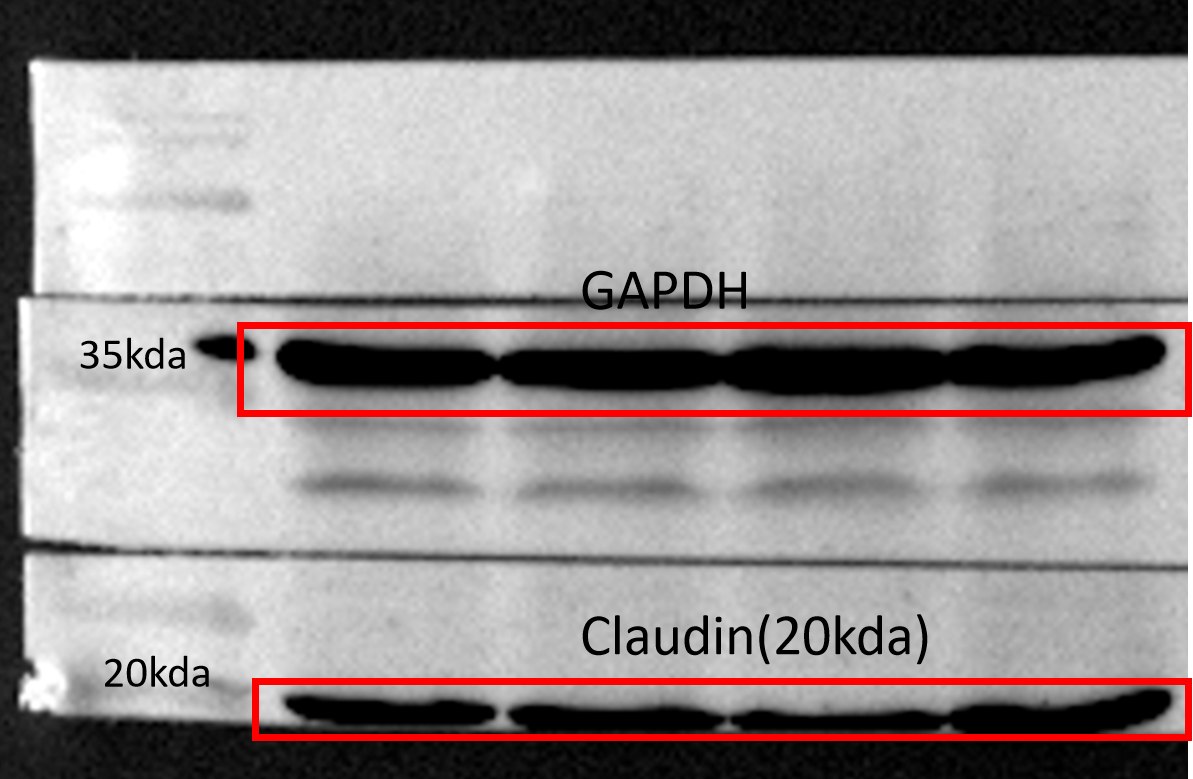

Supplement: Supplementary file 1 [file Data_Sheet_1.ZIP › Western Blot data/Figure5.H(Claudin).tif]

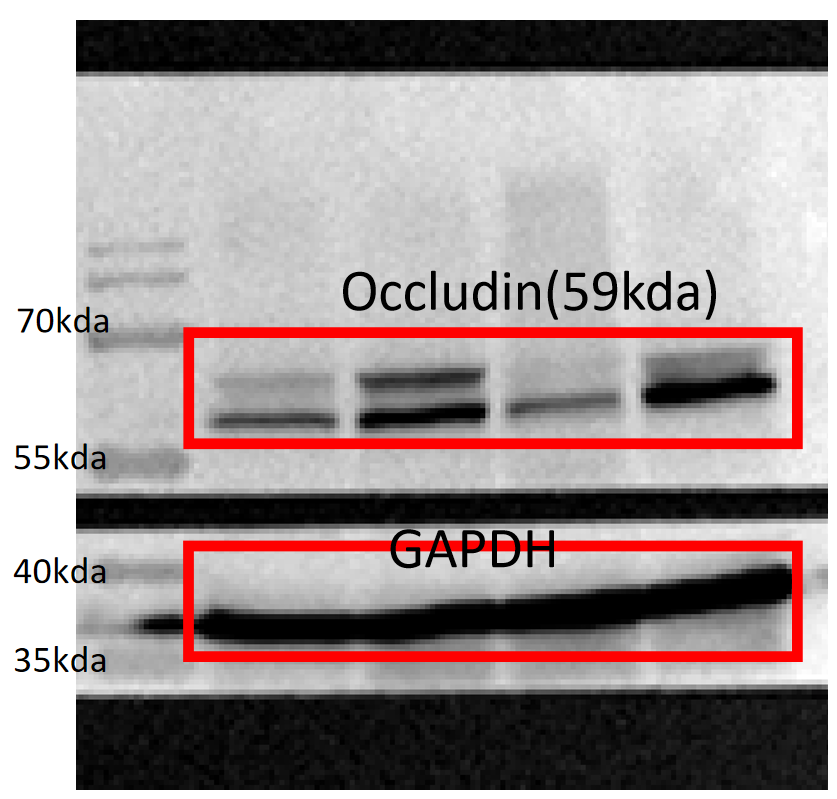

Supplement: Supplementary file 1 [file Data_Sheet_1.ZIP › Western Blot data/Figure5.H(Occludin).tif]

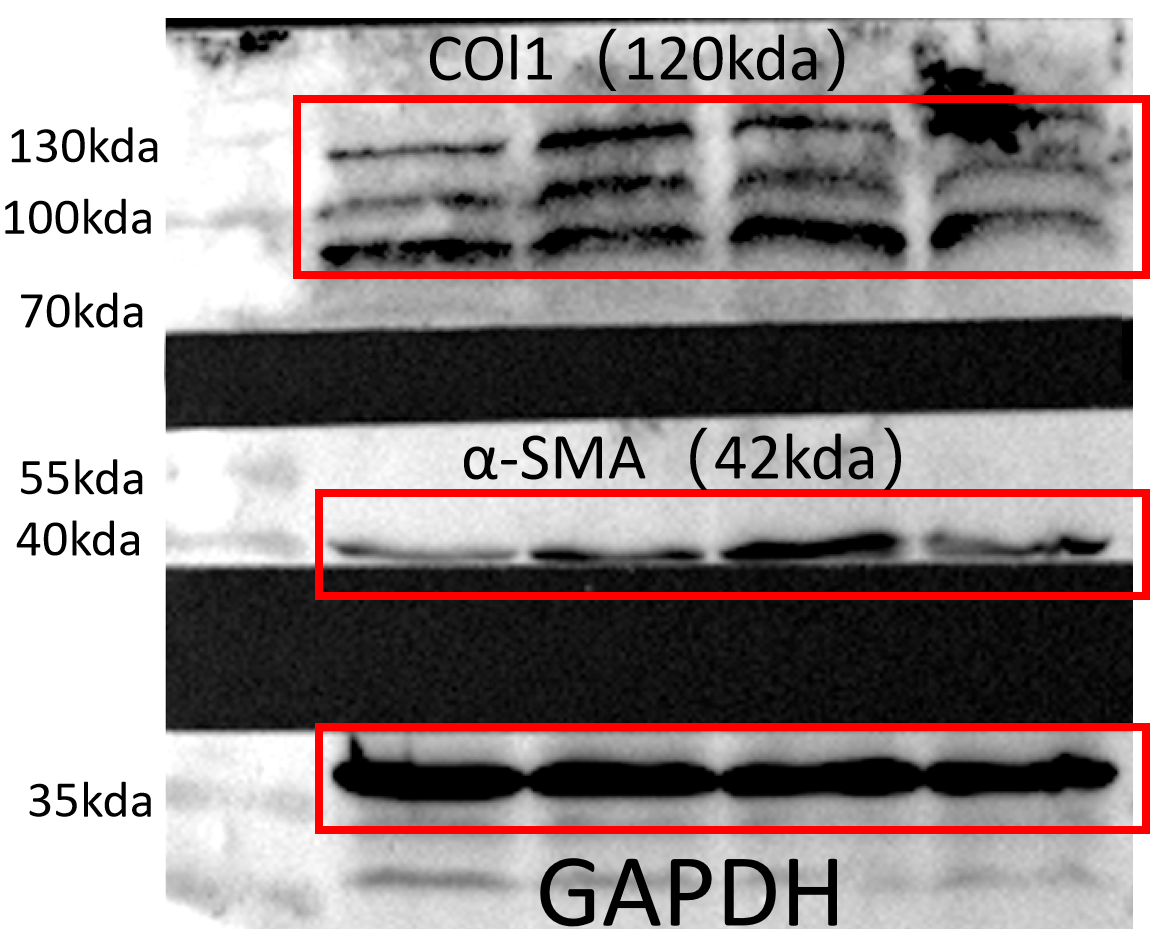

Supplement: Supplementary file 1 [file Data_Sheet_1.ZIP › Western Blot data/Figure6.F(Col1).tif]

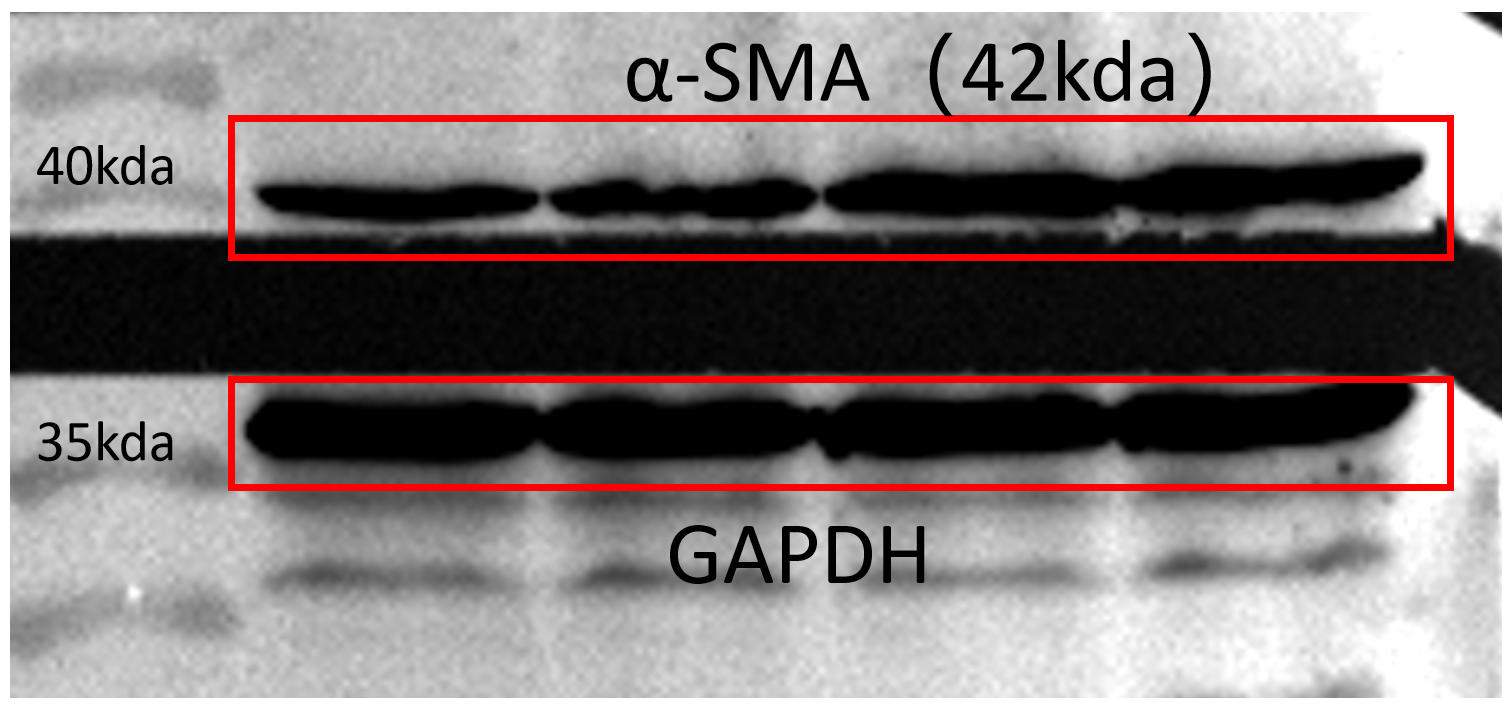

Supplement: Supplementary file 1 [file Data_Sheet_1.ZIP › Western Blot data/Figure6.F(a┴-SMA).tif]

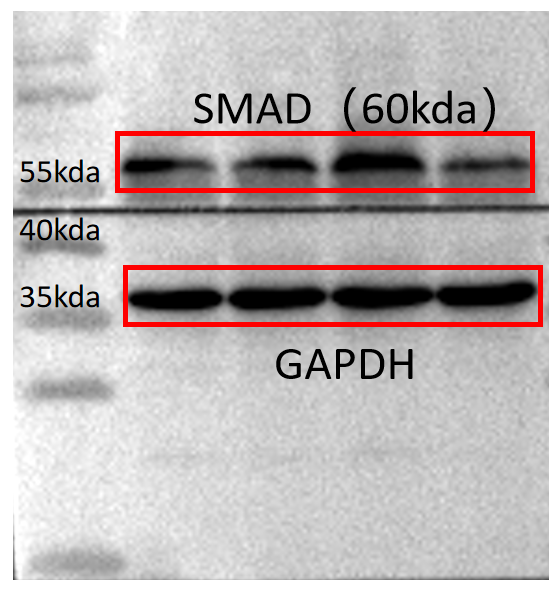

Supplement: Supplementary file 1 [file Data_Sheet_1.ZIP › Western Blot data/Figure6.I(SMAD).tif]

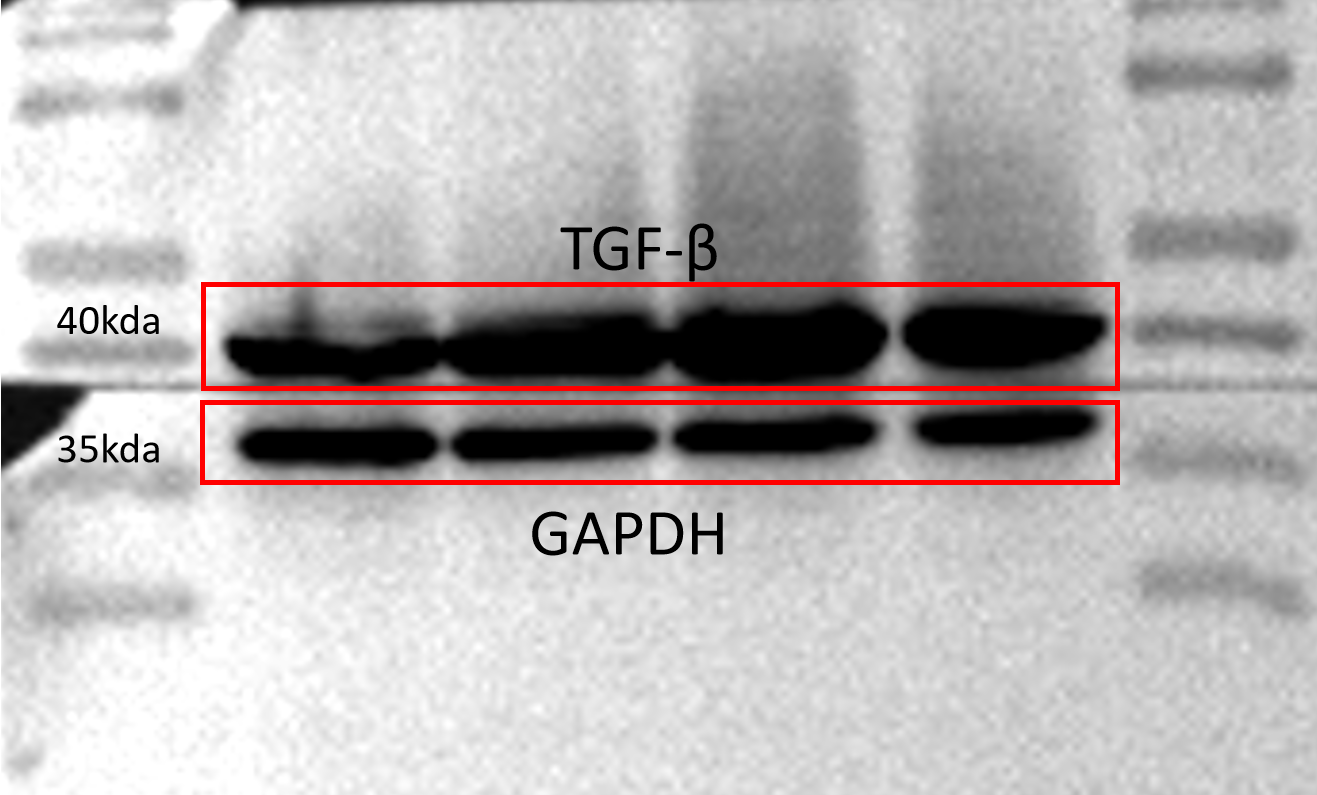

Supplement: Supplementary file 1 [file Data_Sheet_1.ZIP › Western Blot data/Figure6.I(TGF-a┬).tif]

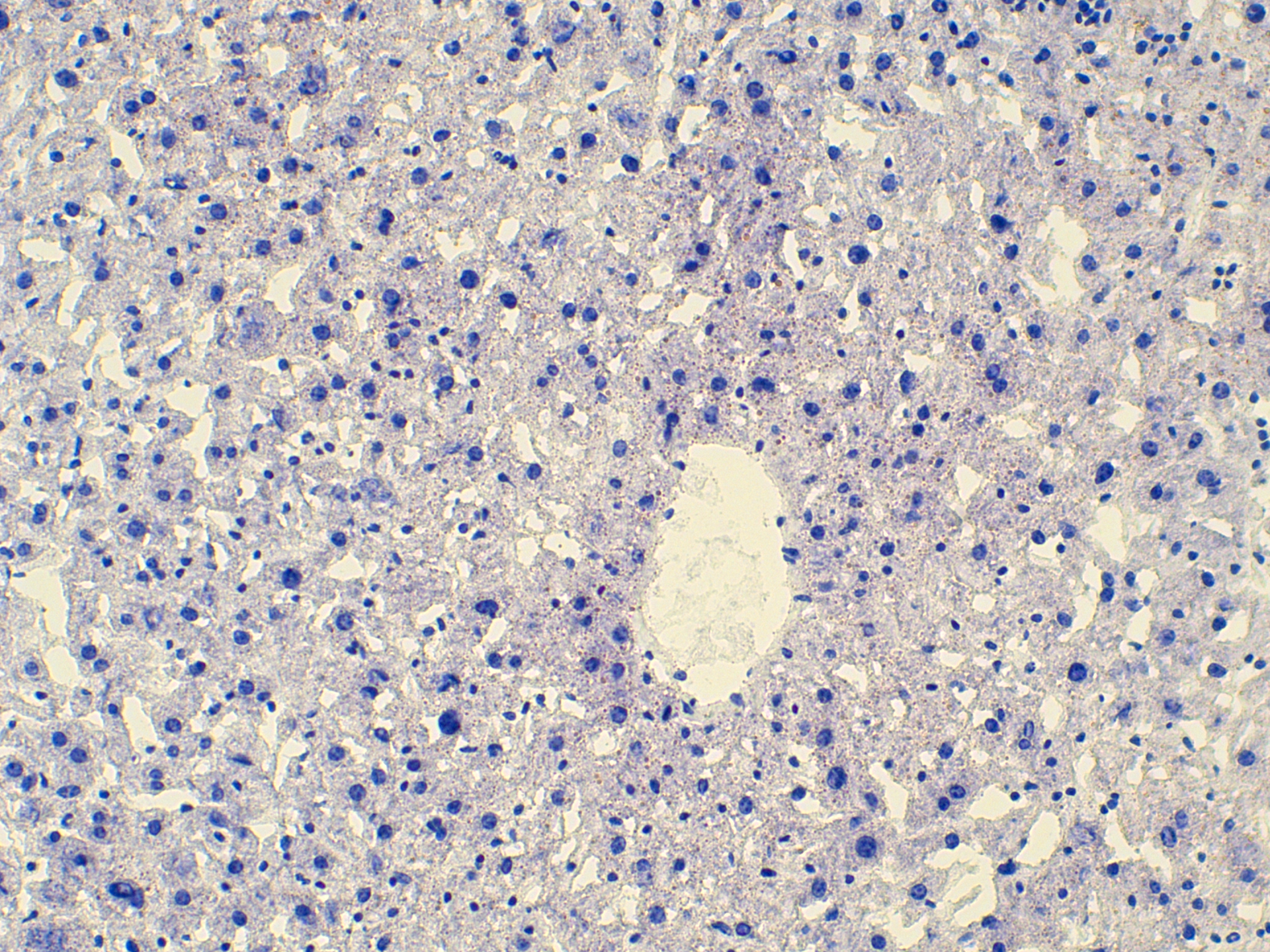

Supplement: Supplementary file 3 [file Data_Sheet_3.ZIP › Fig4.ORred/ORed-Con.tif]

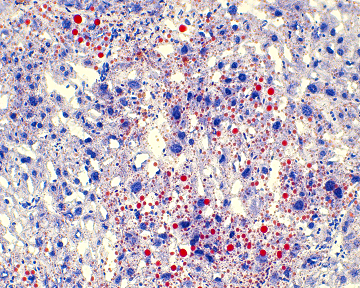

Supplement: Supplementary file 3 [file Data_Sheet_3.ZIP › Fig4.ORred/ORed-FIB.tif]

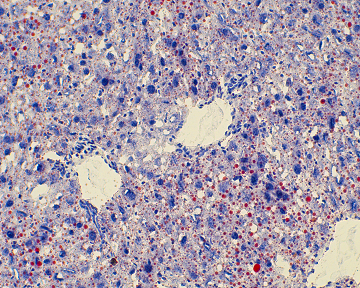

Supplement: Supplementary file 3 [file Data_Sheet_3.ZIP › Fig4.ORred/ORed-FIB+LGG.tif]

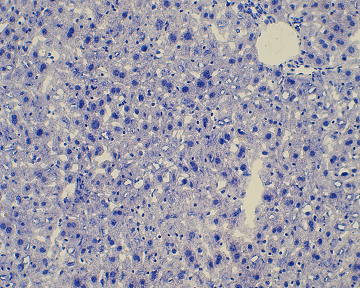

Supplement: Supplementary file 3 [file Data_Sheet_3.ZIP › Fig4.ORred/ORed-LGG.tif]

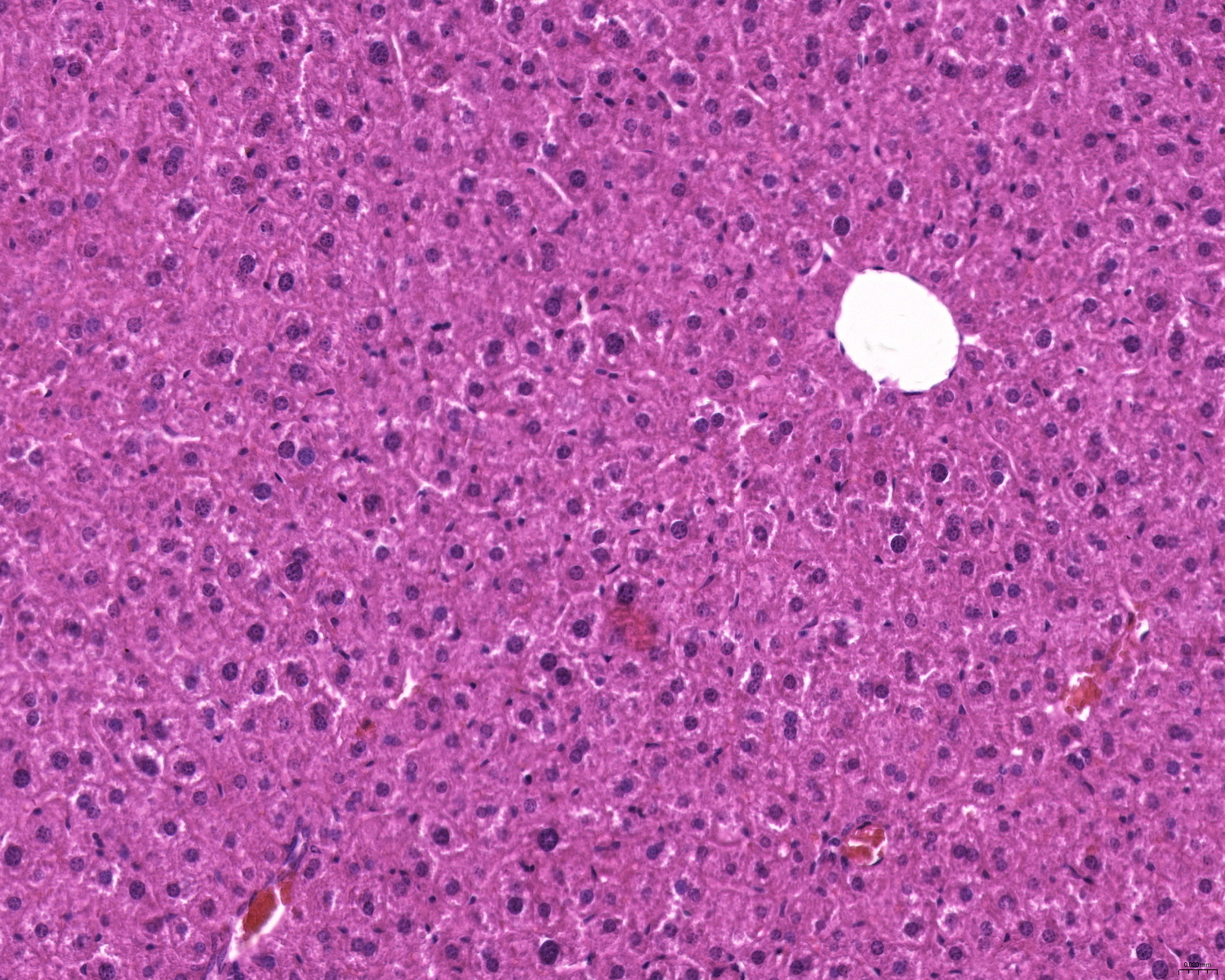

Supplement: Supplementary file 4 [file Data_Sheet_4.ZIP › Fig4.HE/HE-Con.jpg]

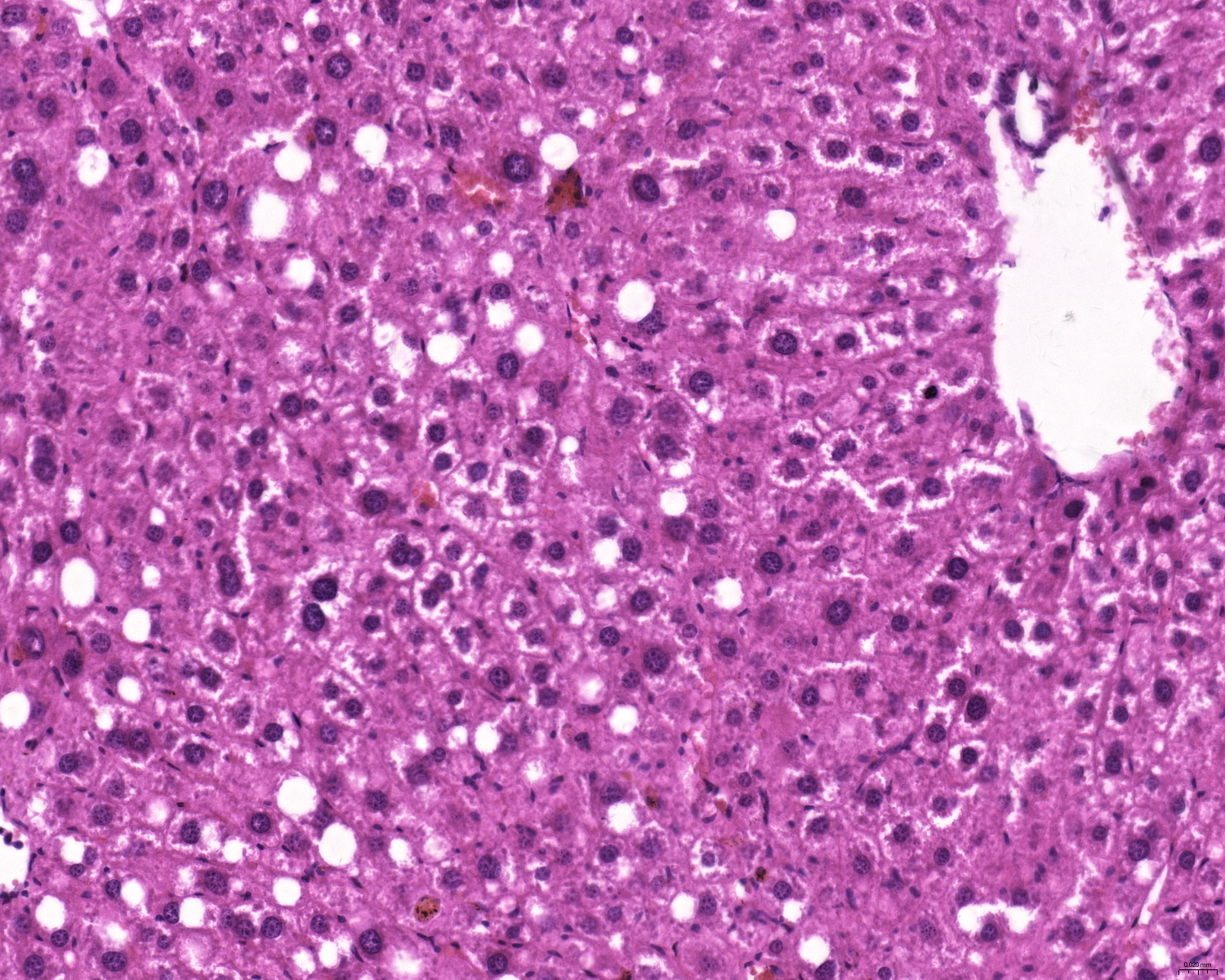

Supplement: Supplementary file 4 [file Data_Sheet_4.ZIP › Fig4.HE/HE-FIB.jpg]

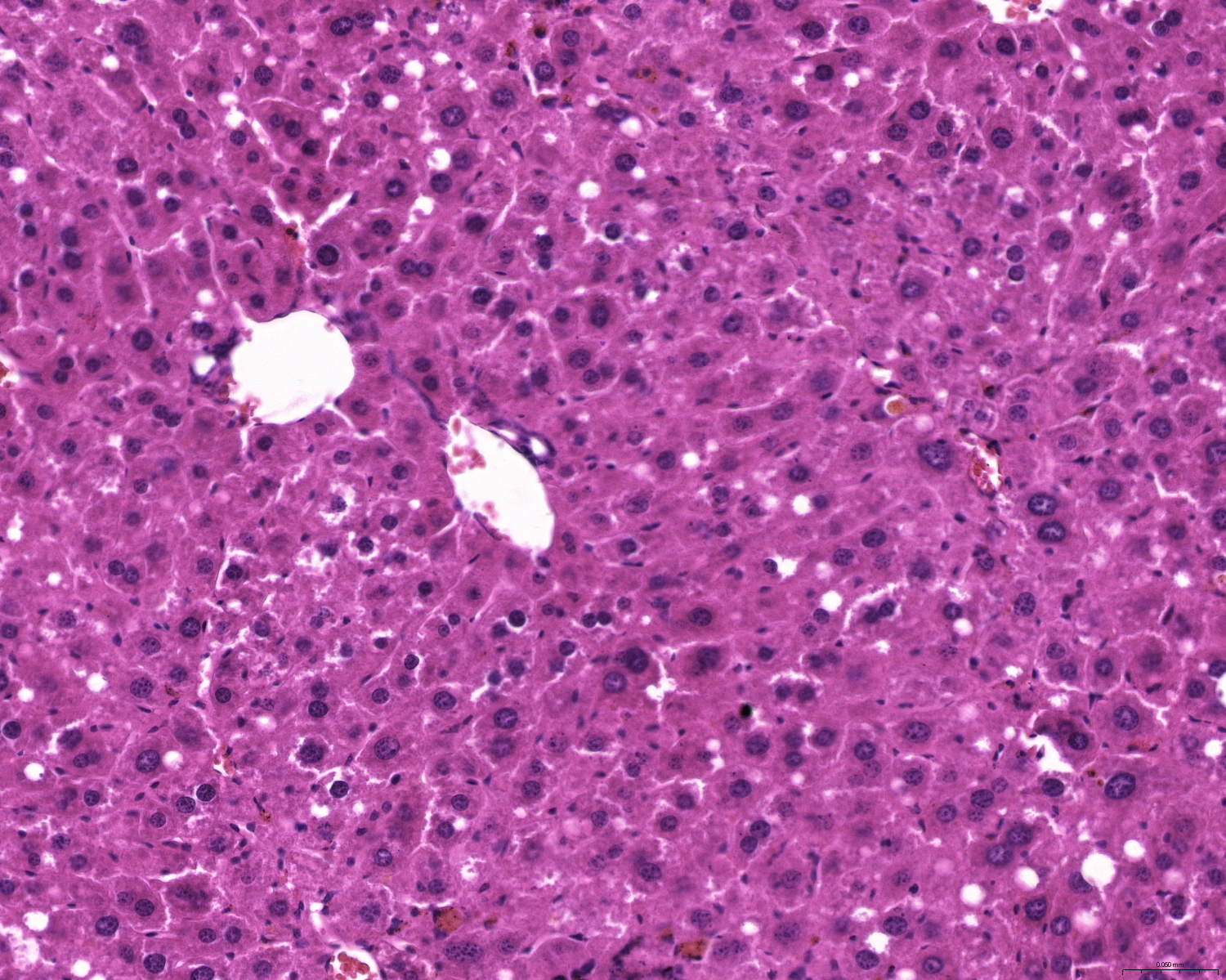

Supplement: Supplementary file 4 [file Data_Sheet_4.ZIP › Fig4.HE/HE-FIB+LGG.jpg]

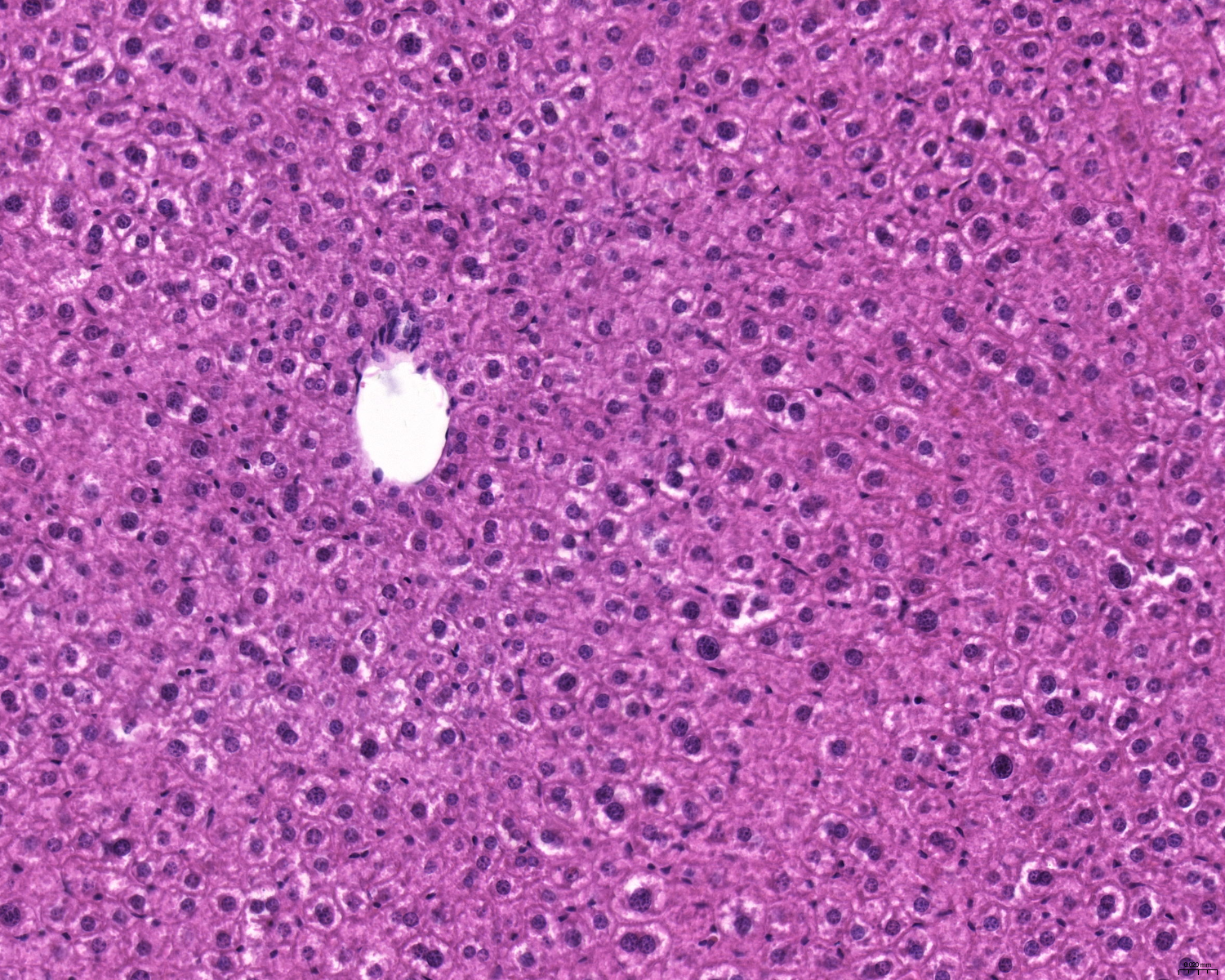

Supplement: Supplementary file 4 [file Data_Sheet_4.ZIP › Fig4.HE/HE-LGG.tif]

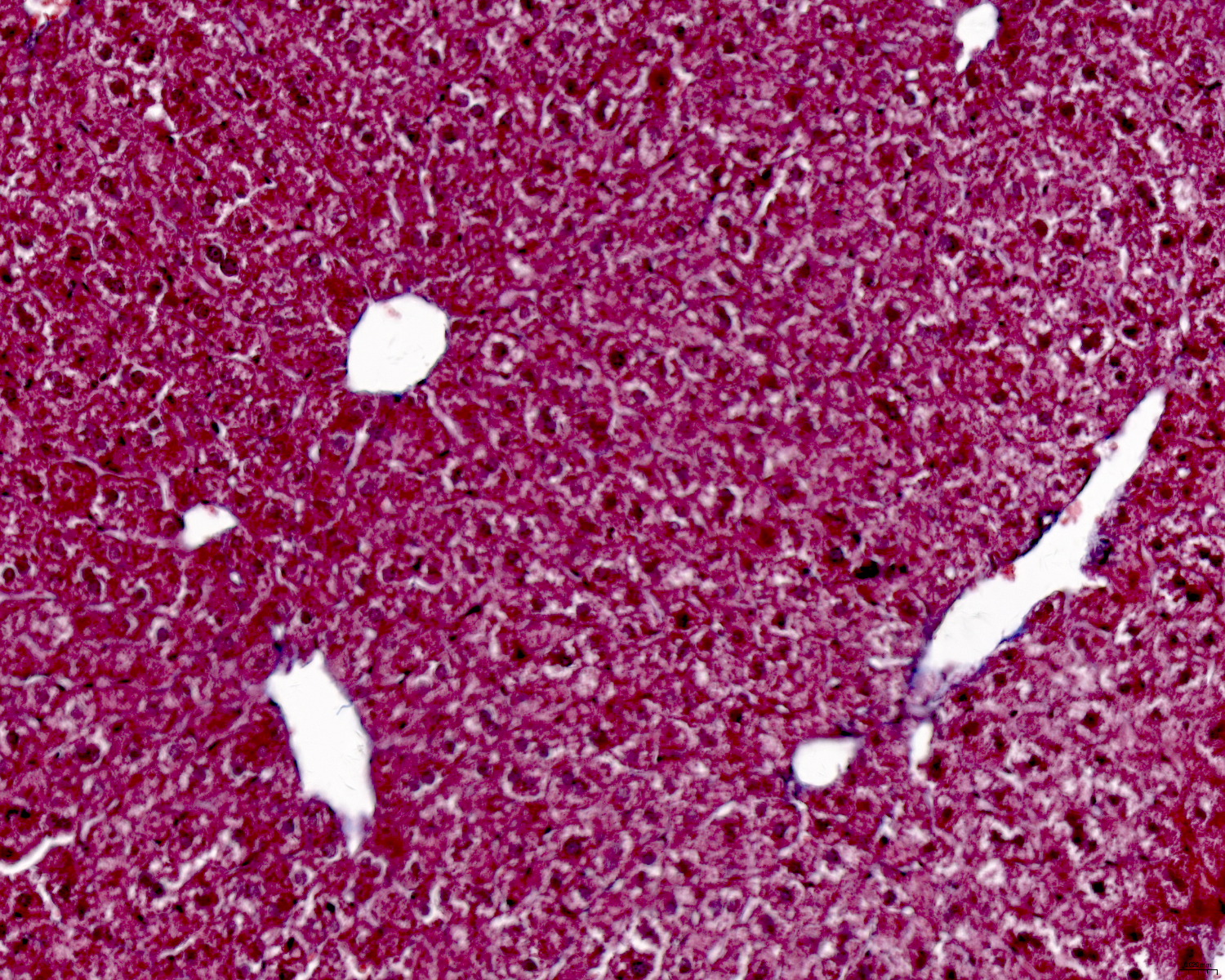

Supplement: Supplementary file 5 [file Data_Sheet_5.ZIP › Fig1.Masson/Masson-Con.tif]

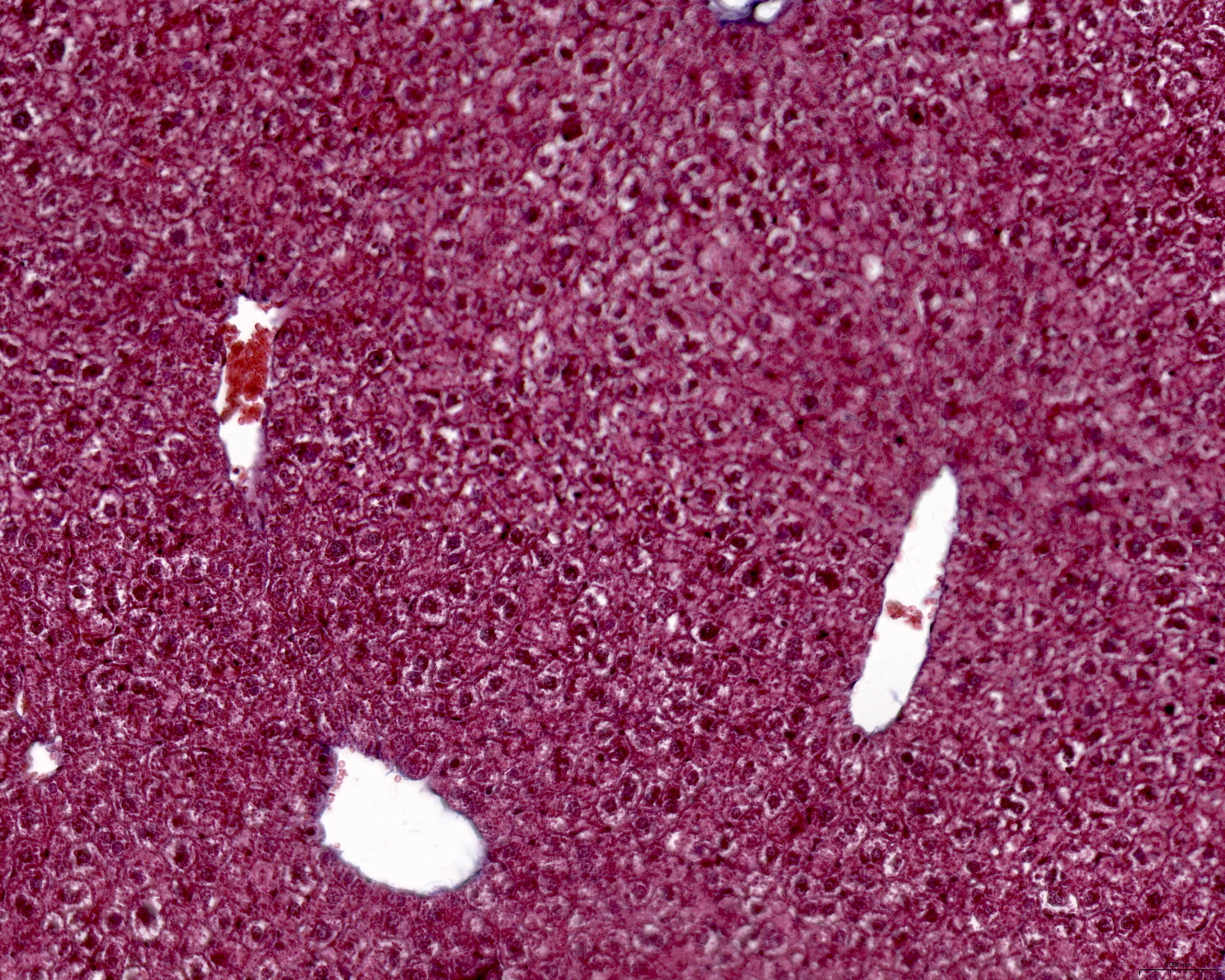

Supplement: Supplementary file 5 [file Data_Sheet_5.ZIP › Fig1.Masson/Masson-LGG.jpg]

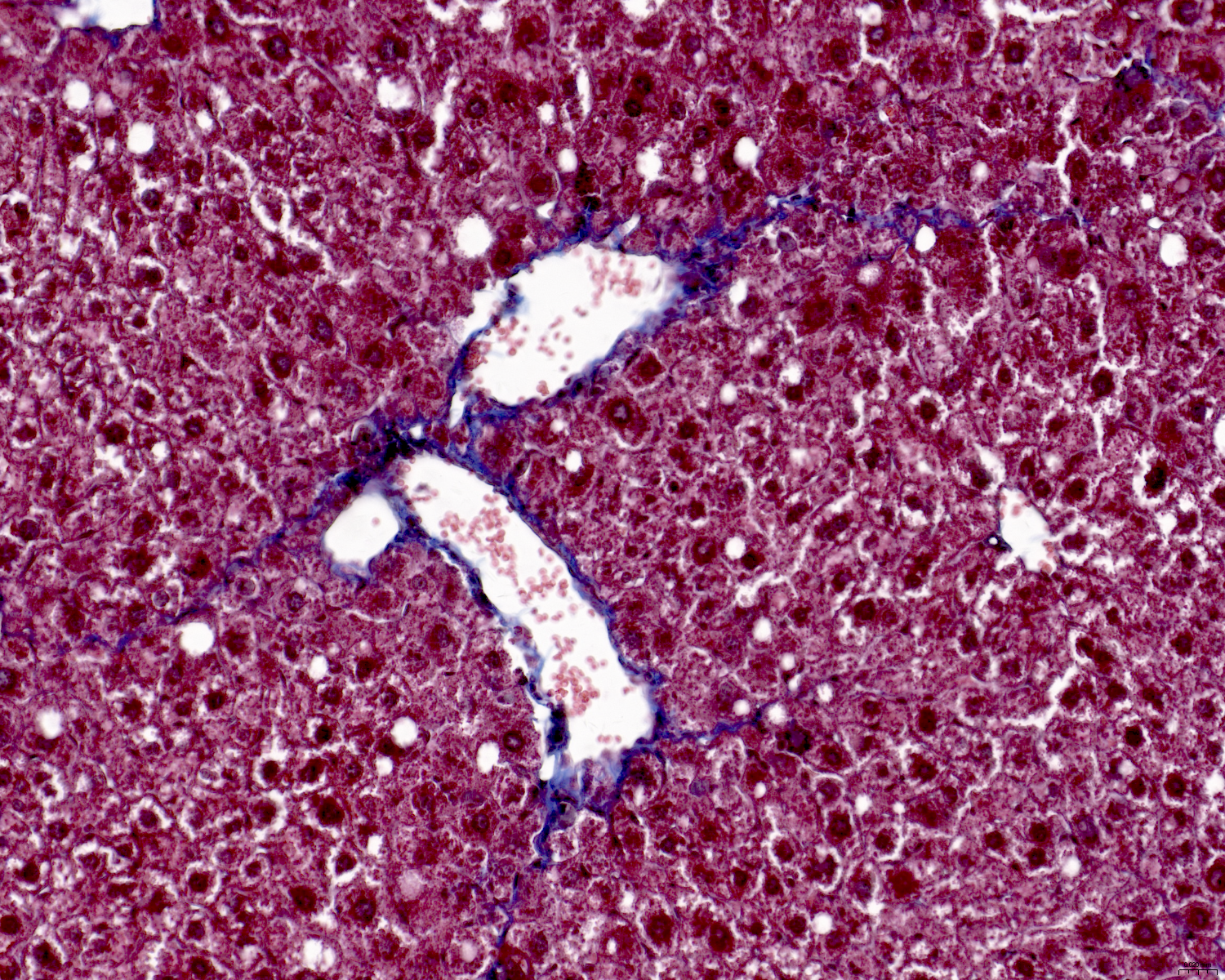

Supplement: Supplementary file 5 [file Data_Sheet_5.ZIP › Fig1.Masson/Masson-STE.tif]

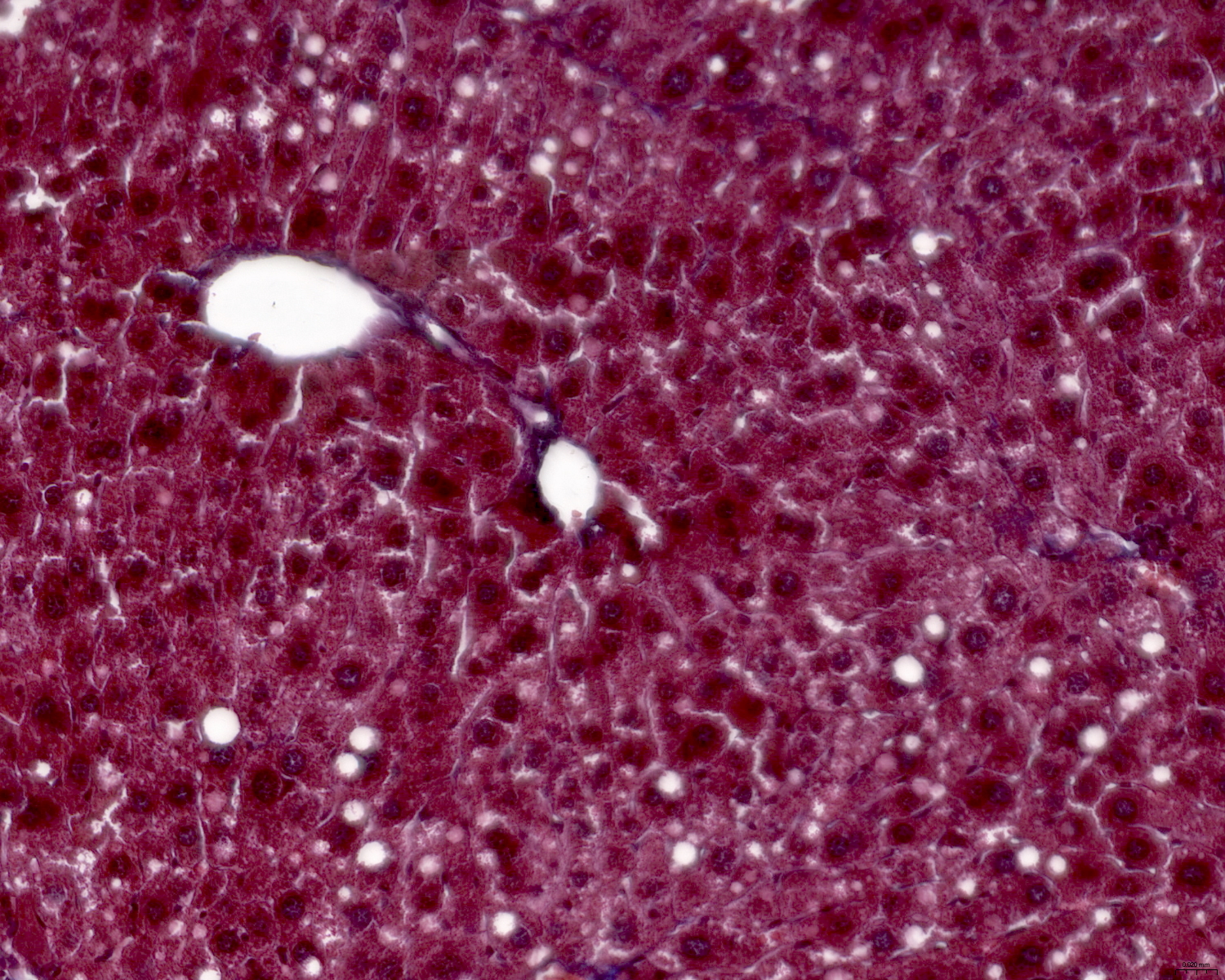

Supplement: Supplementary file 5 [file Data_Sheet_5.ZIP › Fig1.Masson/Masson-STE+LGG.jpg]

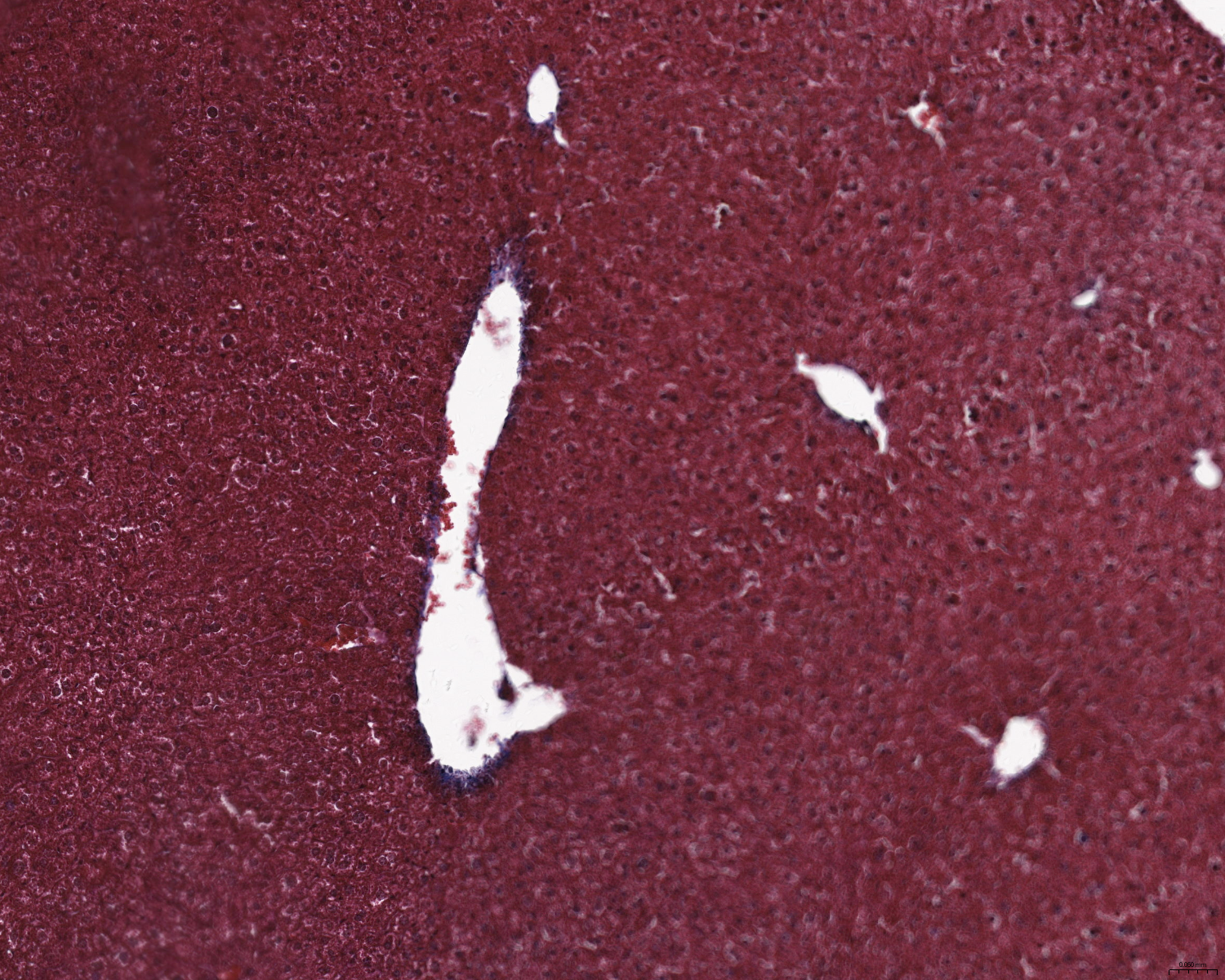

Supplement: Supplementary file 6 [file Data_Sheet_6.ZIP › Fig4.Masso/Masson-Con.tif]

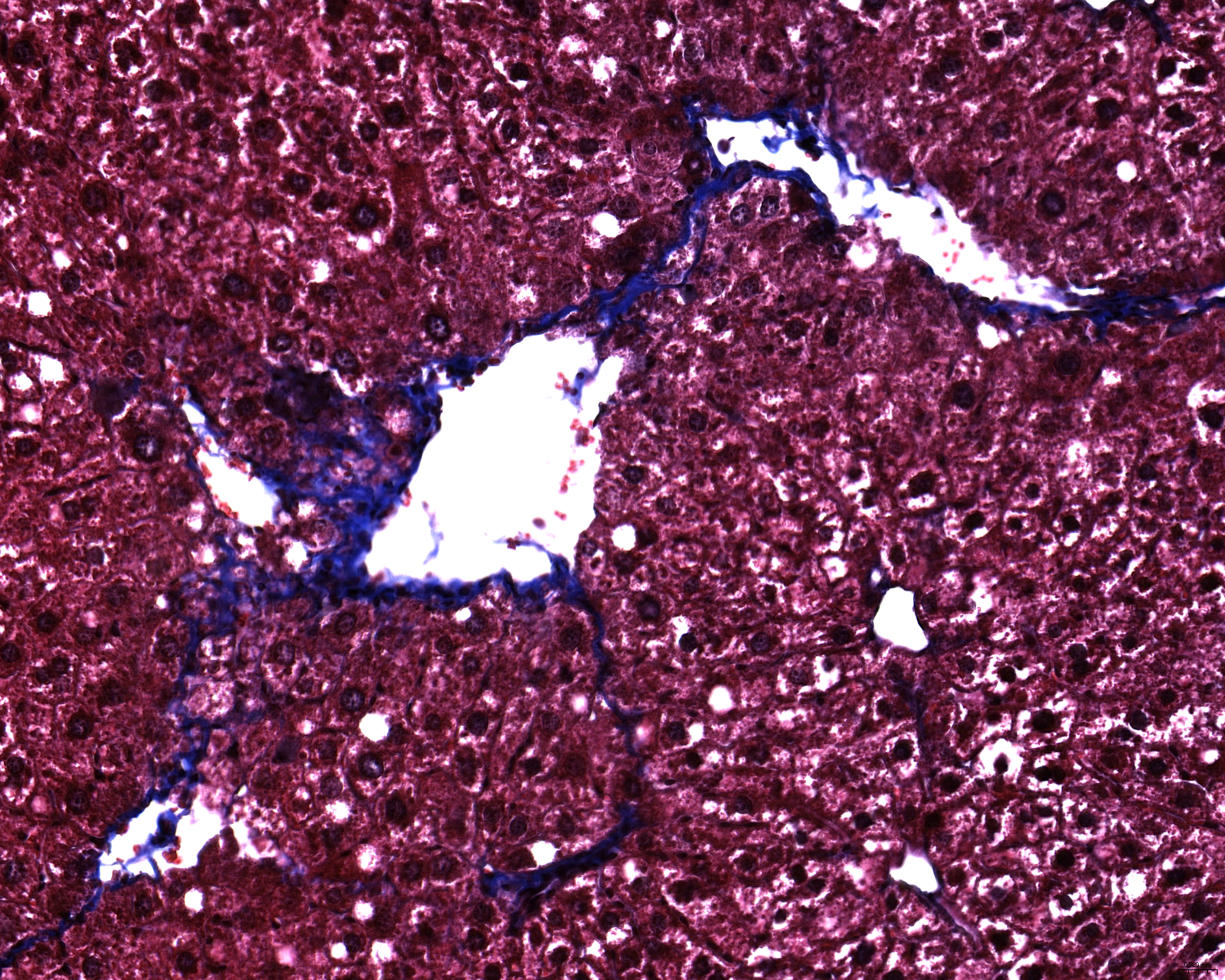

Supplement: Supplementary file 6 [file Data_Sheet_6.ZIP › Fig4.Masso/Masson-FIB.tif]

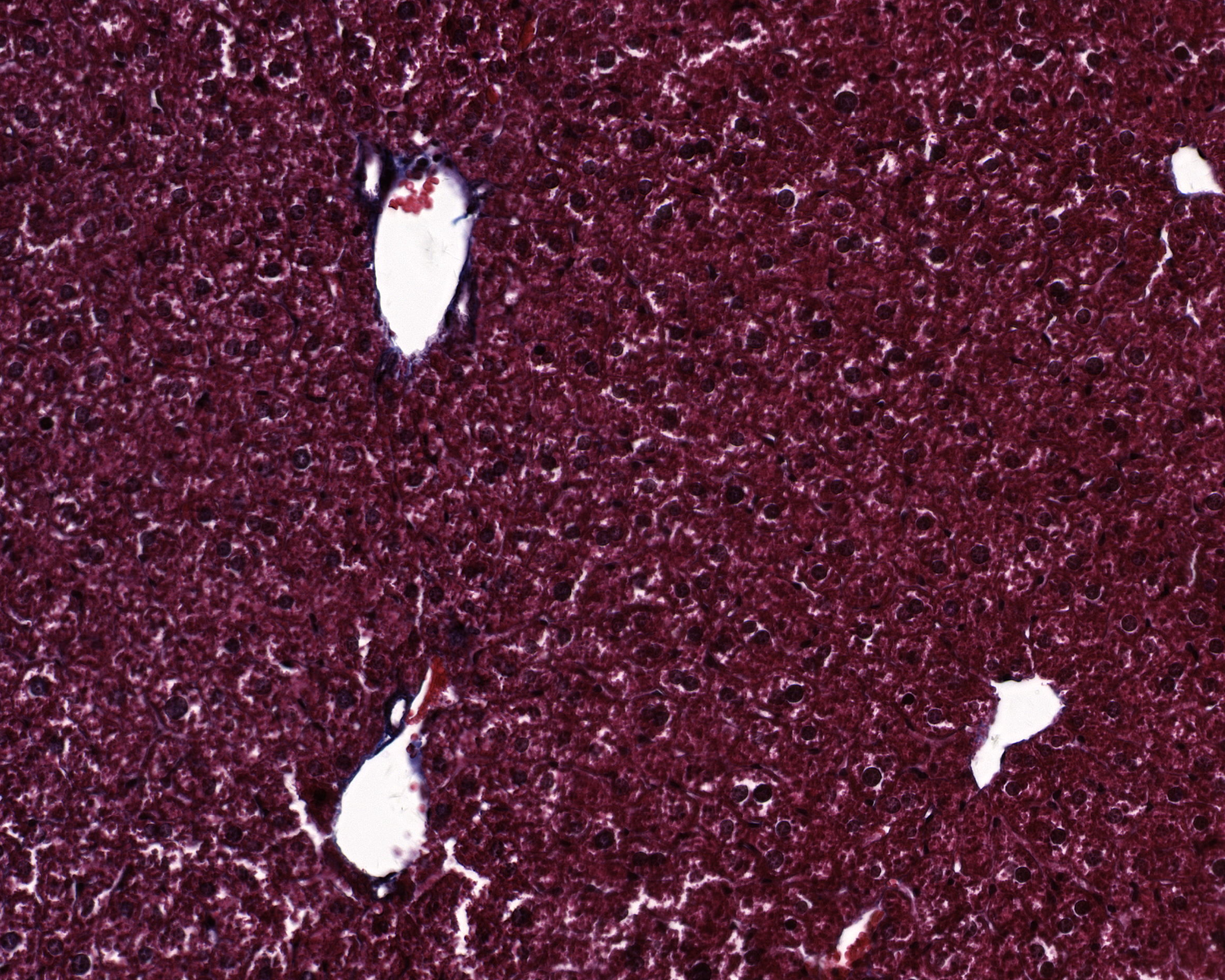

Supplement: Supplementary file 6 [file Data_Sheet_6.ZIP › Fig4.Masso/Masson-LGG.tif]

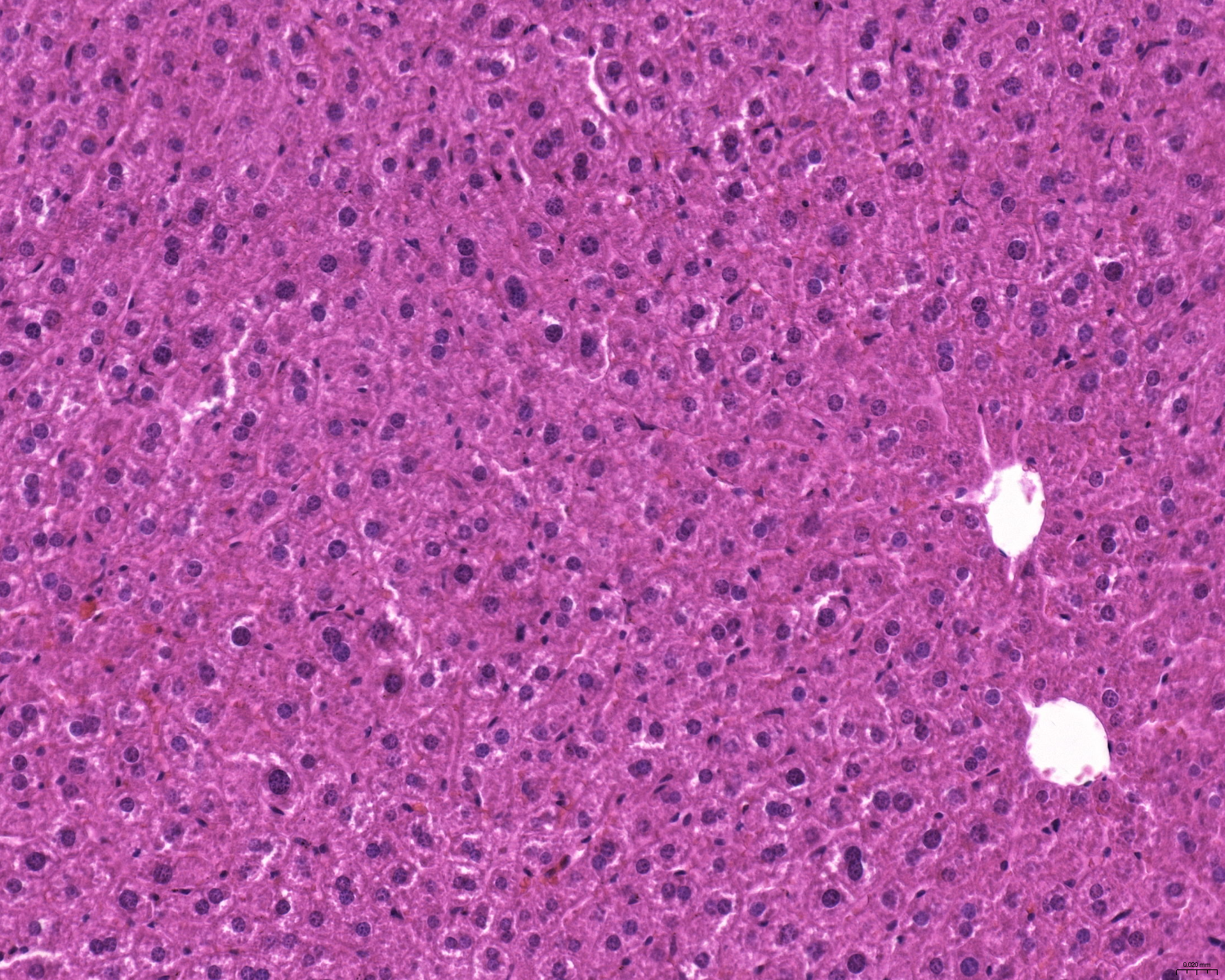

Supplement: Supplementary file 7 [file Data_Sheet_7.ZIP › Fig1.HE/HE-Con.tif]

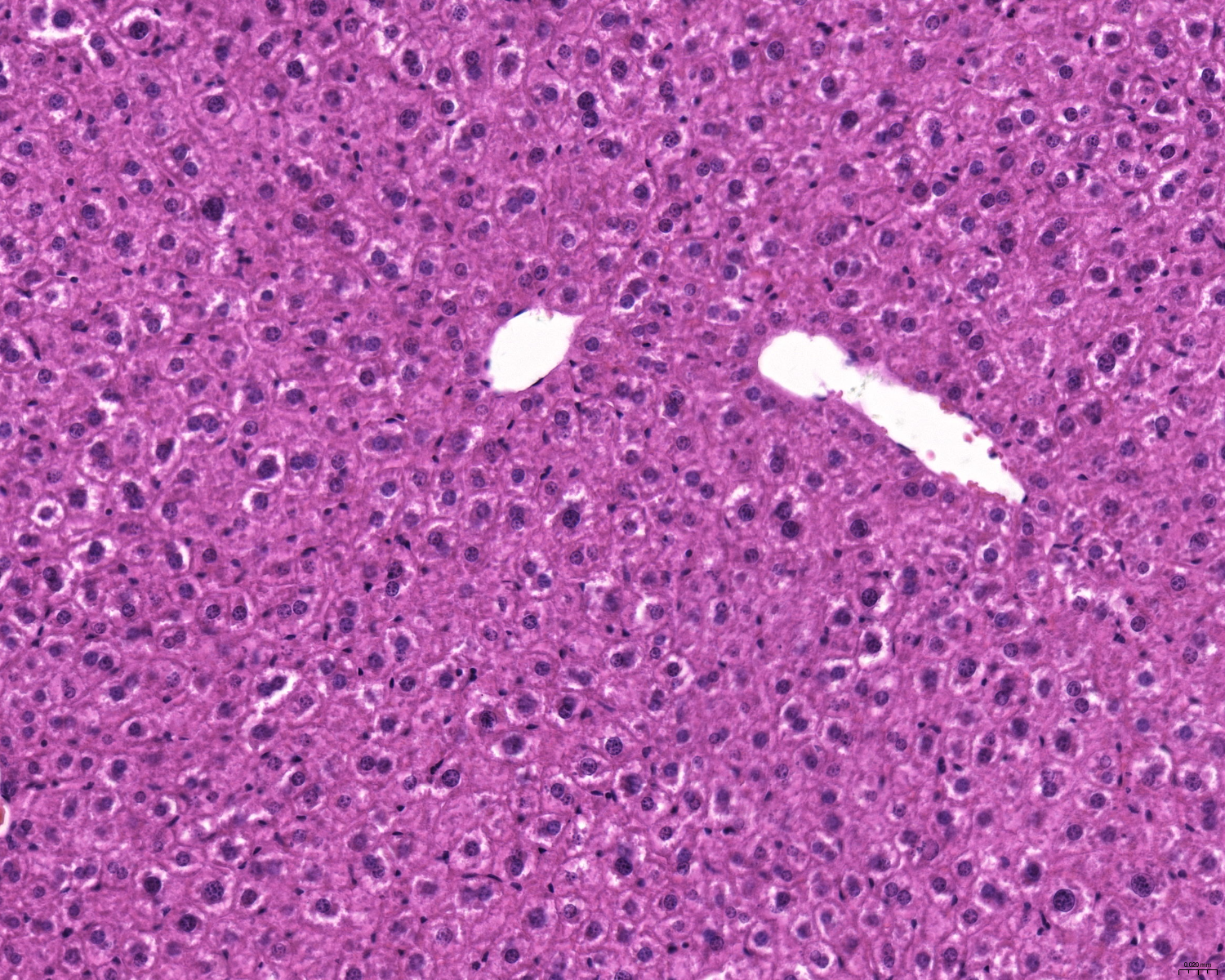

Supplement: Supplementary file 7 [file Data_Sheet_7.ZIP › Fig1.HE/HE-LGG.tif]

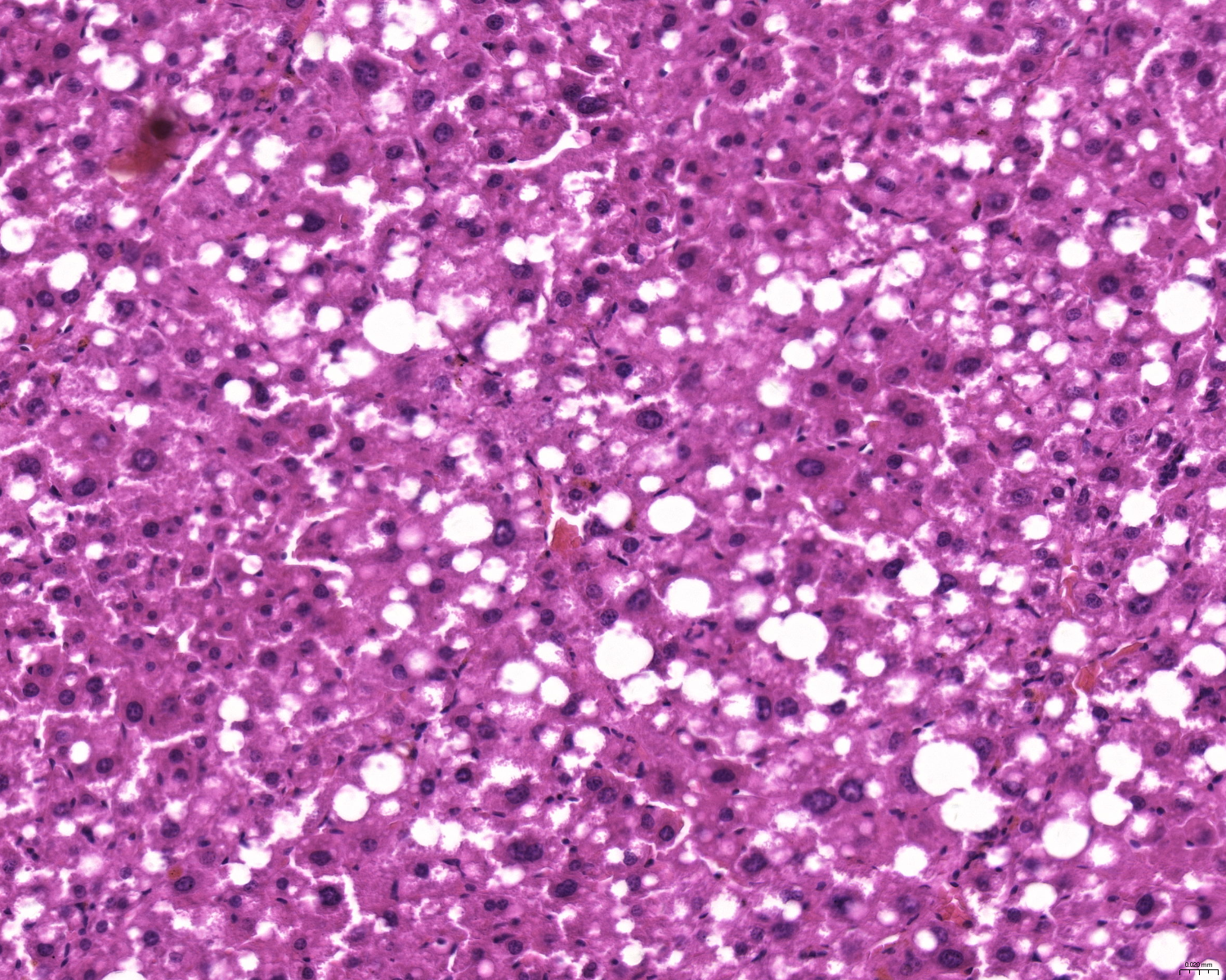

Supplement: Supplementary file 7 [file Data_Sheet_7.ZIP › Fig1.HE/HE-STE.tif]

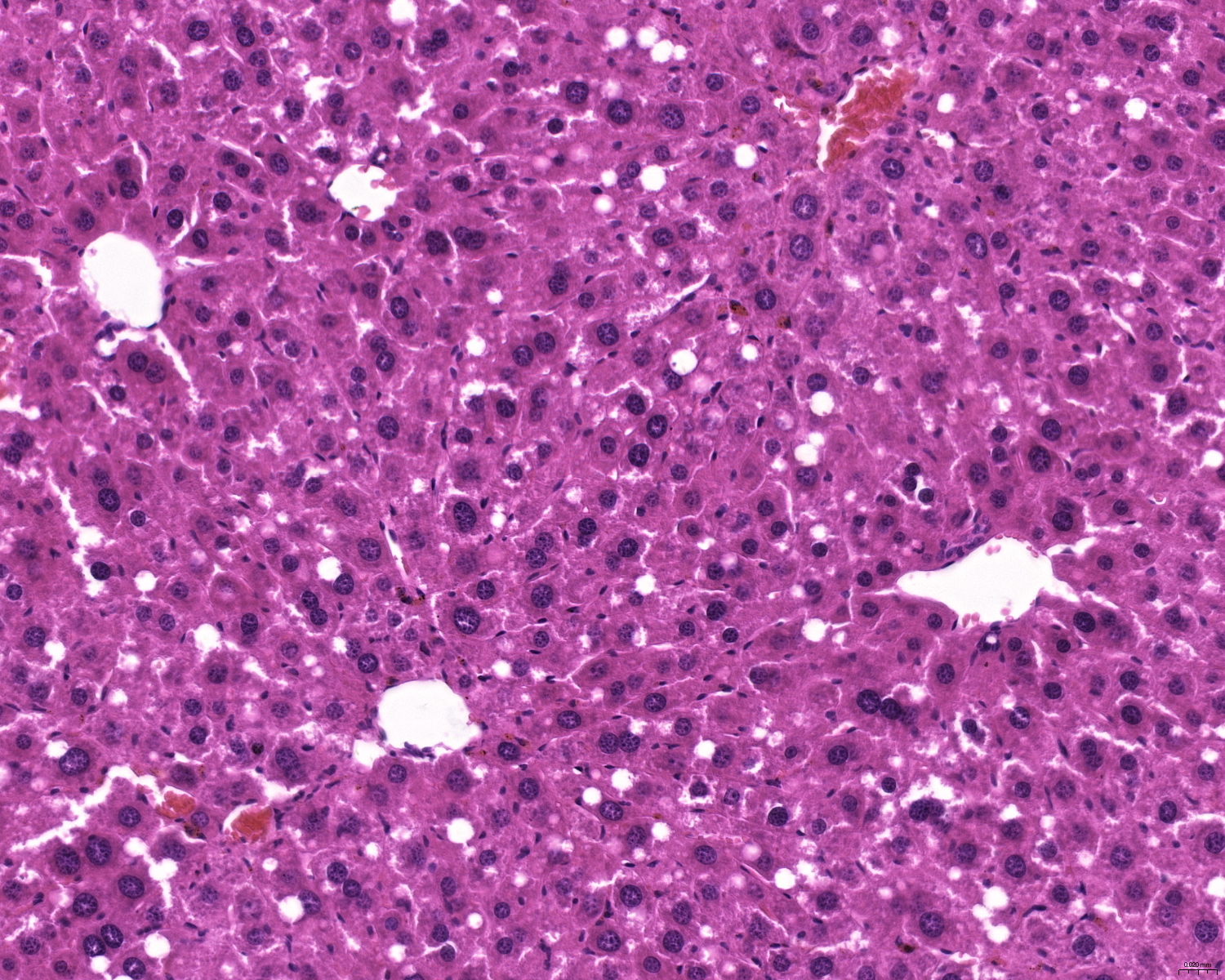

Supplement: Supplementary file 7 [file Data_Sheet_7.ZIP › Fig1.HE/HE-STE+LGG.jpg]

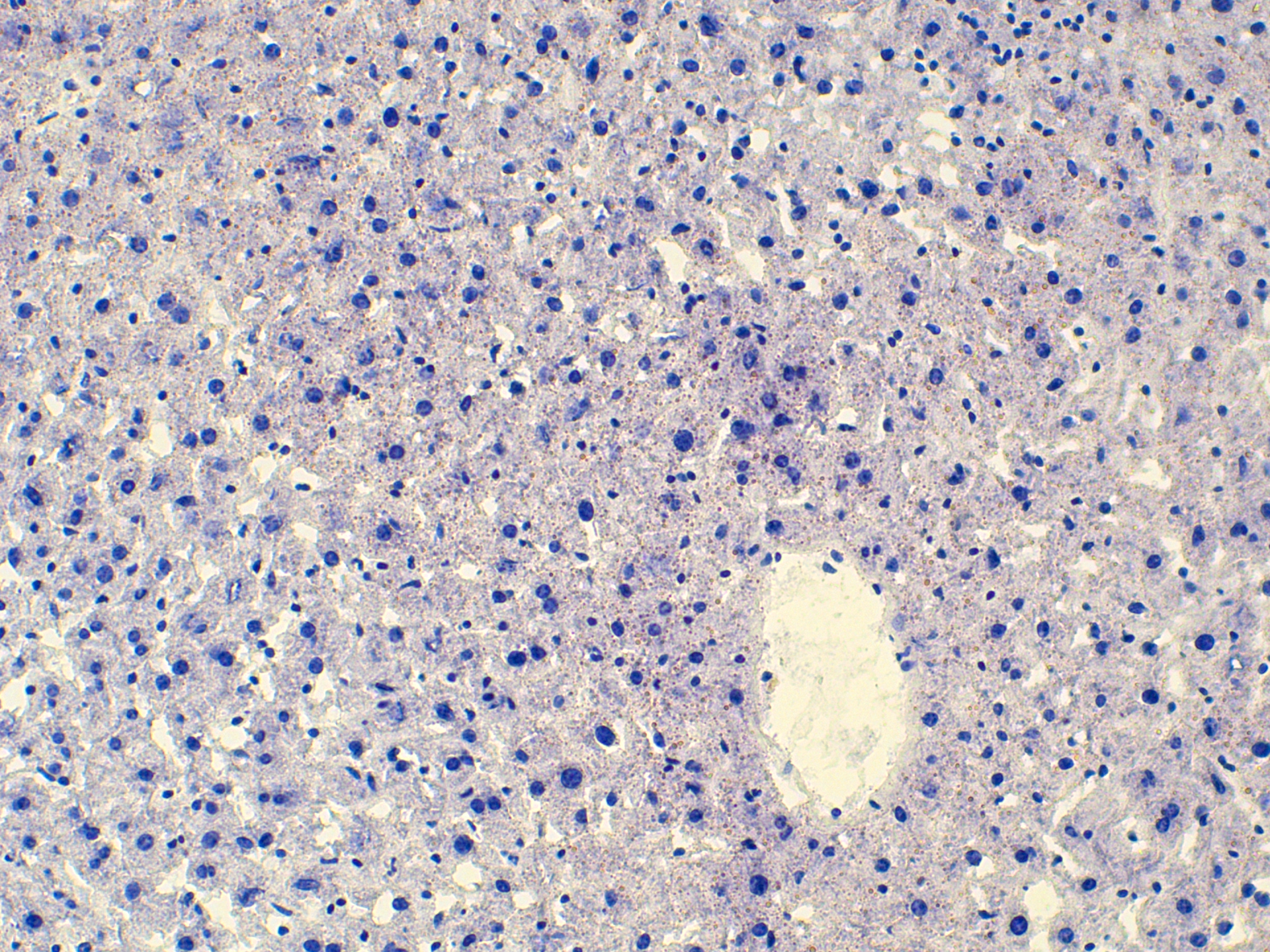

Supplement: Supplementary file 8 [file Data_Sheet_8.ZIP › Fig1.ORred/ORed-Con.tif]

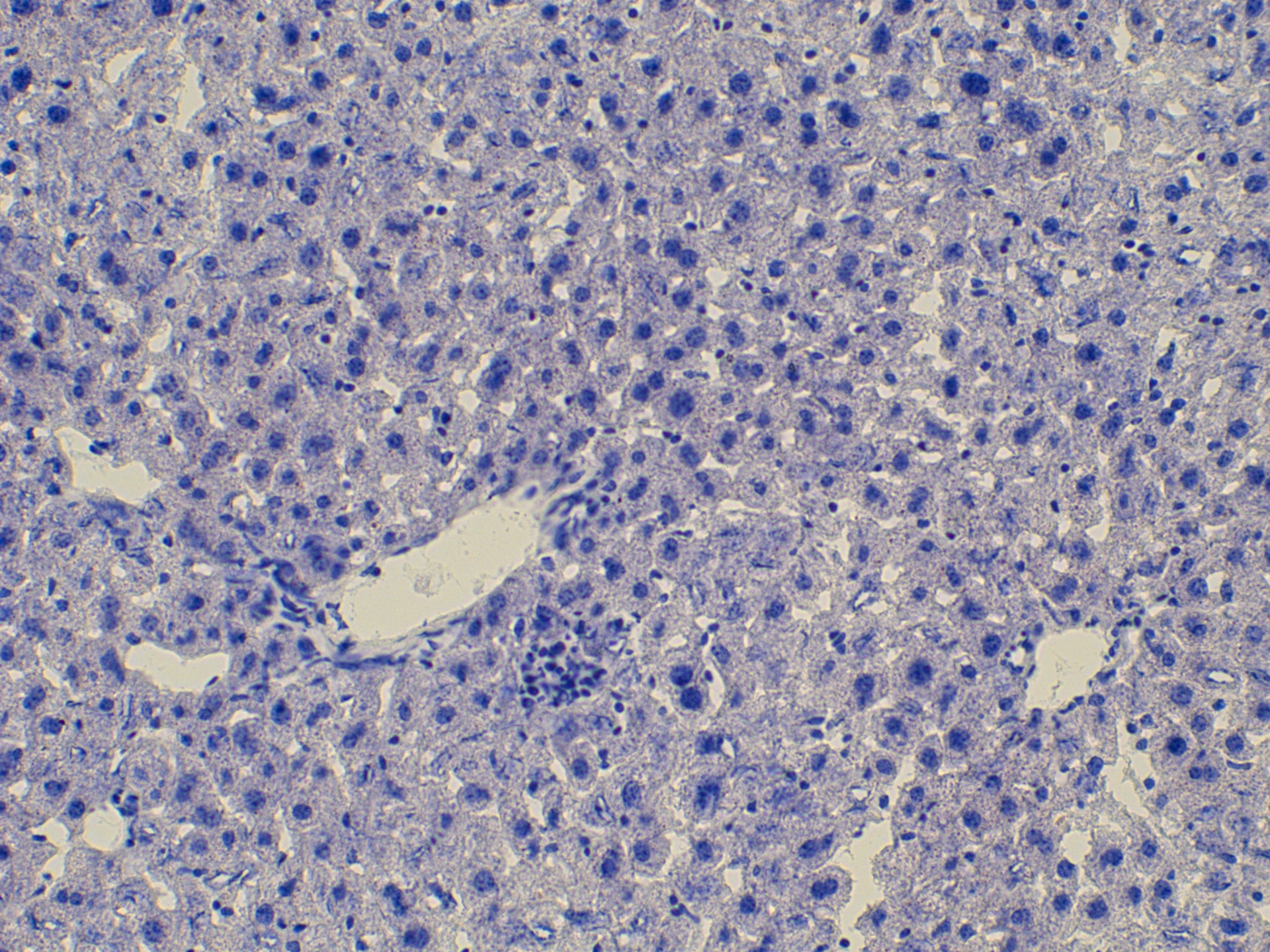

Supplement: Supplementary file 8 [file Data_Sheet_8.ZIP › Fig1.ORred/ORed-LGG.tif]

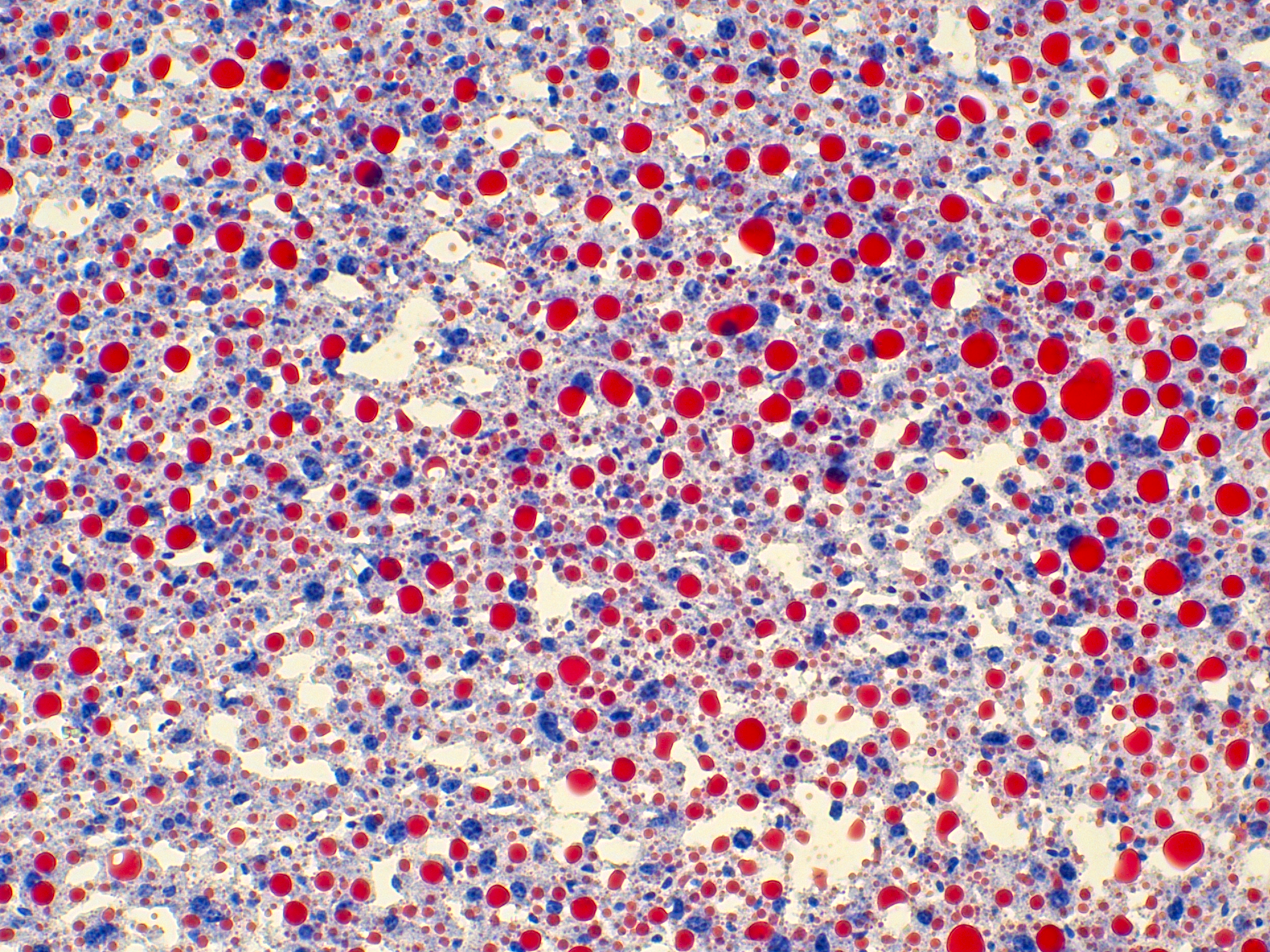

Supplement: Supplementary file 8 [file Data_Sheet_8.ZIP › Fig1.ORred/ORed-STE.tif]

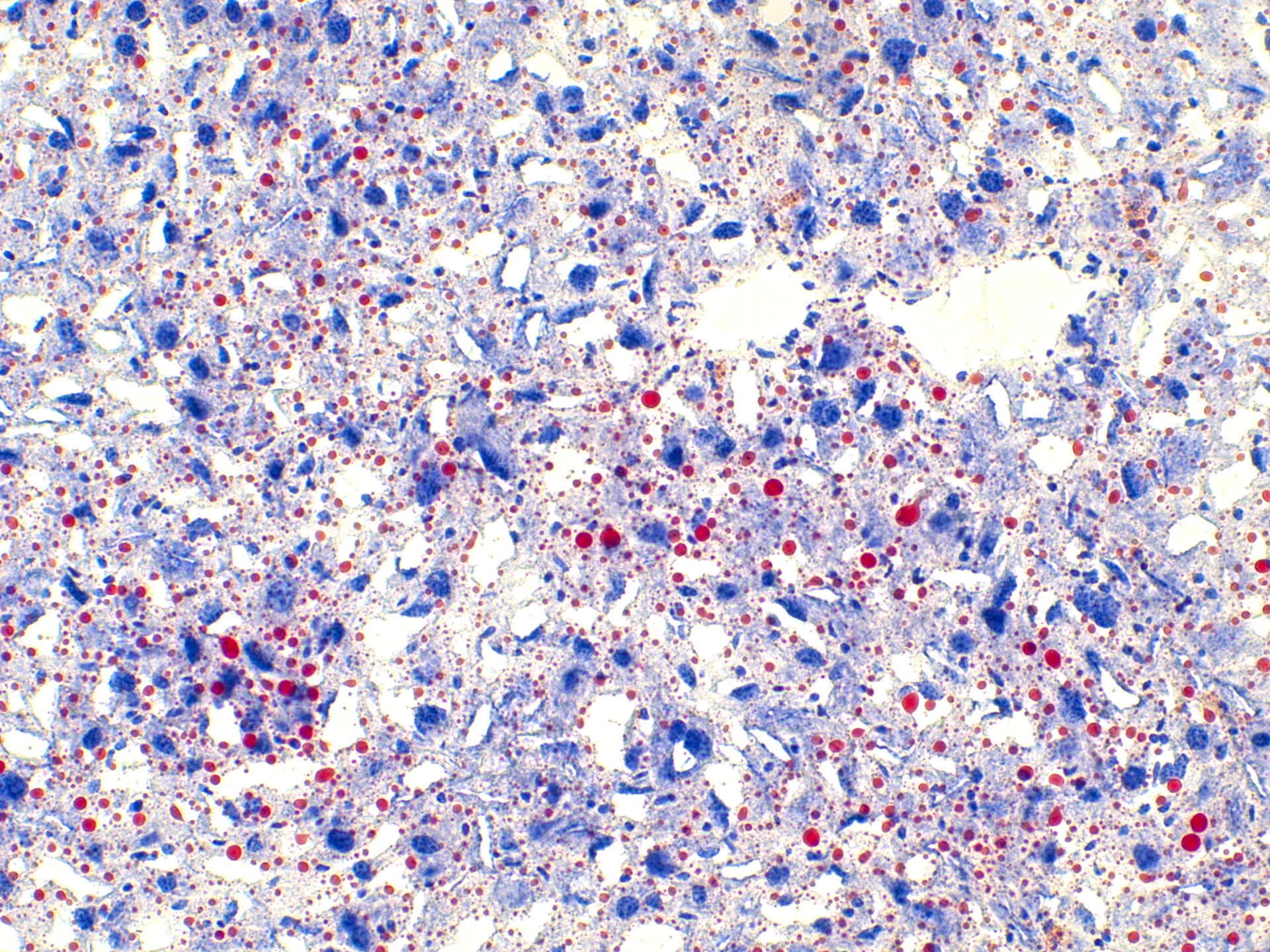

Supplement: Supplementary file 8 [file Data_Sheet_8.ZIP › Fig1.ORred/ORed-STE+LGG.tif]

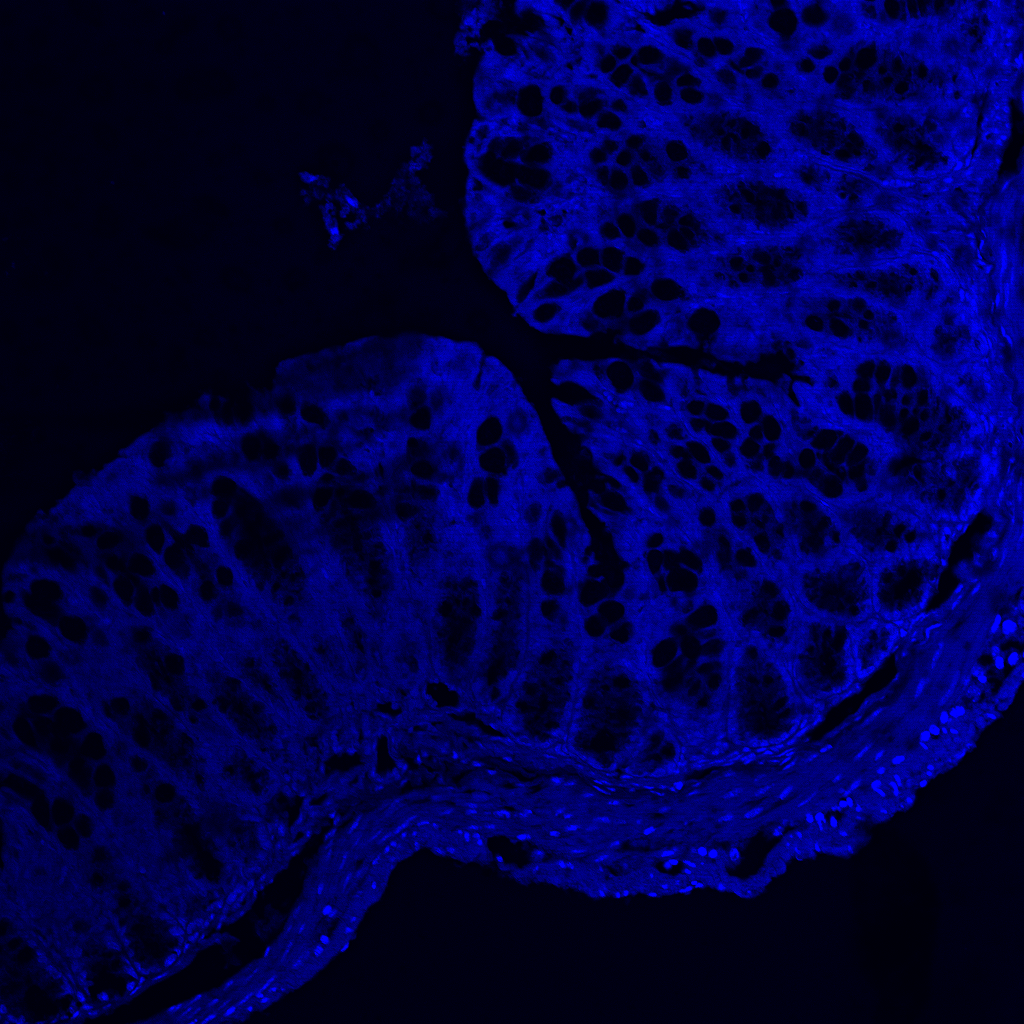

Supplement: Supplementary file 9 [file Data_Sheet_9.ZIP › Figure2/Con_C001T001.tif]

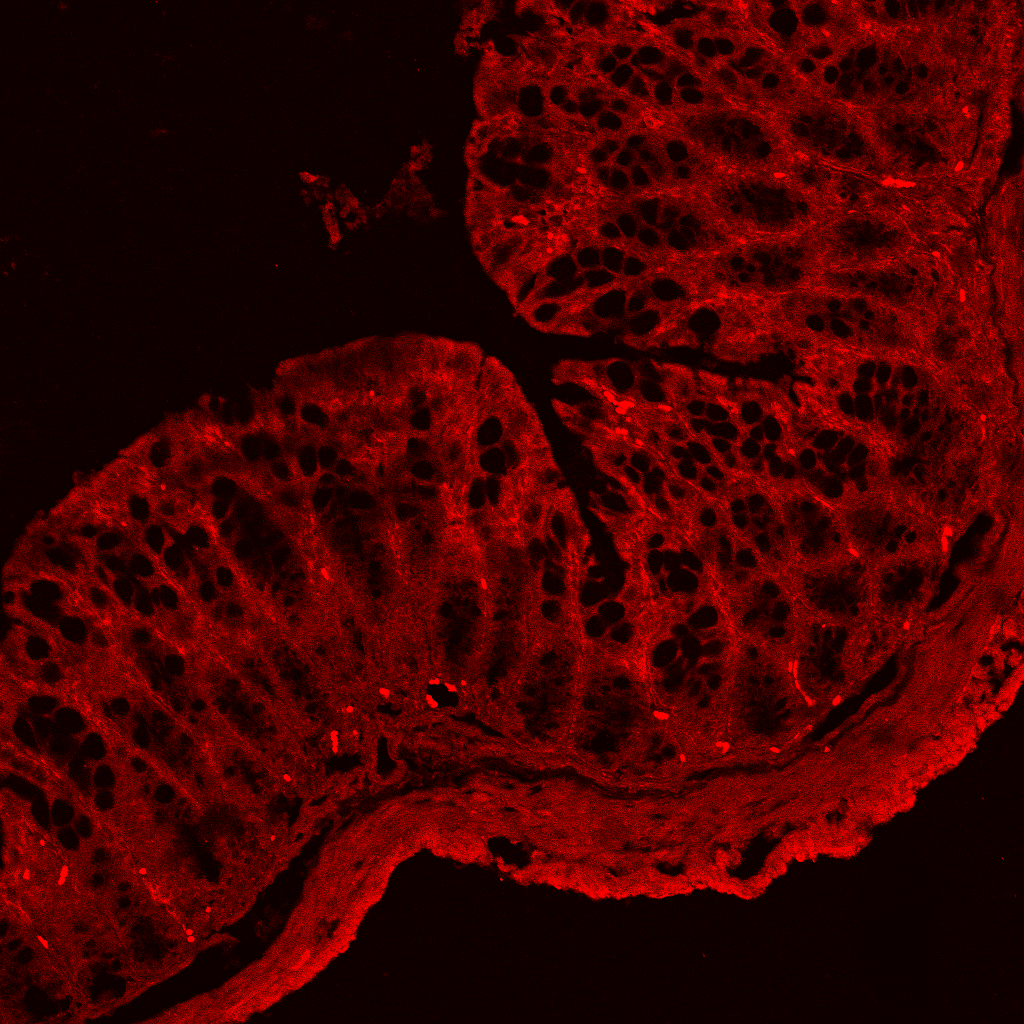

Supplement: Supplementary file 9 [file Data_Sheet_9.ZIP › Figure2/Con_C002T001.tif]

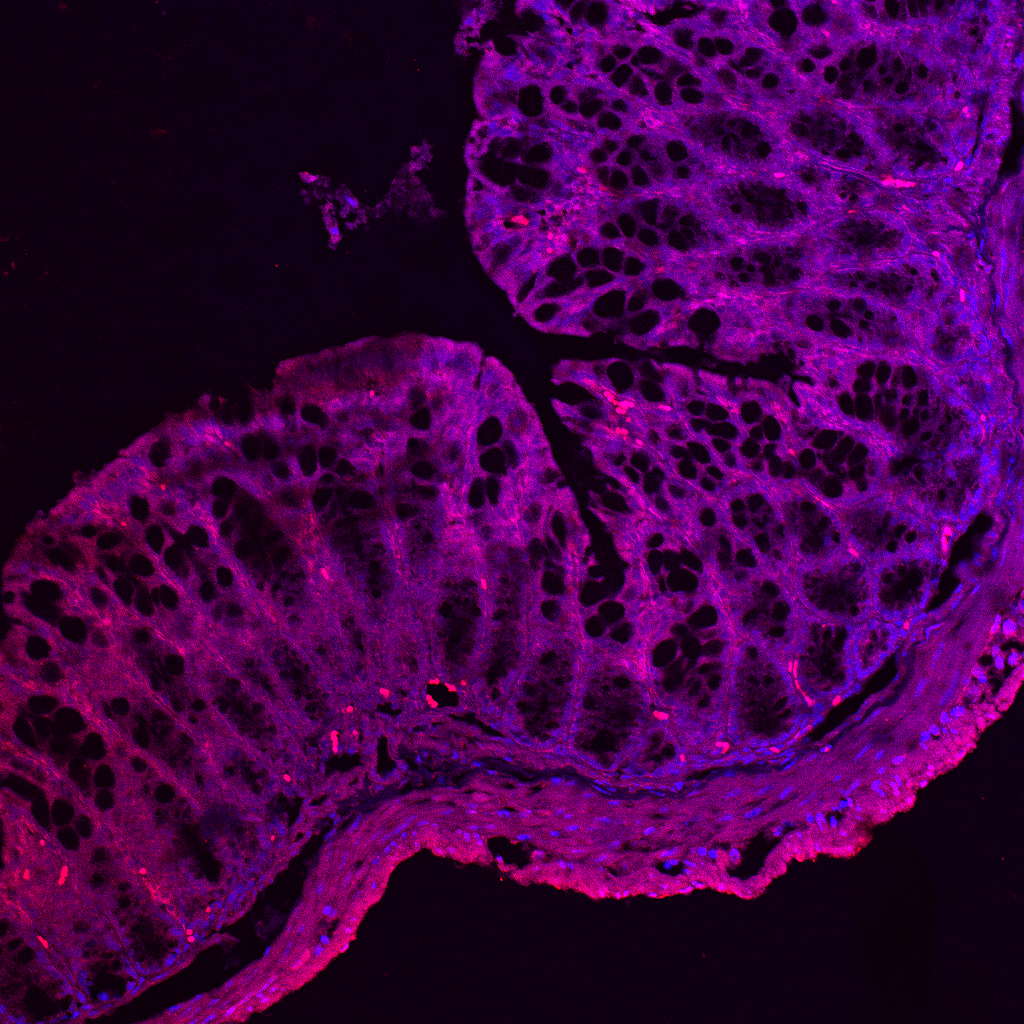

Supplement: Supplementary file 9 [file Data_Sheet_9.ZIP › Figure2/Con_T001.tif]

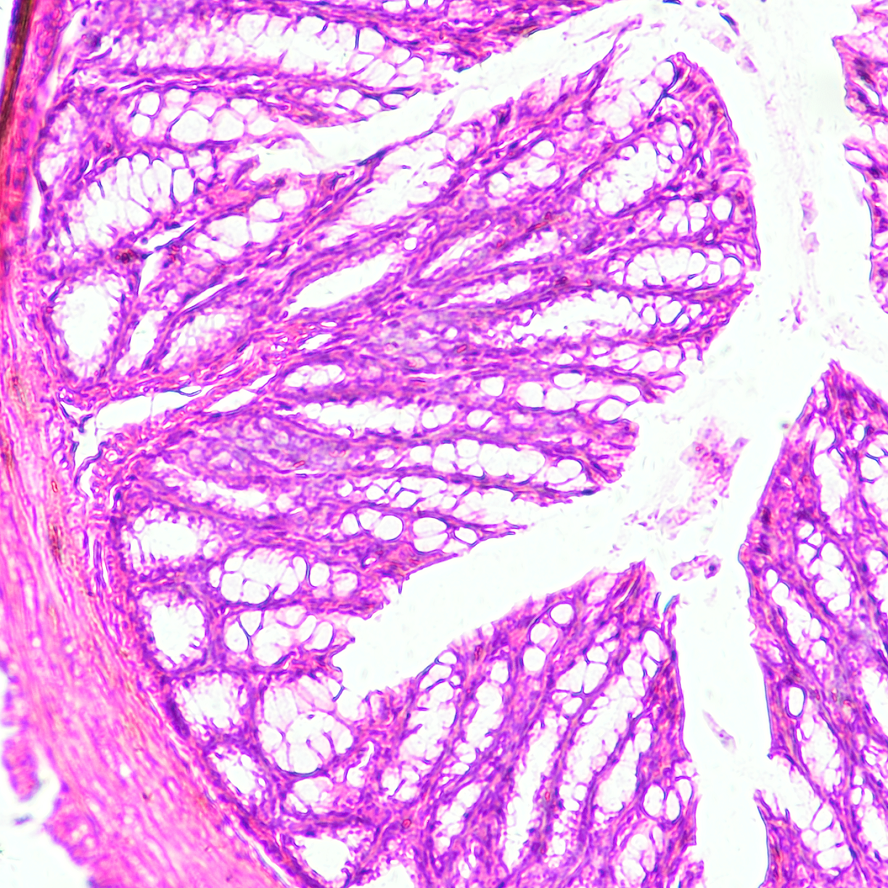

Supplement: Supplementary file 9 [file Data_Sheet_9.ZIP › Figure2/Fig2.A(Con).tif]

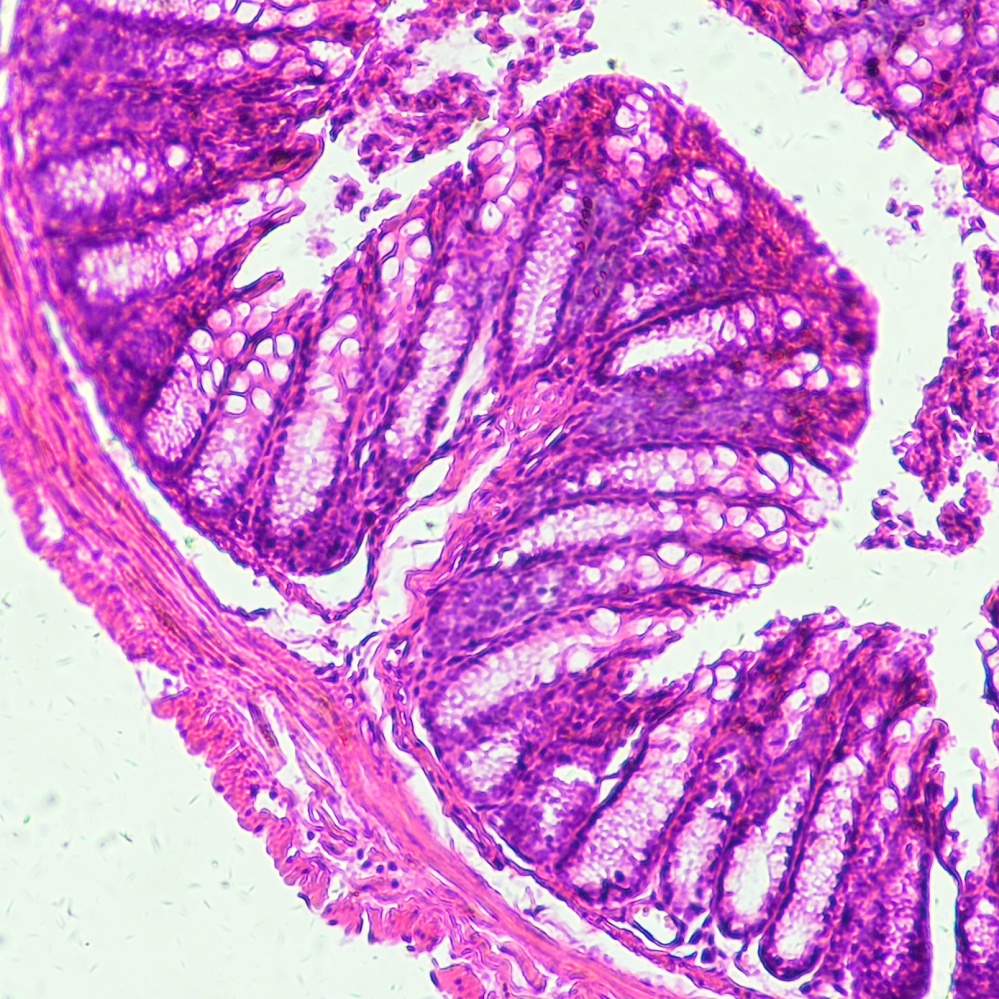

Supplement: Supplementary file 9 [file Data_Sheet_9.ZIP › Figure2/Fig2.A(LGG).tif]

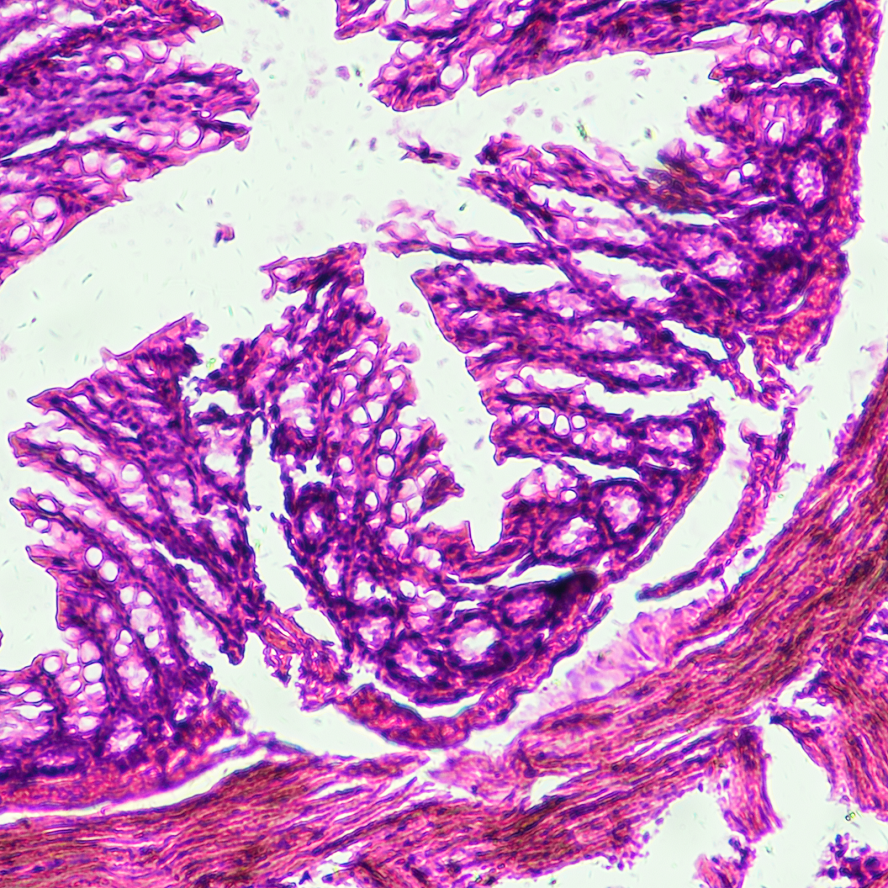

Supplement: Supplementary file 9 [file Data_Sheet_9.ZIP › Figure2/Fig2.A(STE).tif]

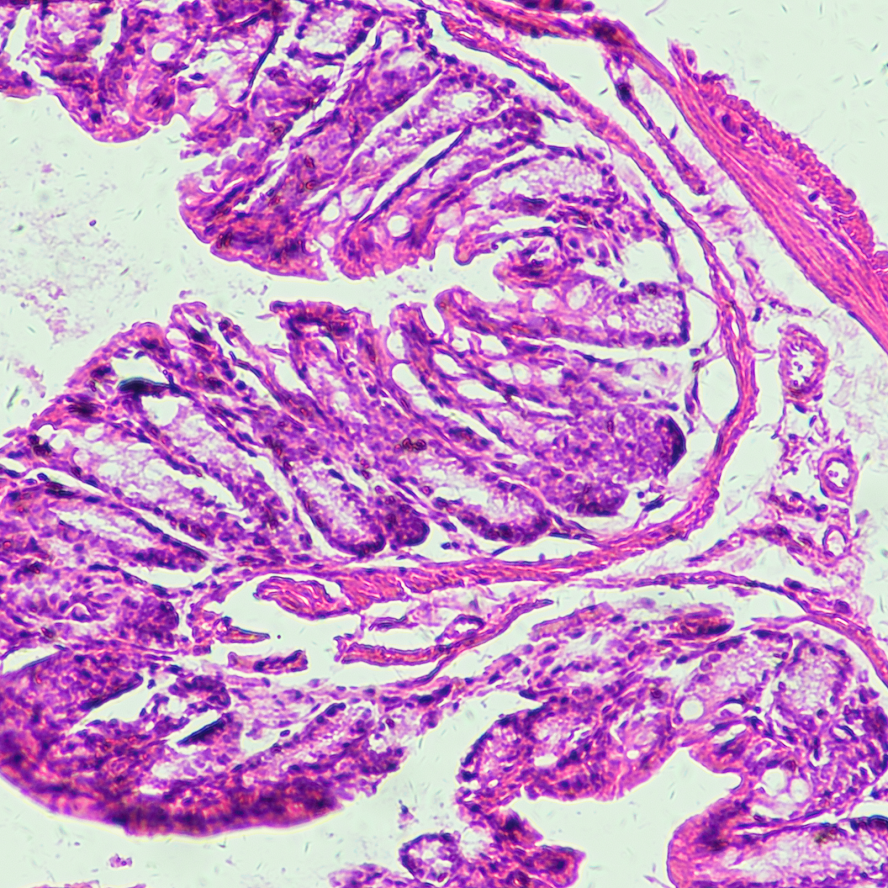

Supplement: Supplementary file 9 [file Data_Sheet_9.ZIP › Figure2/Fig2.A(STE+LGG).tif]

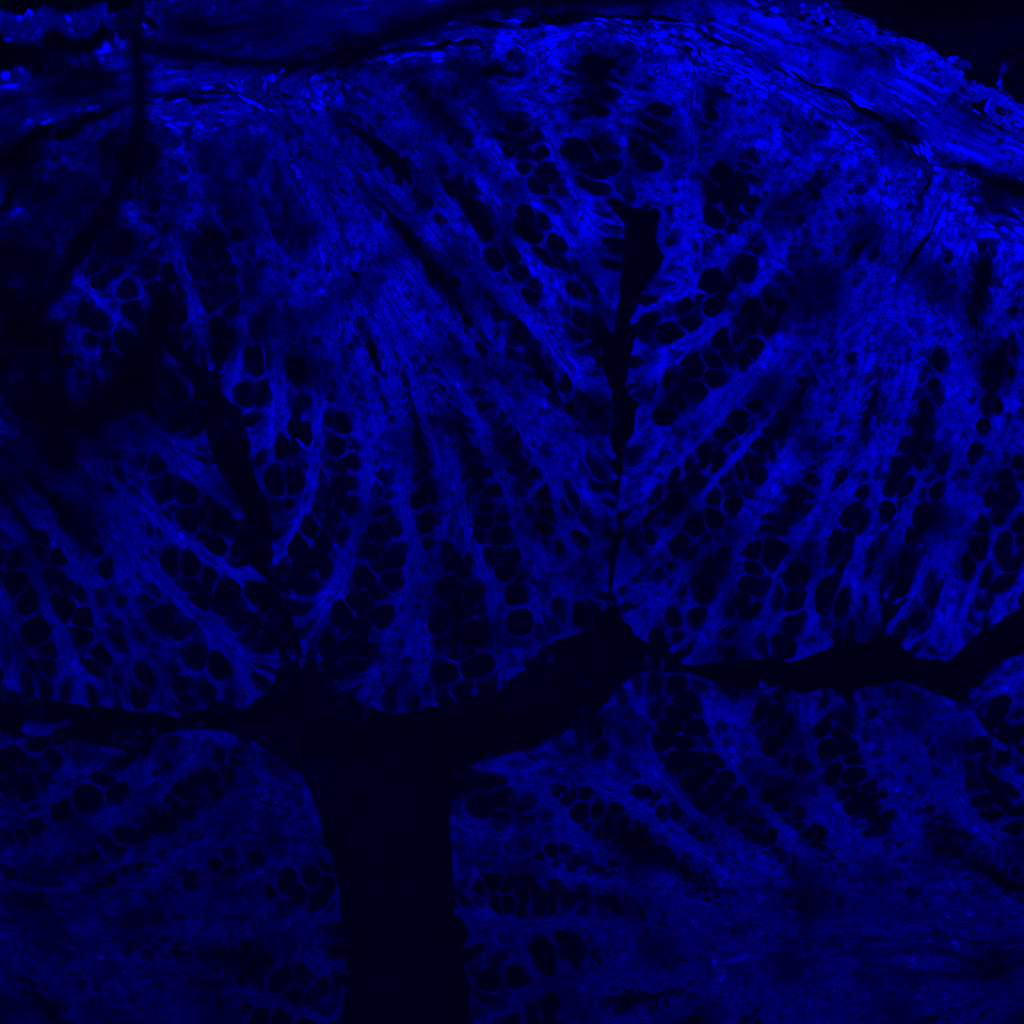

Supplement: Supplementary file 9 [file Data_Sheet_9.ZIP › Figure2/LJ2_C001T001.tif]

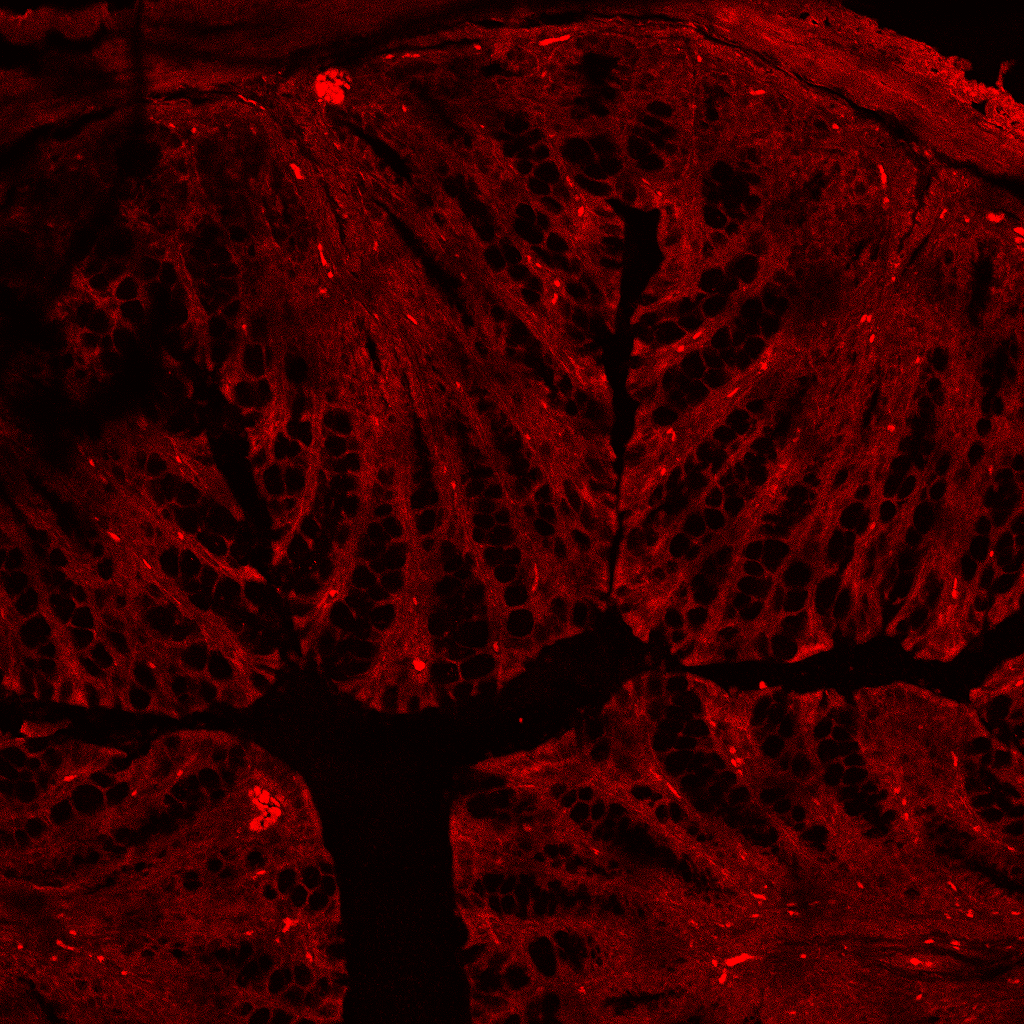

Supplement: Supplementary file 9 [file Data_Sheet_9.ZIP › Figure2/LJ2_C002T001.tif]

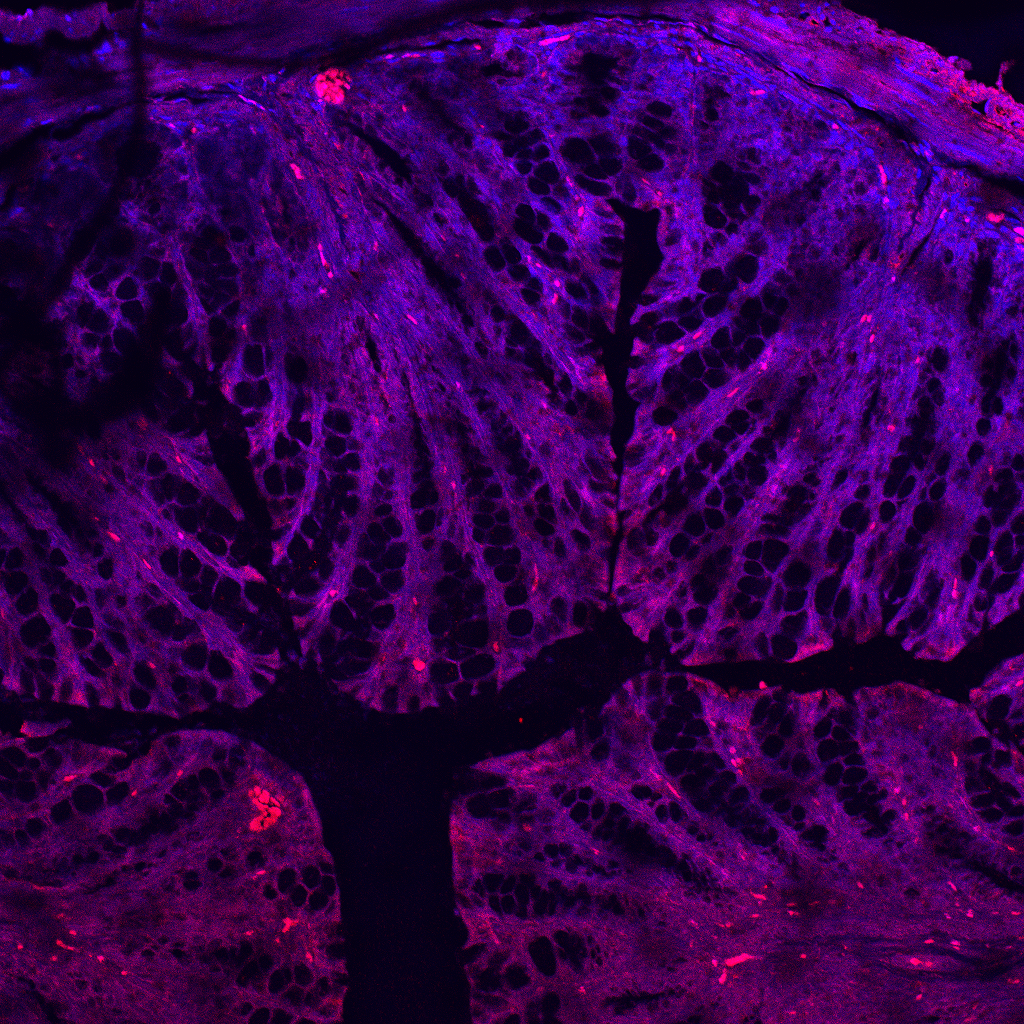

Supplement: Supplementary file 9 [file Data_Sheet_9.ZIP › Figure2/LJ2_T001.tif]

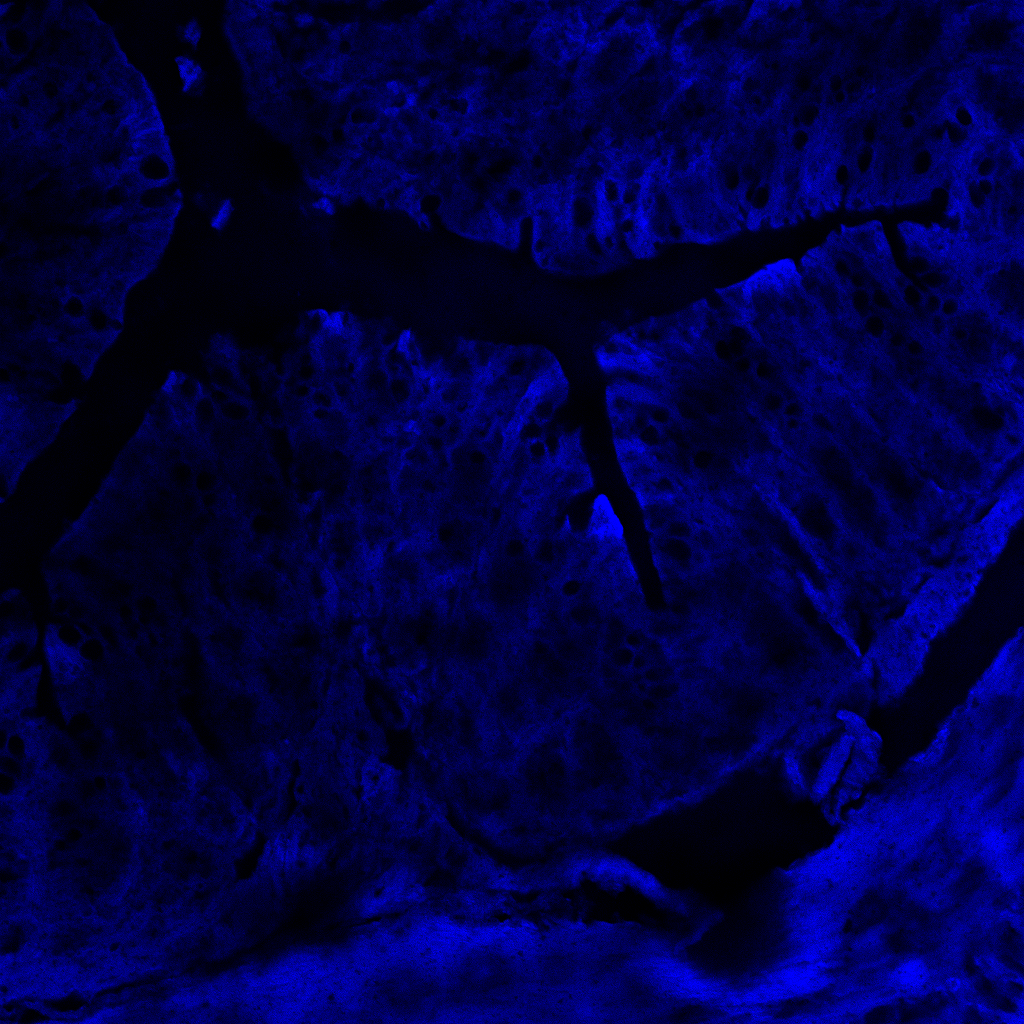

Supplement: Supplementary file 9 [file Data_Sheet_9.ZIP › Figure2/STE2_C001T001.tif]

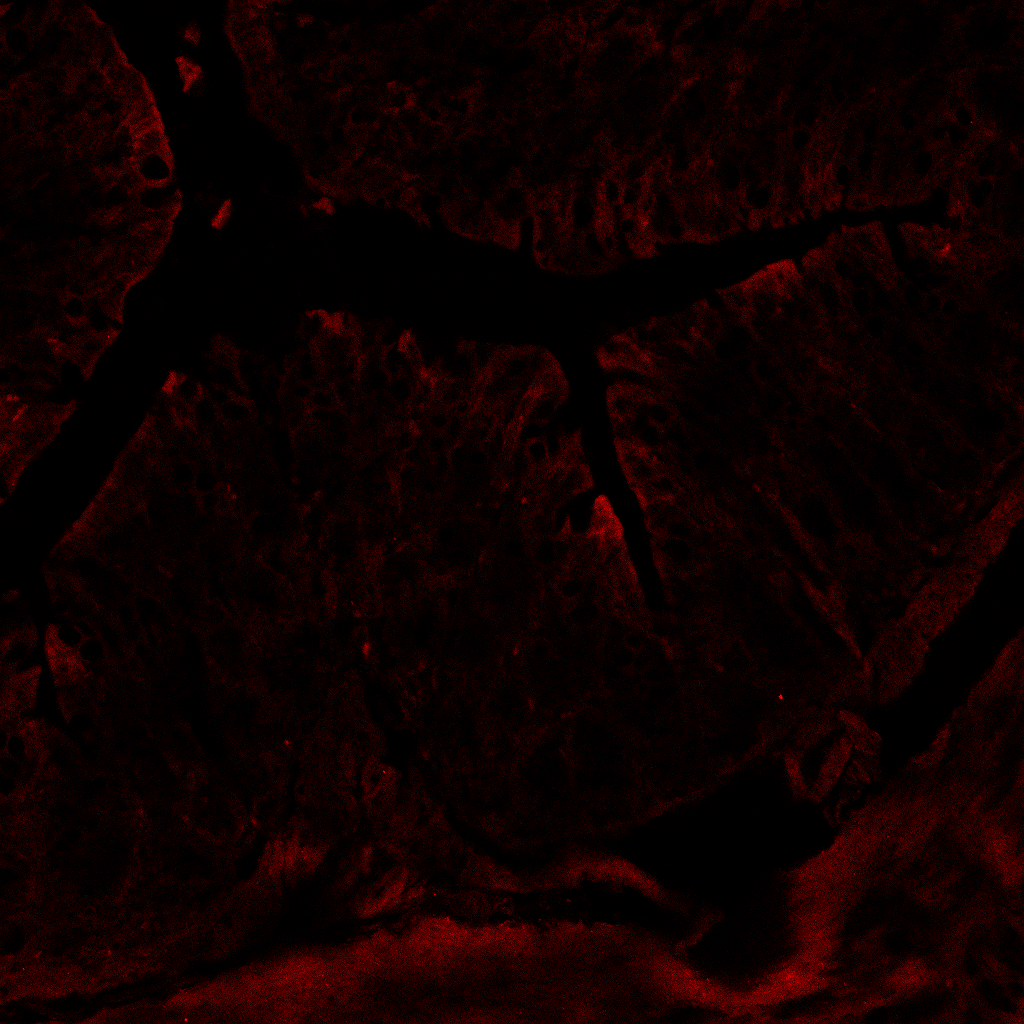

Supplement: Supplementary file 9 [file Data_Sheet_9.ZIP › Figure2/STE2_C002T001.tif]

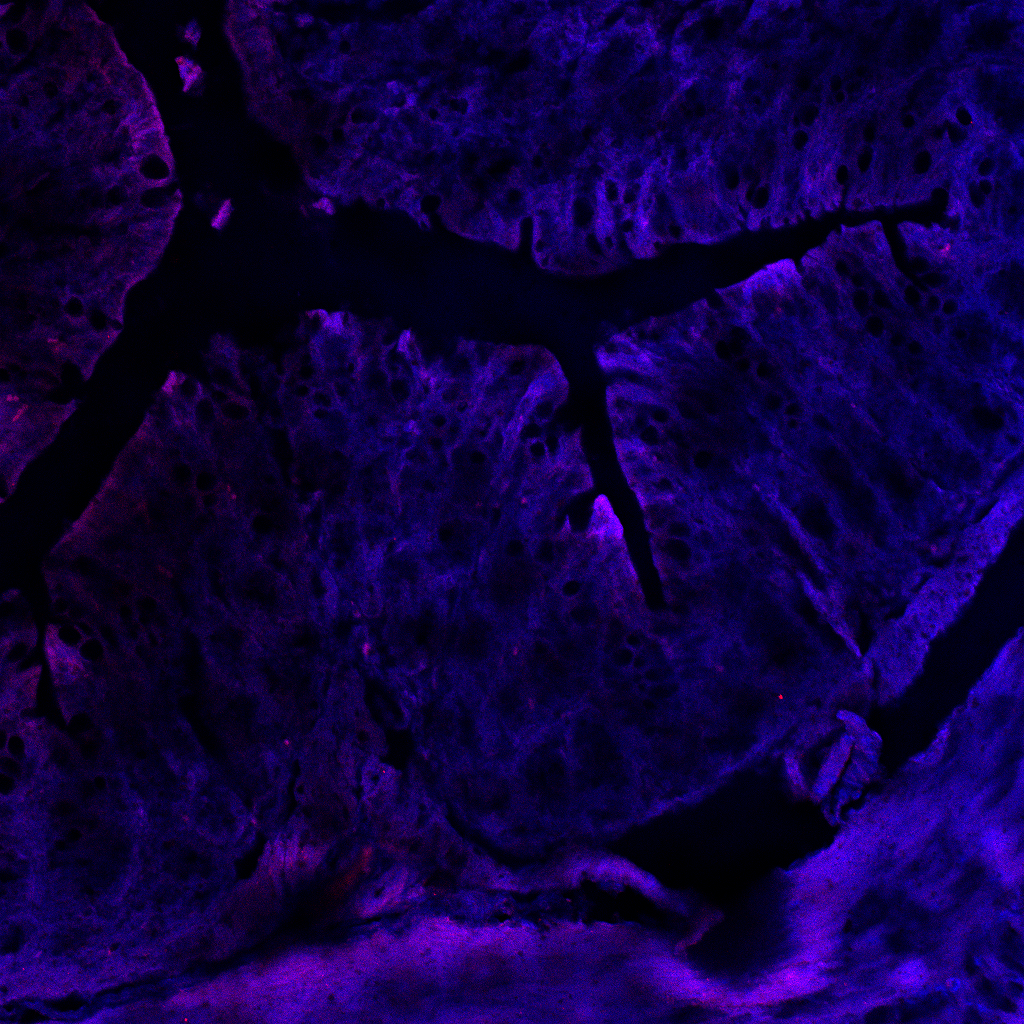

Supplement: Supplementary file 9 [file Data_Sheet_9.ZIP › Figure2/STE2_T001.tif]

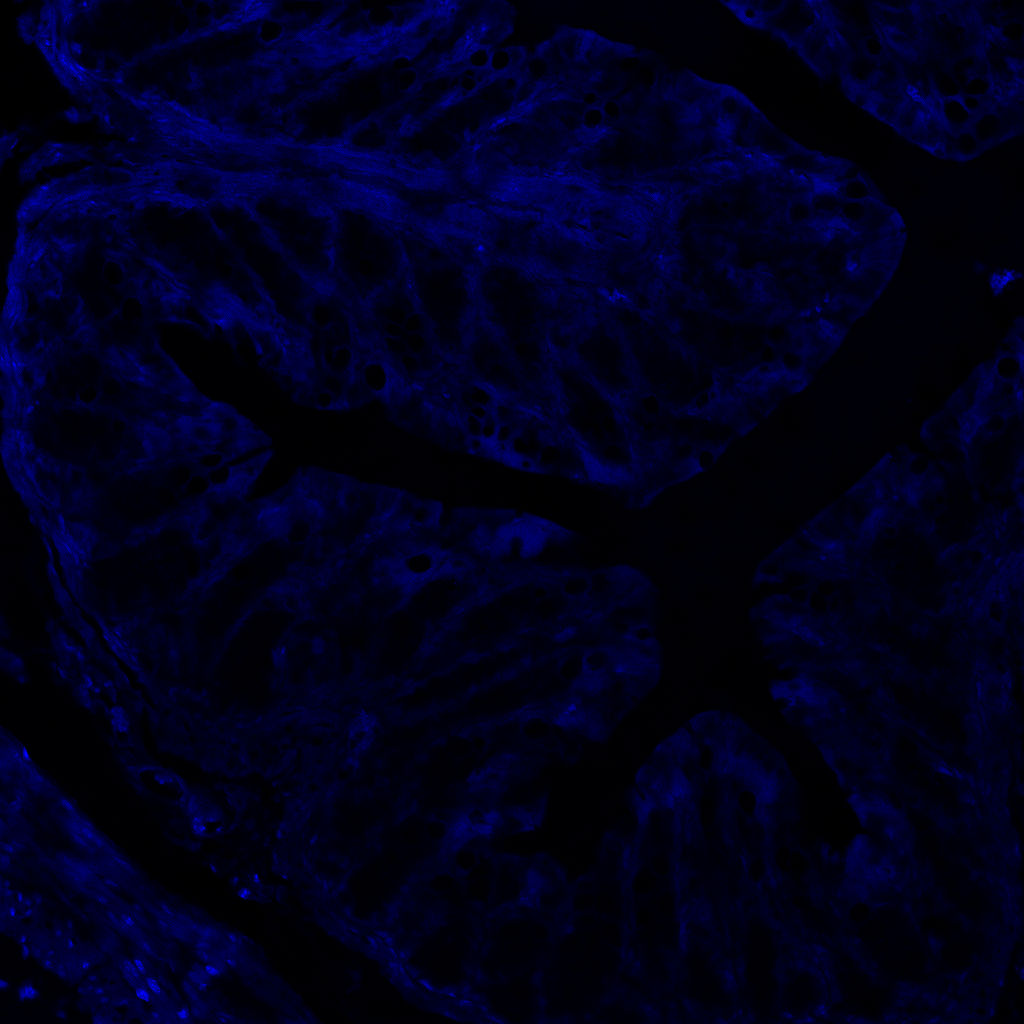

Supplement: Supplementary file 9 [file Data_Sheet_9.ZIP › Figure2/STET4_C001T001.tif]

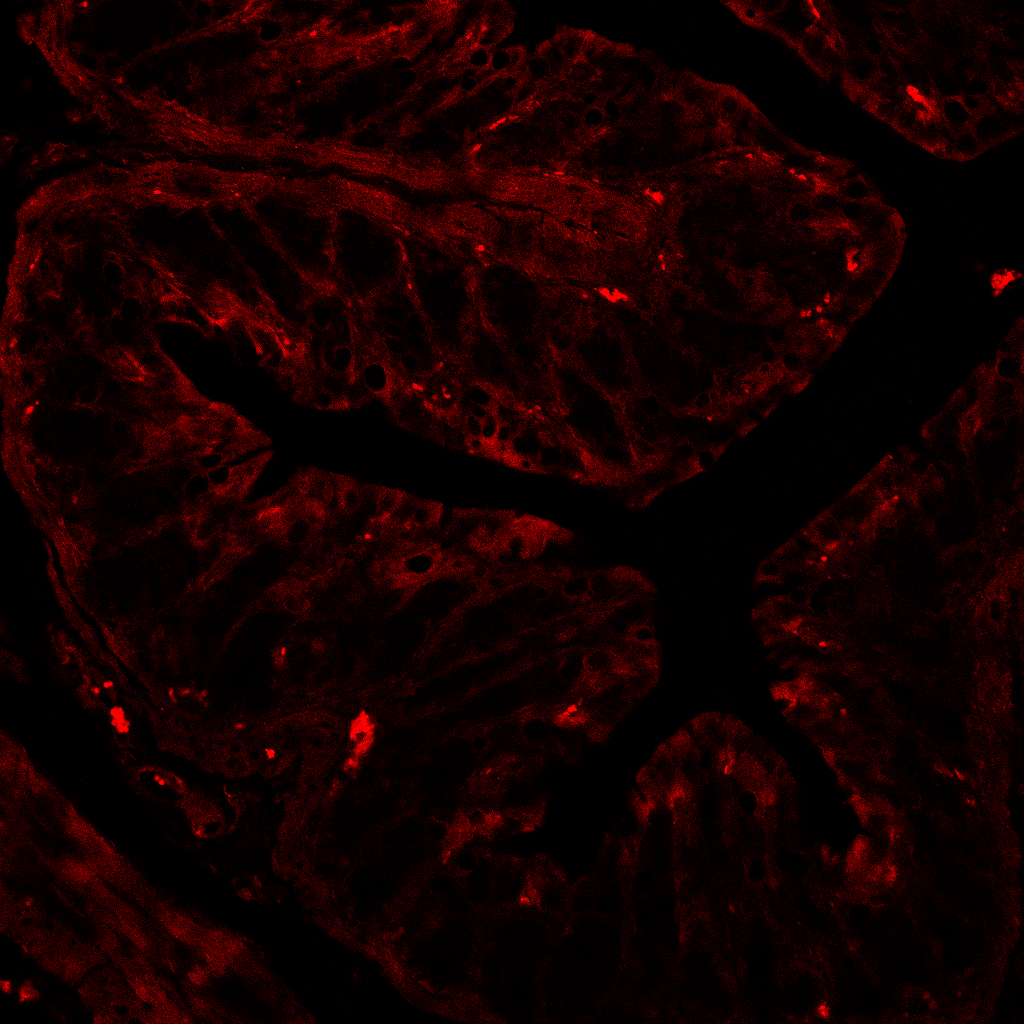

Supplement: Supplementary file 9 [file Data_Sheet_9.ZIP › Figure2/STET4_C002T001.tif]

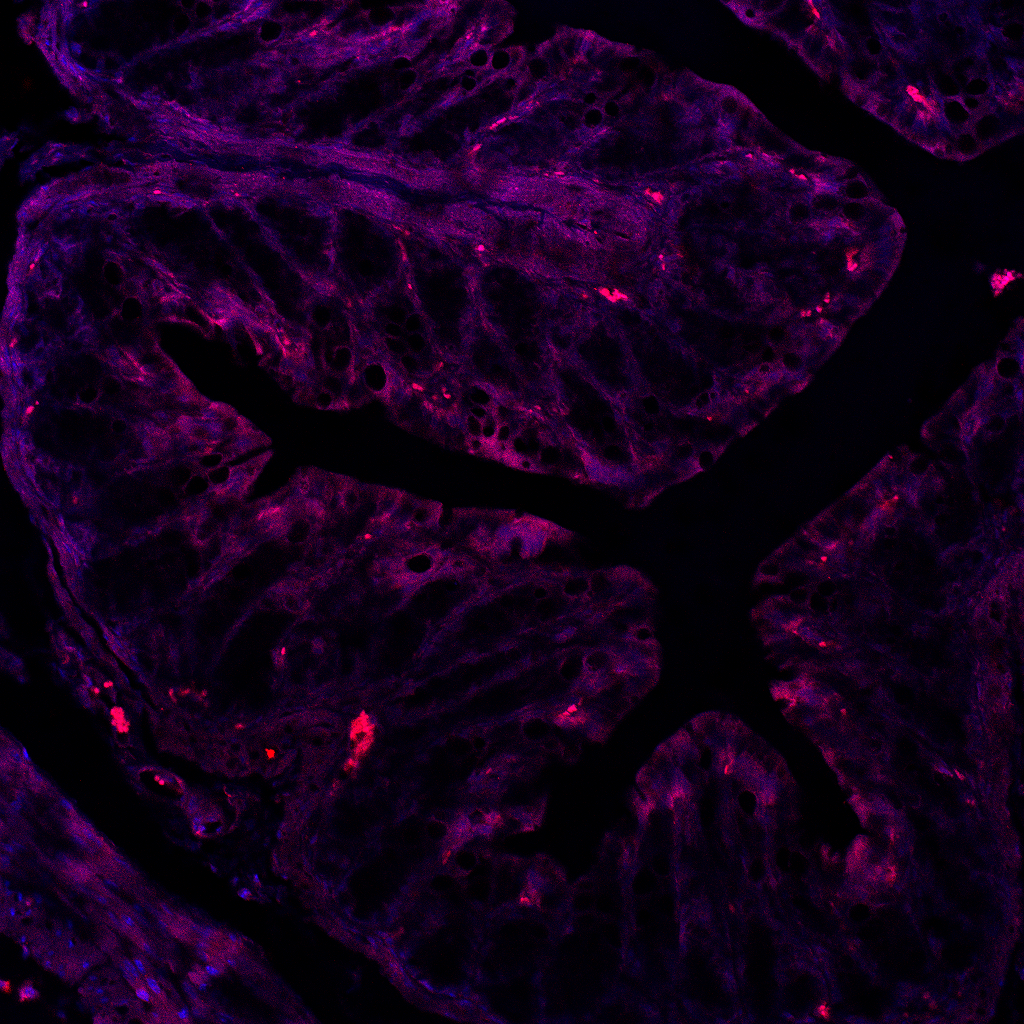

Supplement: Supplementary file 9 [file Data_Sheet_9.ZIP › Figure2/STET4_T001.tif]

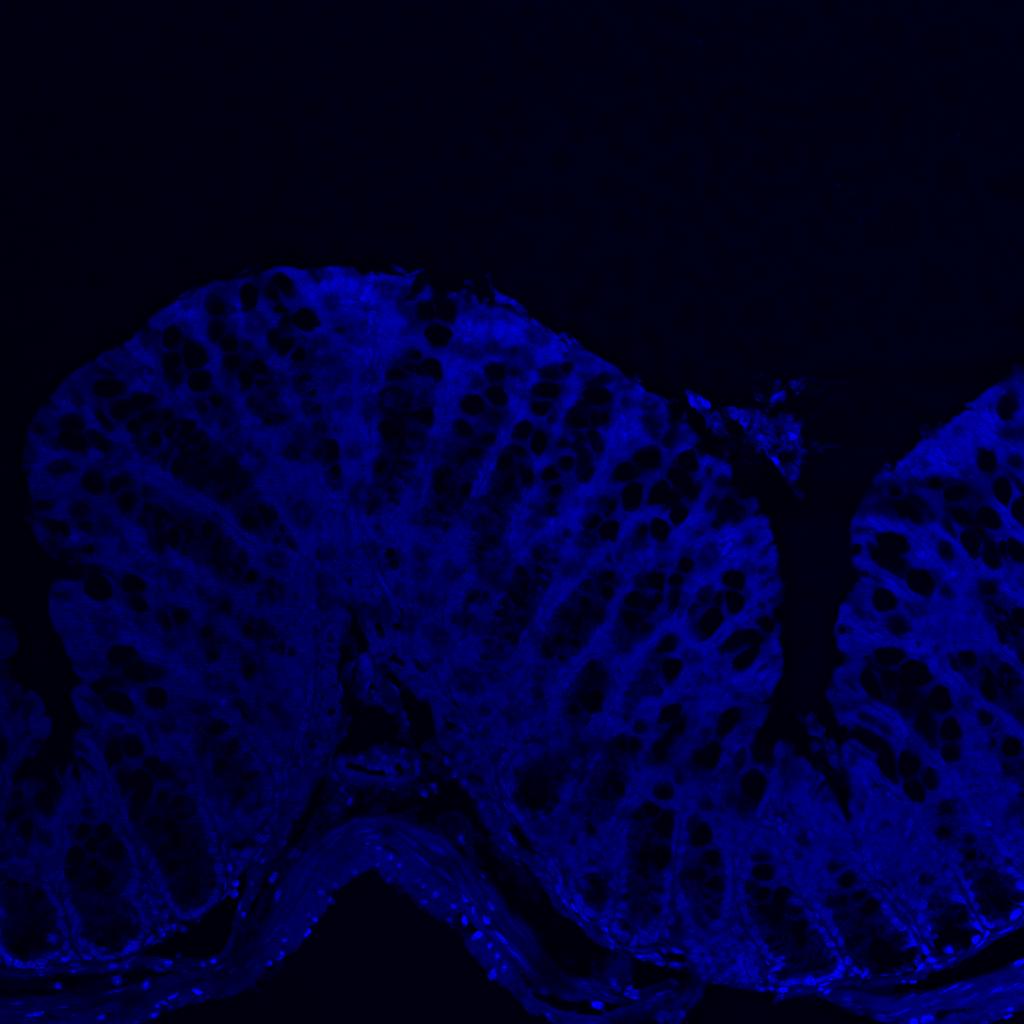

Supplement: Supplementary file 10 [file Data_Sheet_10.ZIP › Figure5/CJ2_C001T001.tif]

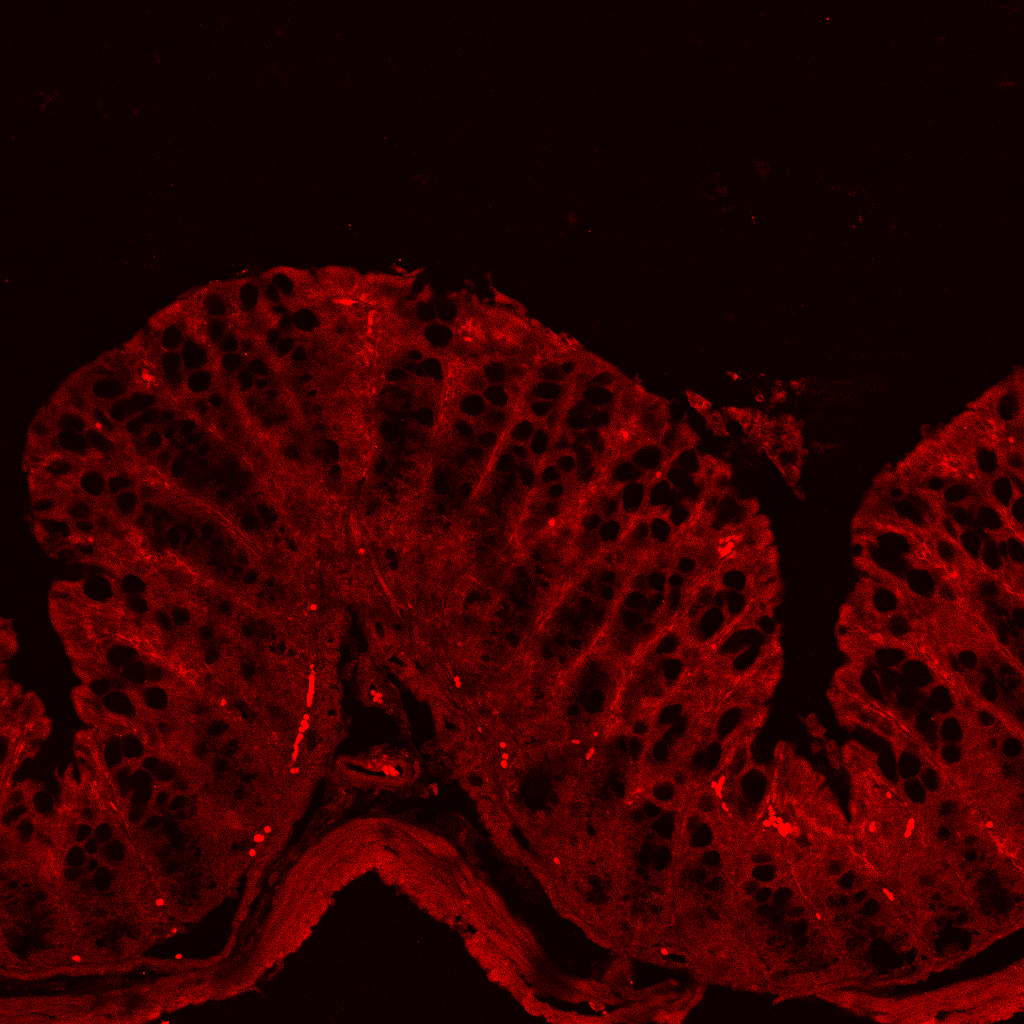

Supplement: Supplementary file 10 [file Data_Sheet_10.ZIP › Figure5/CJ2_C002T001.tif]

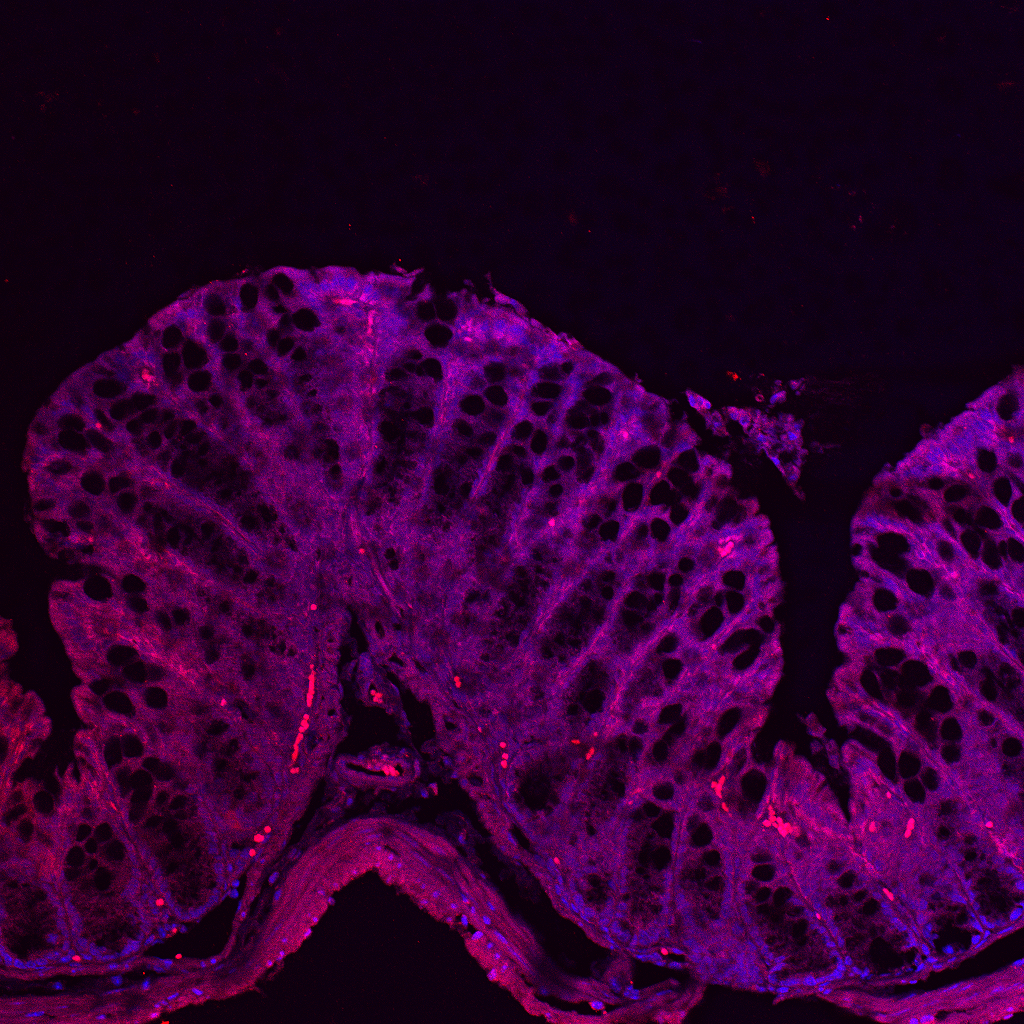

Supplement: Supplementary file 10 [file Data_Sheet_10.ZIP › Figure5/CJ2_T001.tif]

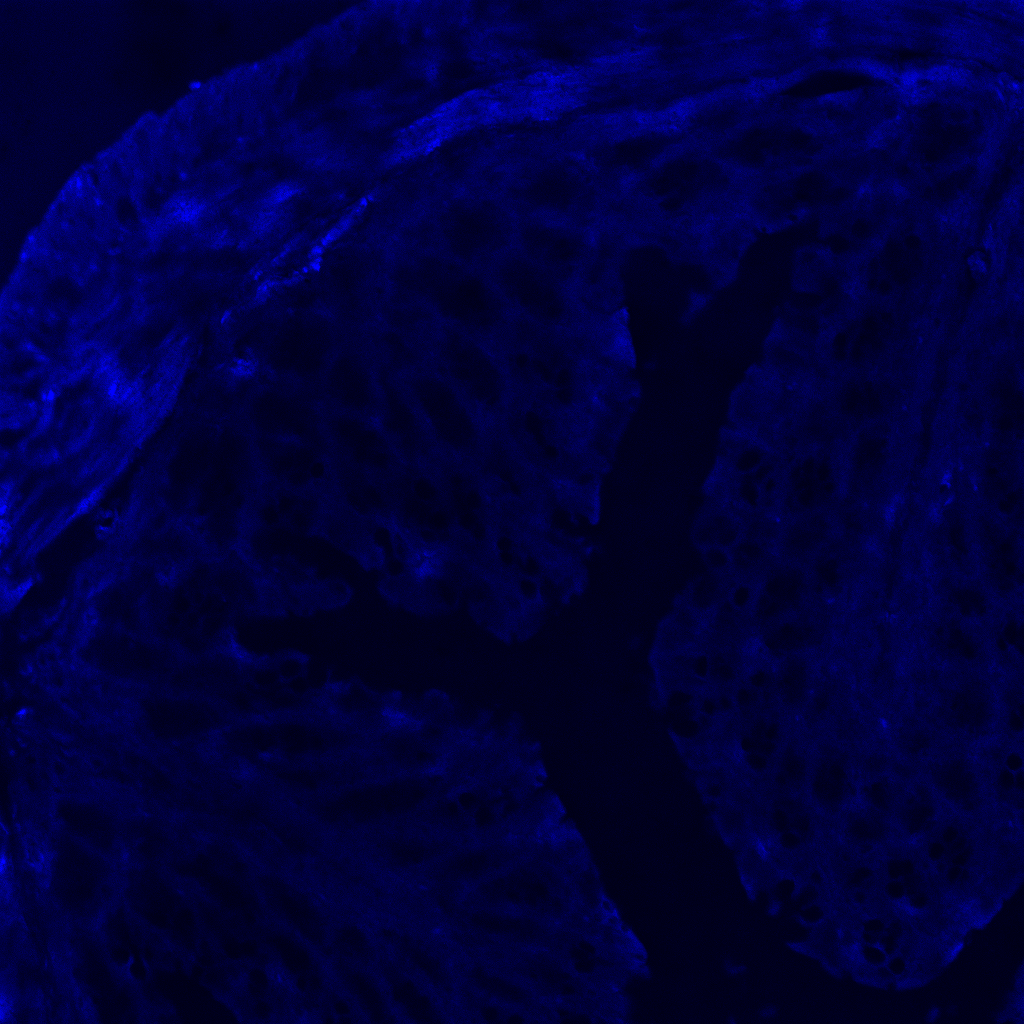

Supplement: Supplementary file 10 [file Data_Sheet_10.ZIP › Figure5/FIB4_C001T001.tif]

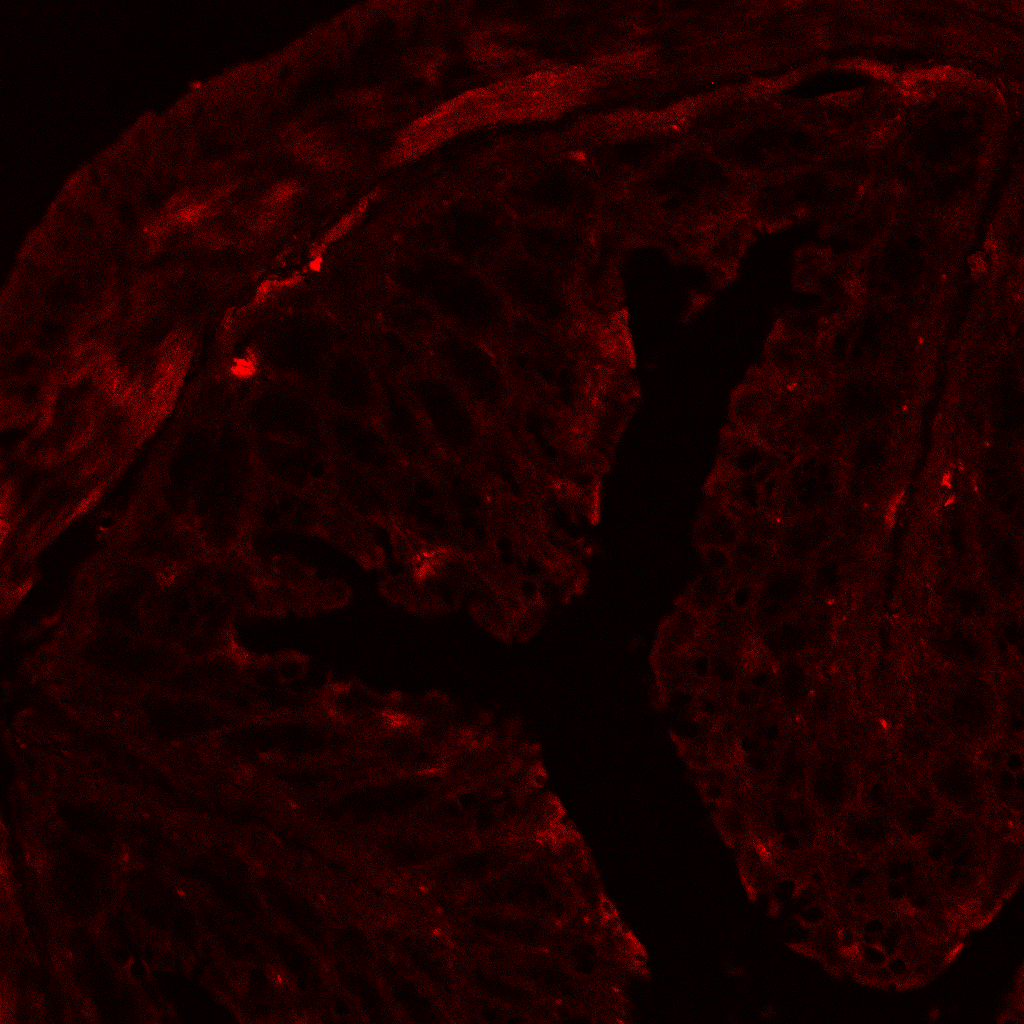

Supplement: Supplementary file 10 [file Data_Sheet_10.ZIP › Figure5/FIB4_C002T001.tif]

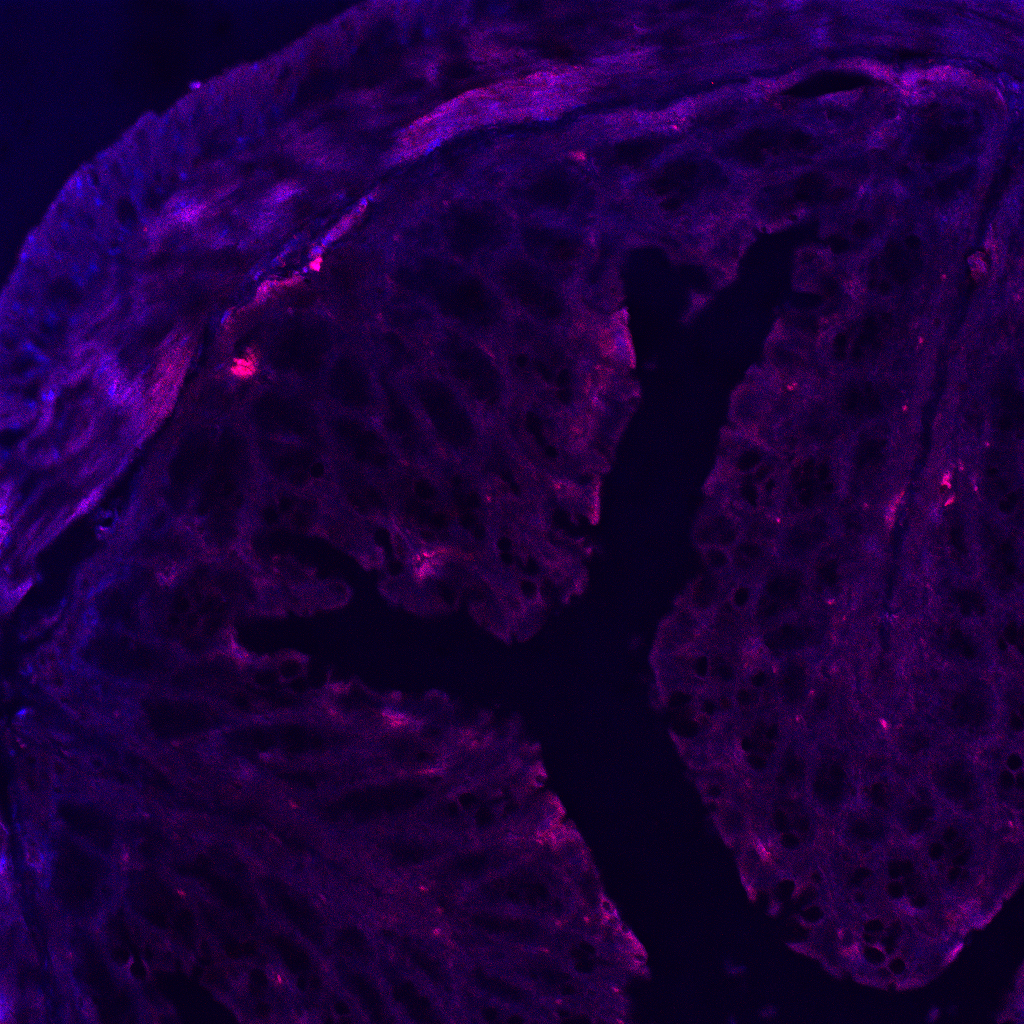

Supplement: Supplementary file 10 [file Data_Sheet_10.ZIP › Figure5/FIB4_T001.tif]

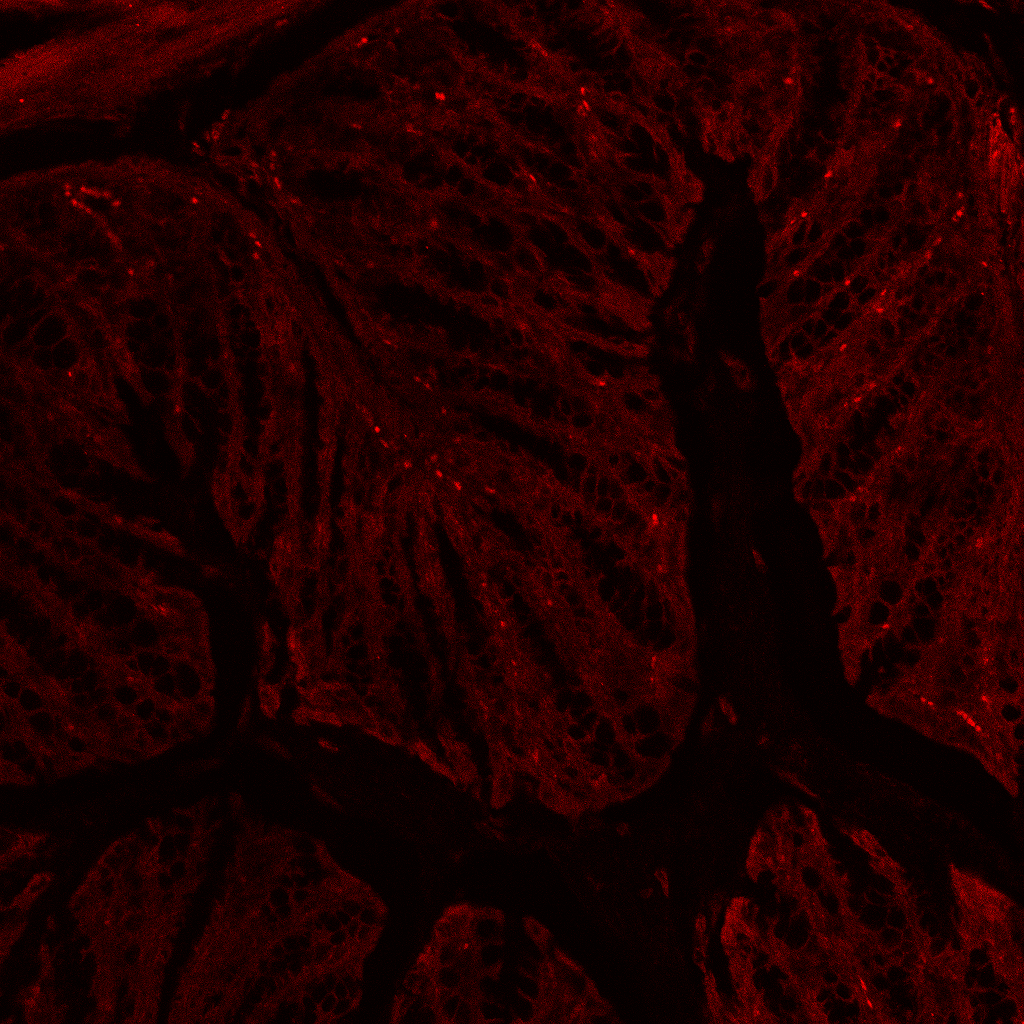

Supplement: Supplementary file 10 [file Data_Sheet_10.ZIP › Figure5/FIBT_C001T001.tif]

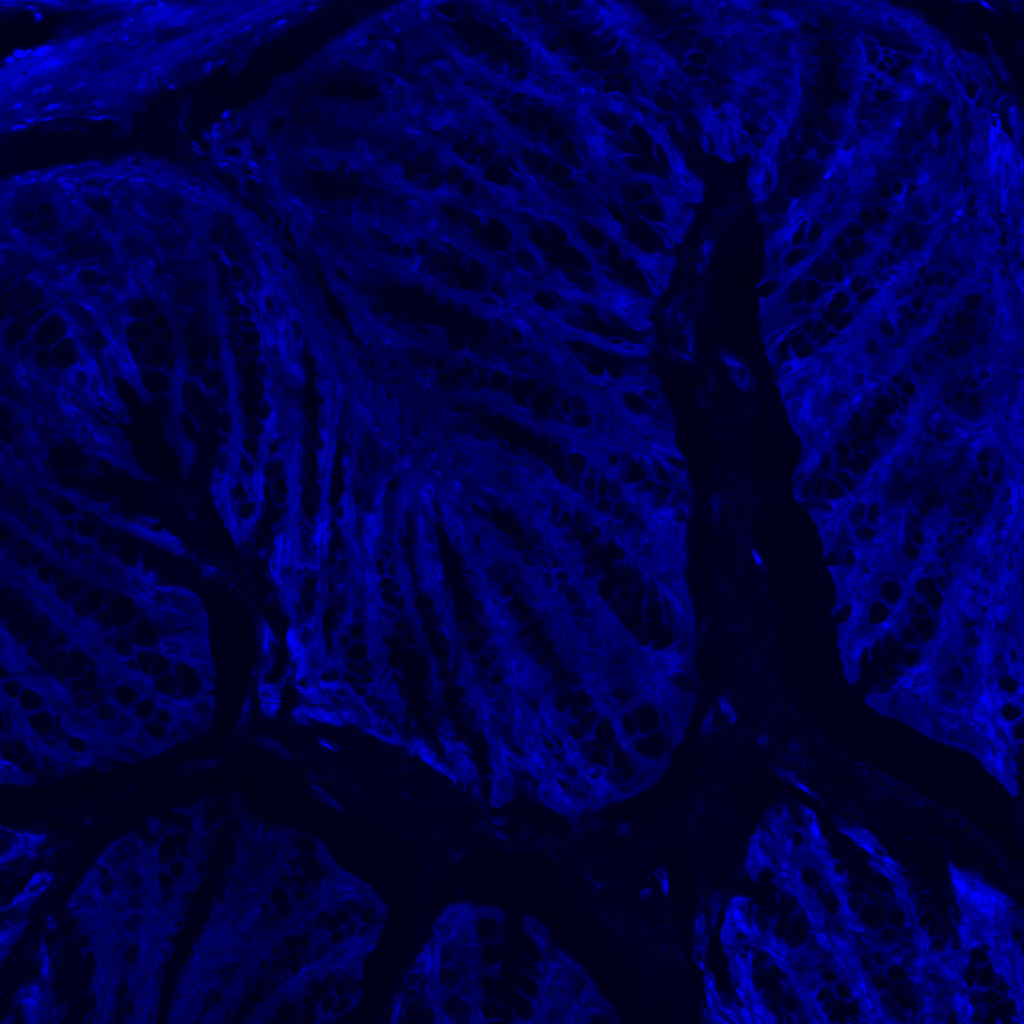

Supplement: Supplementary file 10 [file Data_Sheet_10.ZIP › Figure5/FIBT_C002T001.tif]

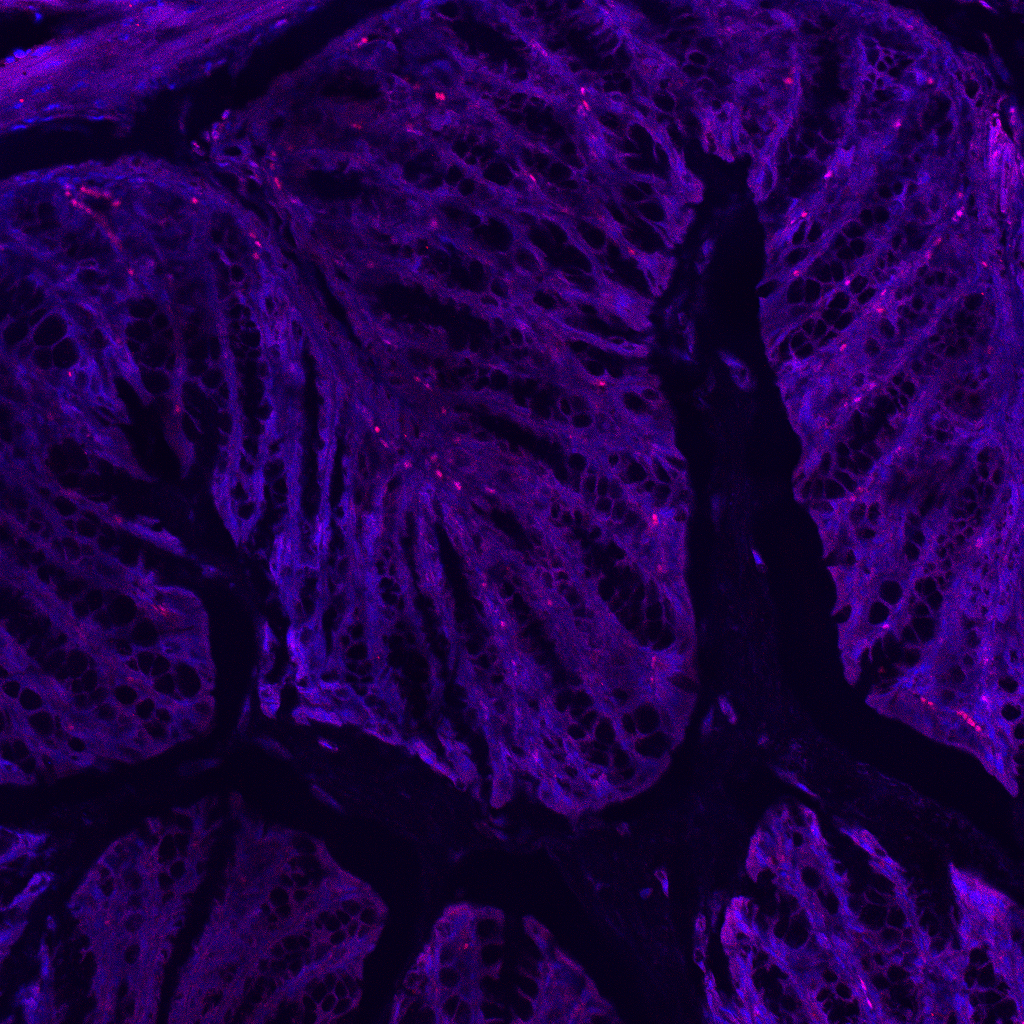

Supplement: Supplementary file 10 [file Data_Sheet_10.ZIP › Figure5/FIBT_T001.tif]

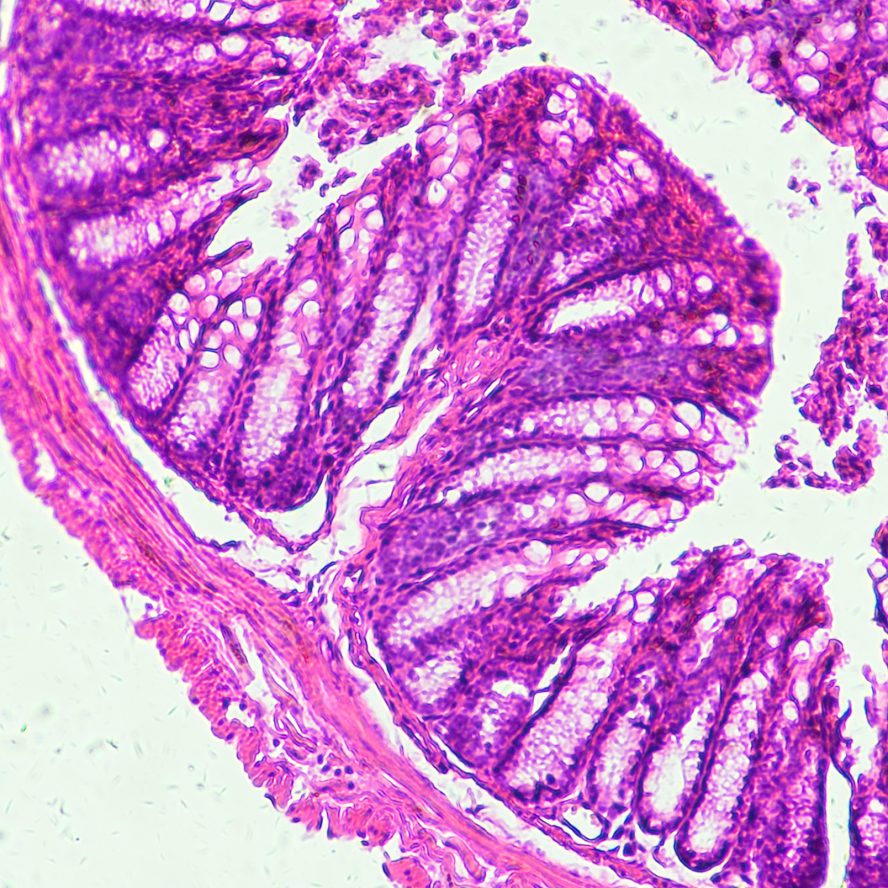

Supplement: Supplementary file 10 [file Data_Sheet_10.ZIP › Figure5/Fig5.A(Con).tif]

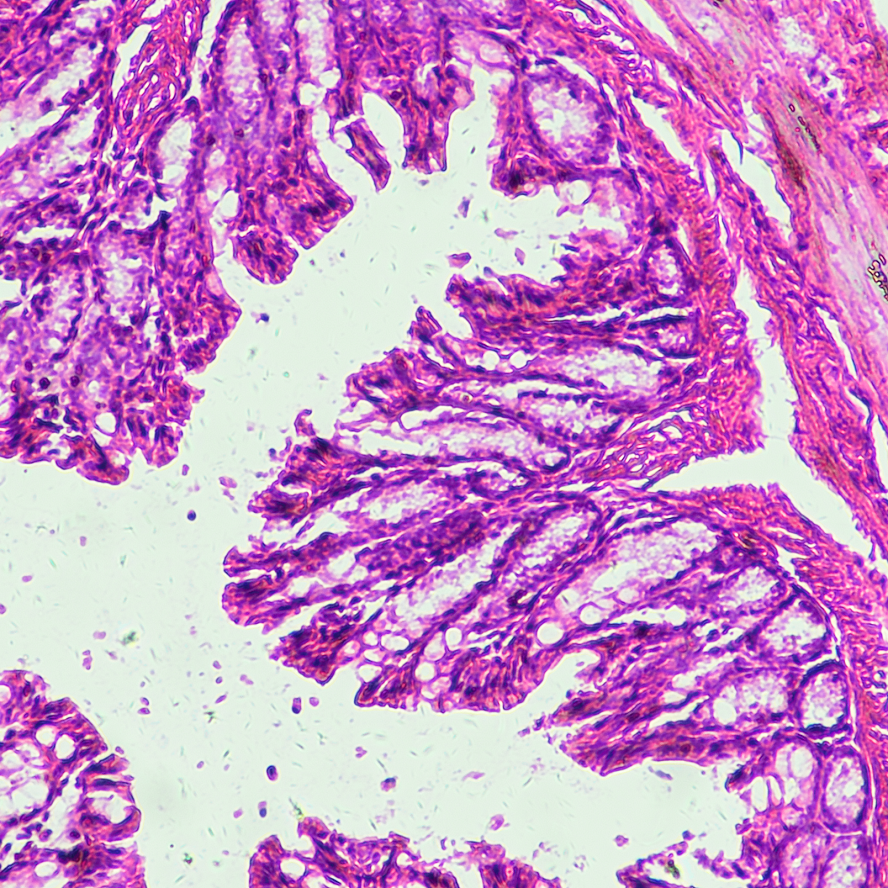

Supplement: Supplementary file 10 [file Data_Sheet_10.ZIP › Figure5/Fig5.A(FIB).tif]

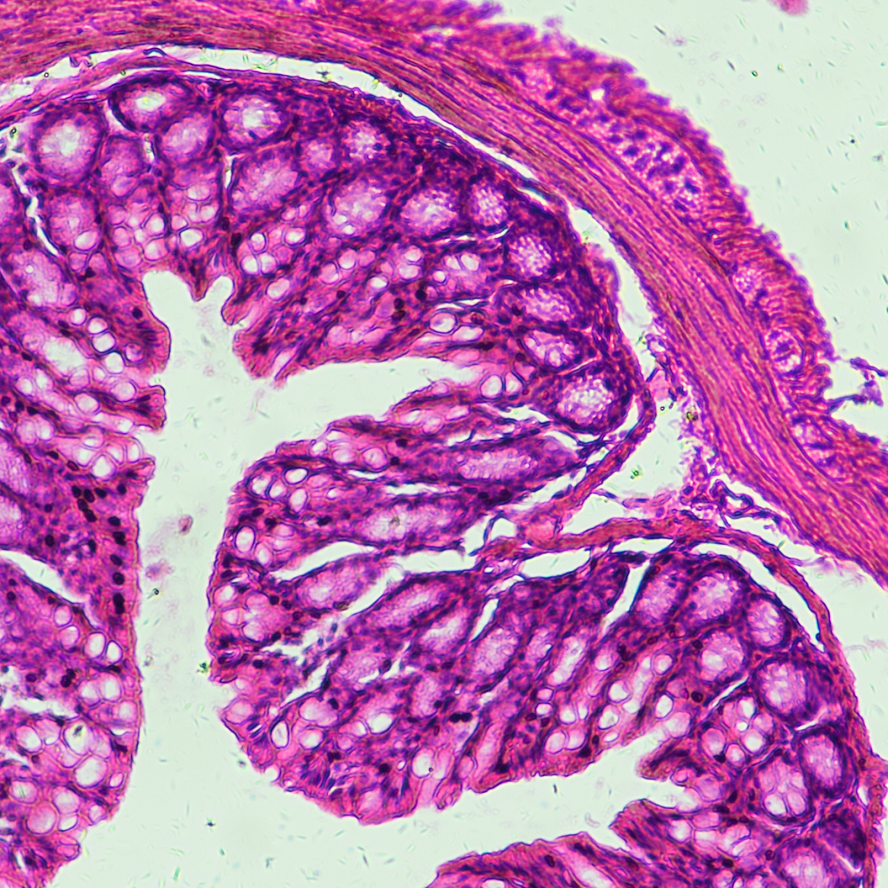

Supplement: Supplementary file 10 [file Data_Sheet_10.ZIP › Figure5/Fig5.A(FIB+LGG).tif]

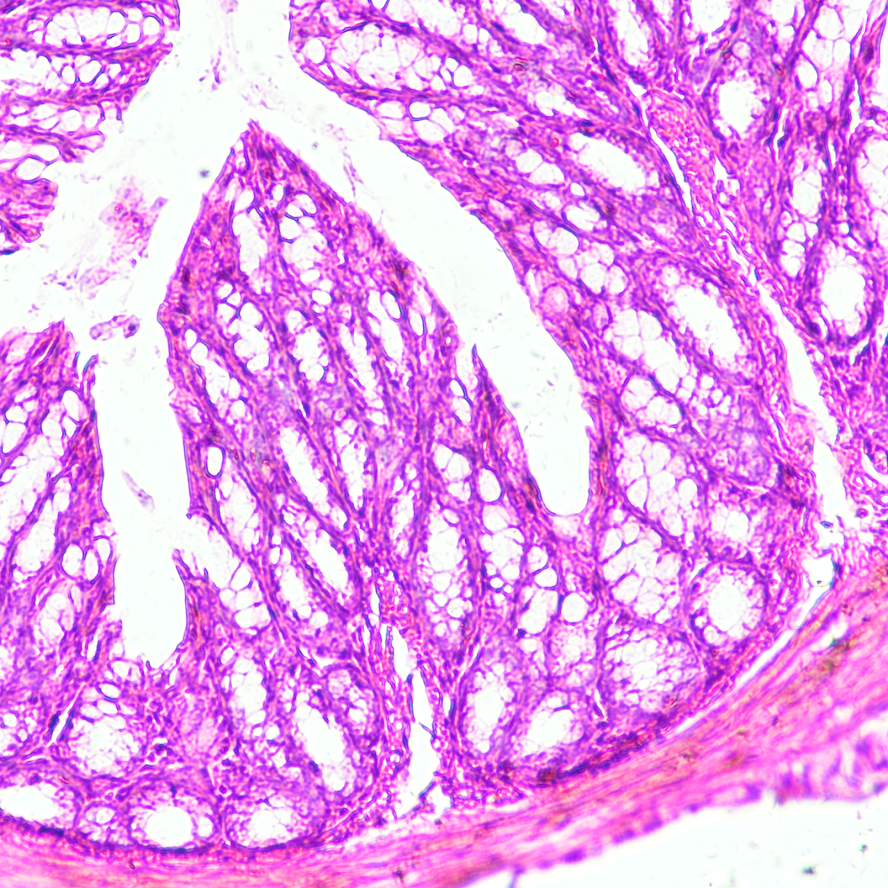

Supplement: Supplementary file 10 [file Data_Sheet_10.ZIP › Figure5/Fig5.A(LGG).tif]

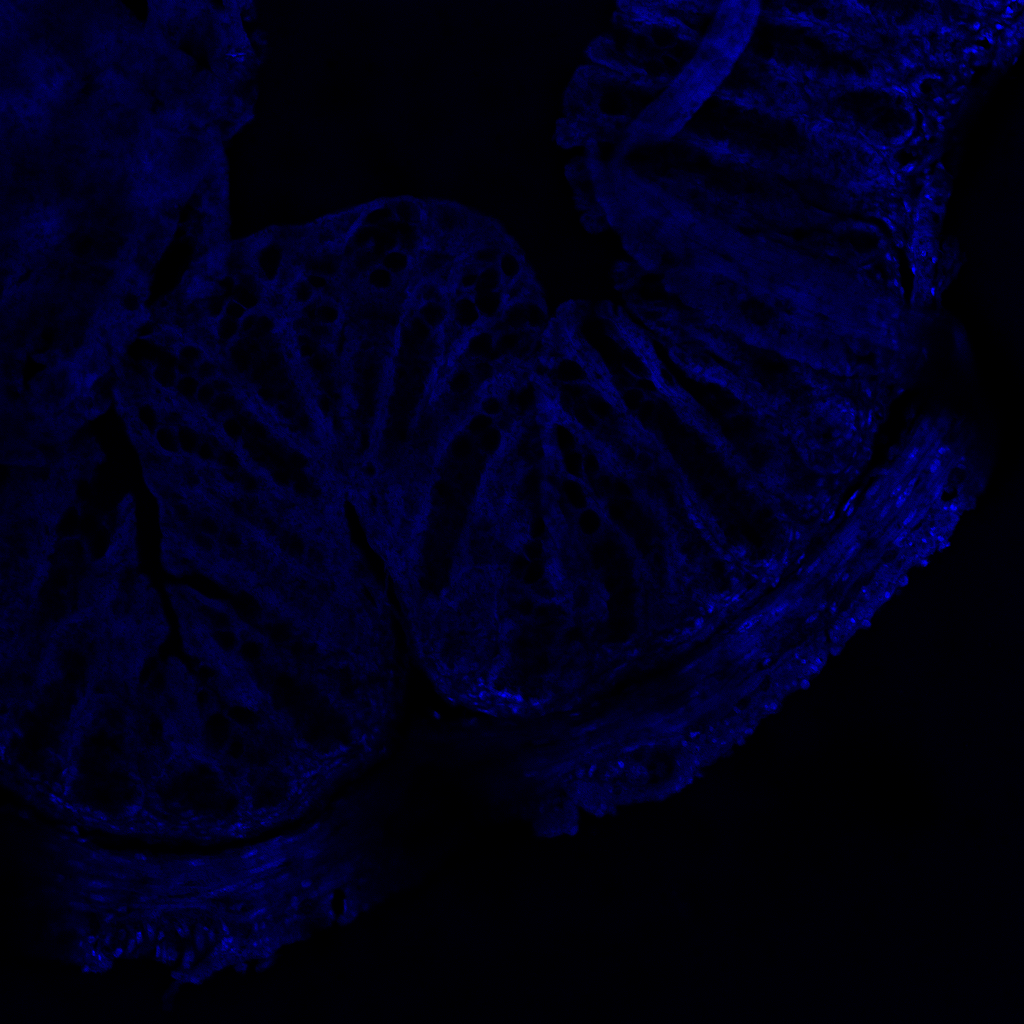

Supplement: Supplementary file 10 [file Data_Sheet_10.ZIP › Figure5/LGG_C001T001.tif]

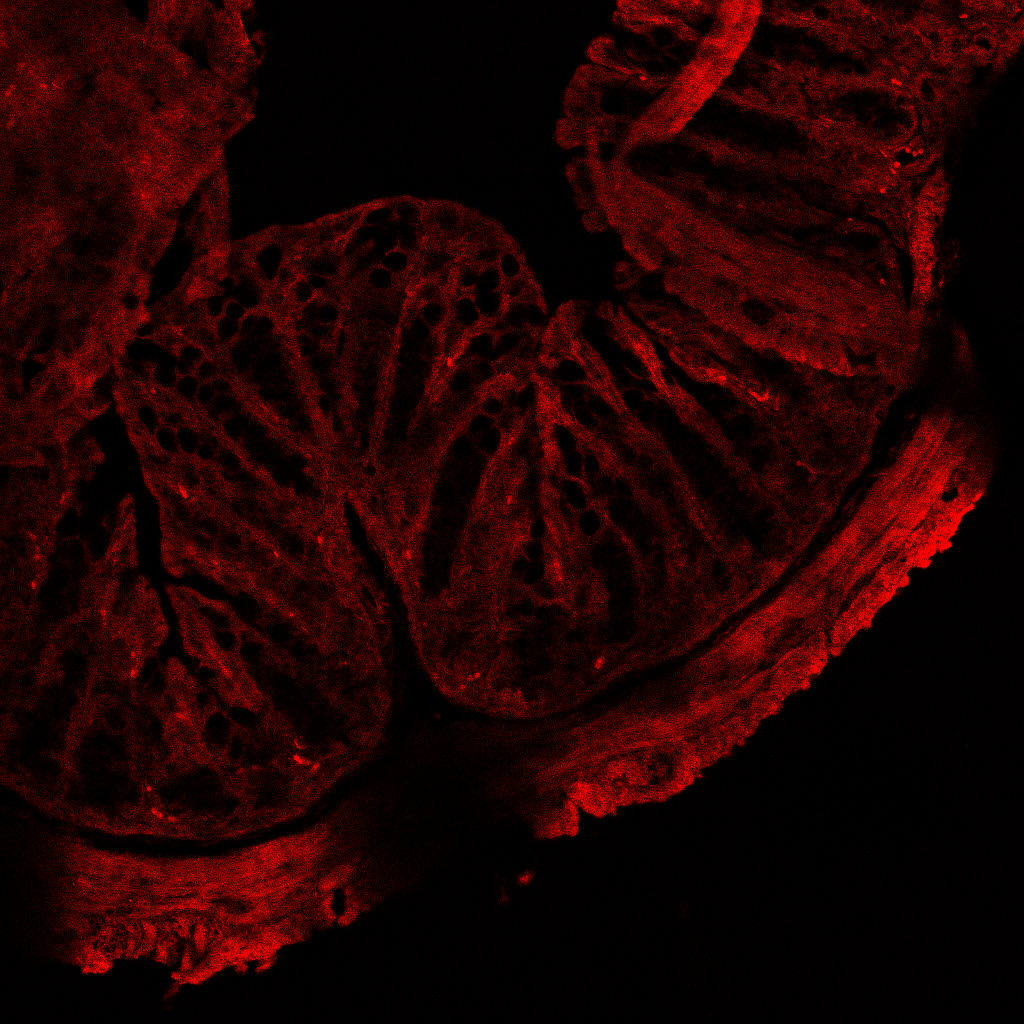

Supplement: Supplementary file 10 [file Data_Sheet_10.ZIP › Figure5/LGG_C002T001.tif]

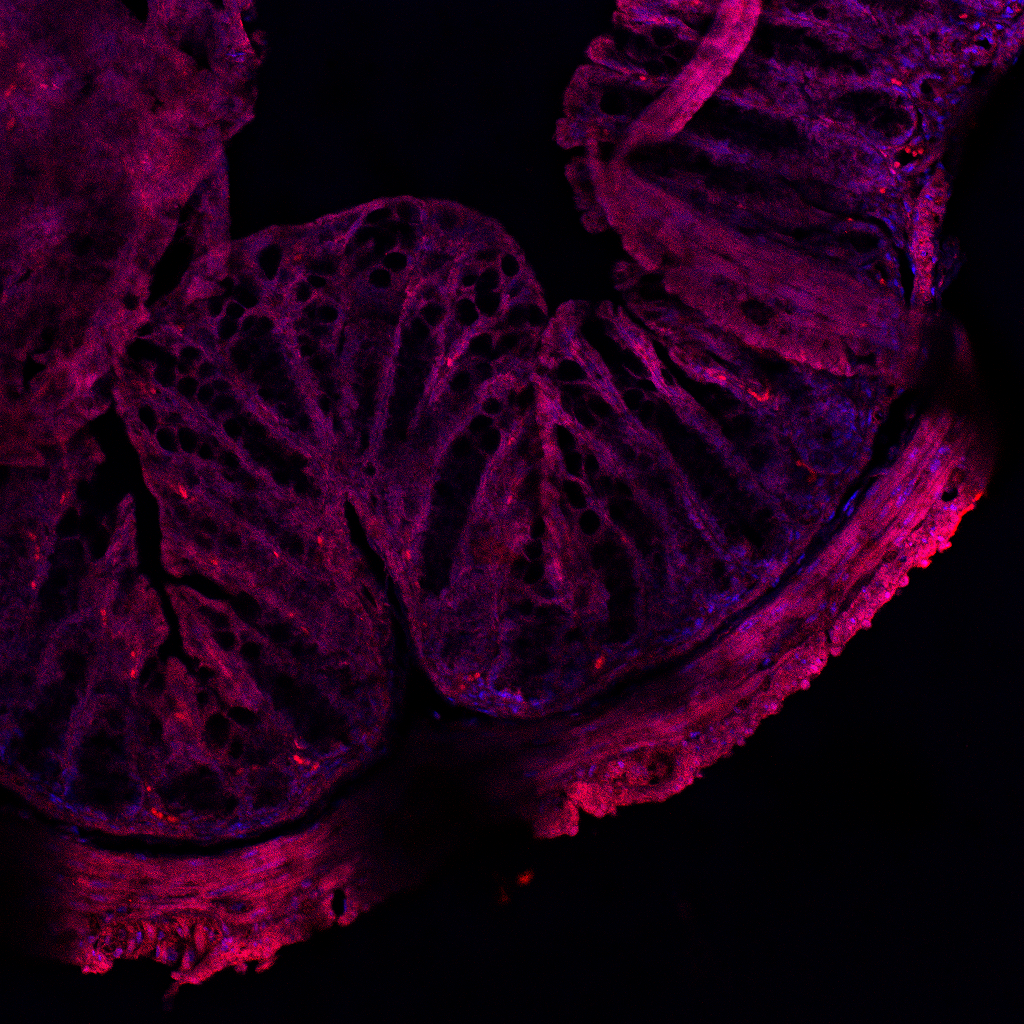

Supplement: Supplementary file 10 [file Data_Sheet_10.ZIP › Figure5/LGG_T001.tif]
